# Supplementary figures and images for: Histone H3K9 demethylase JMJD2B/KDM4B promotes osteogenic differentiation of bone marrow-derived mesenchymal stem cells by regulating H3K9me2 on RUNX2
Source: PeerJ. 2022 Oct 5;10:e13862. doi: 10.7717/peerj.13862 (PMC9547583; doi:10.7717/peerj.13862)

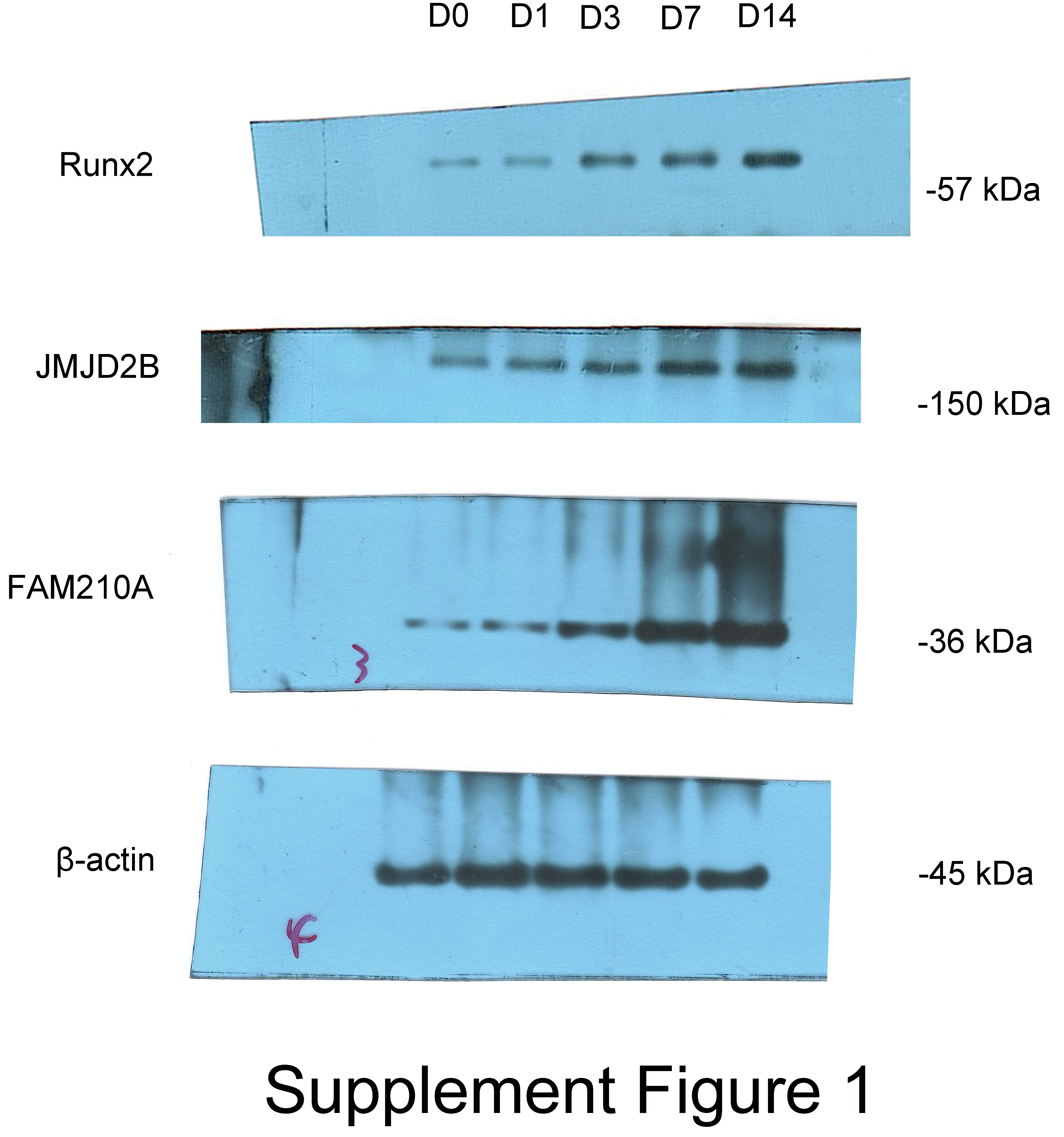

Supplement: Supplemental Information 1 [file peerj-10-13862-s001.jpg]

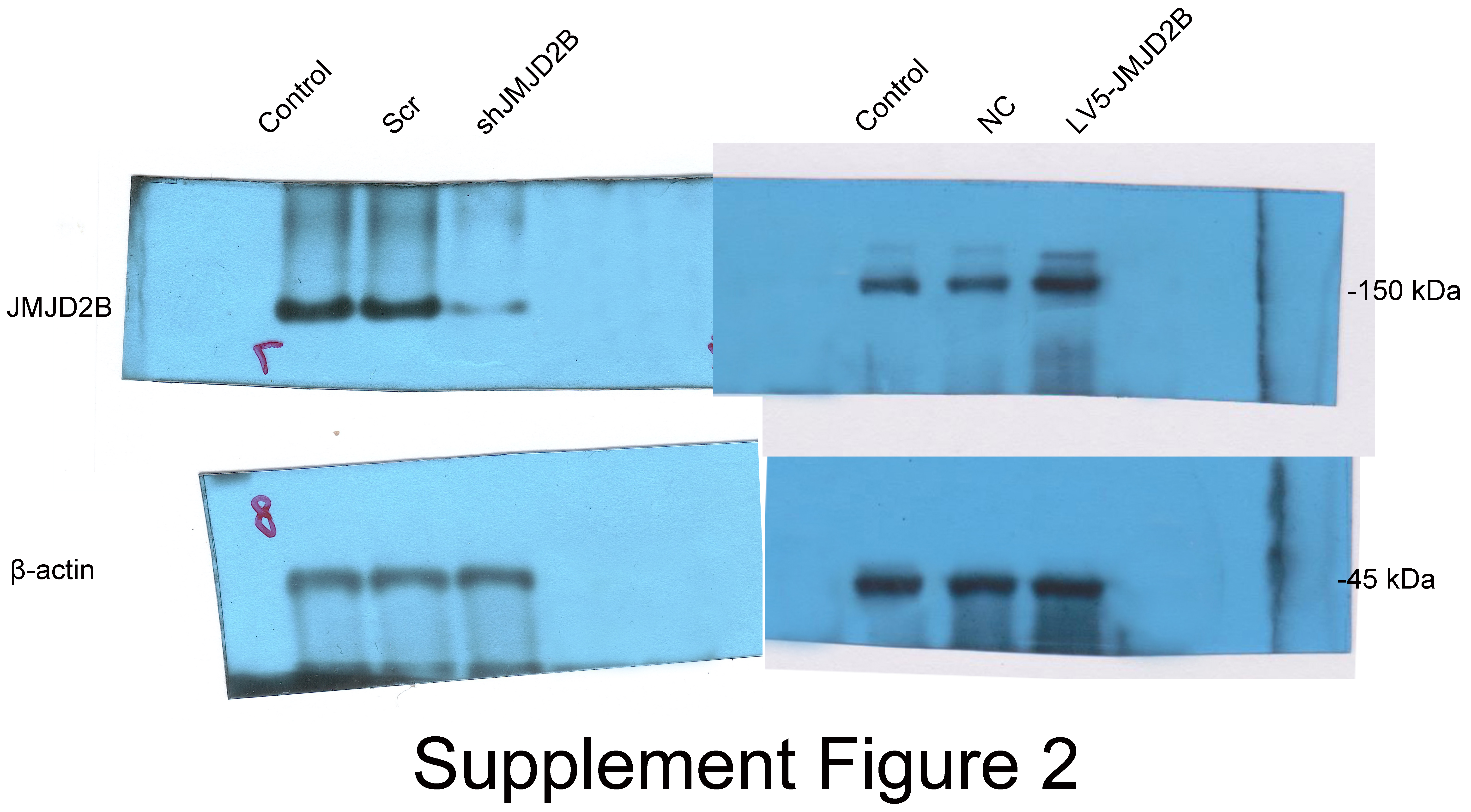

Supplement: Supplemental Information 2 [file peerj-10-13862-s002.jpg]

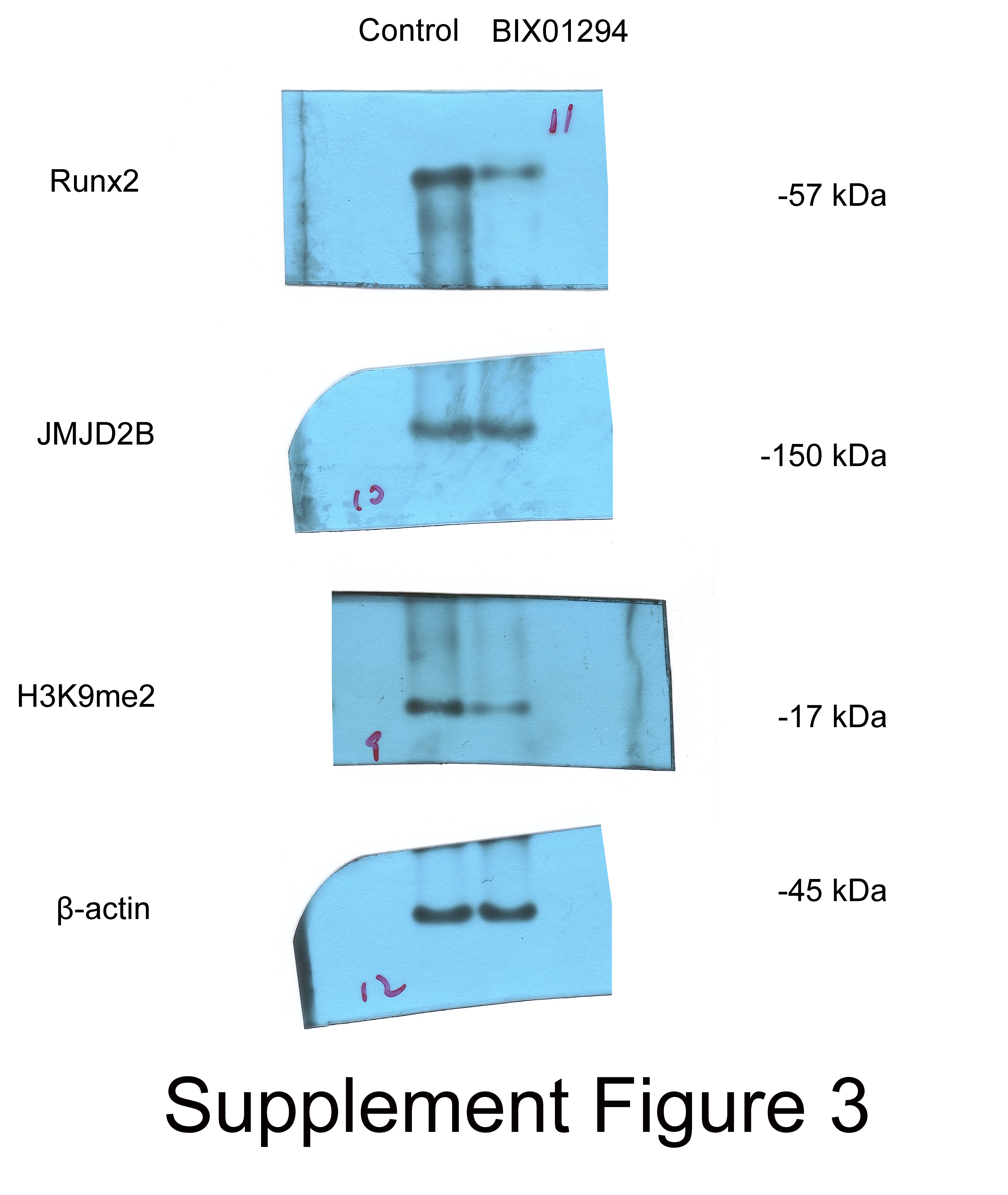

Supplement: Supplemental Information 3 [file peerj-10-13862-s003.jpg]

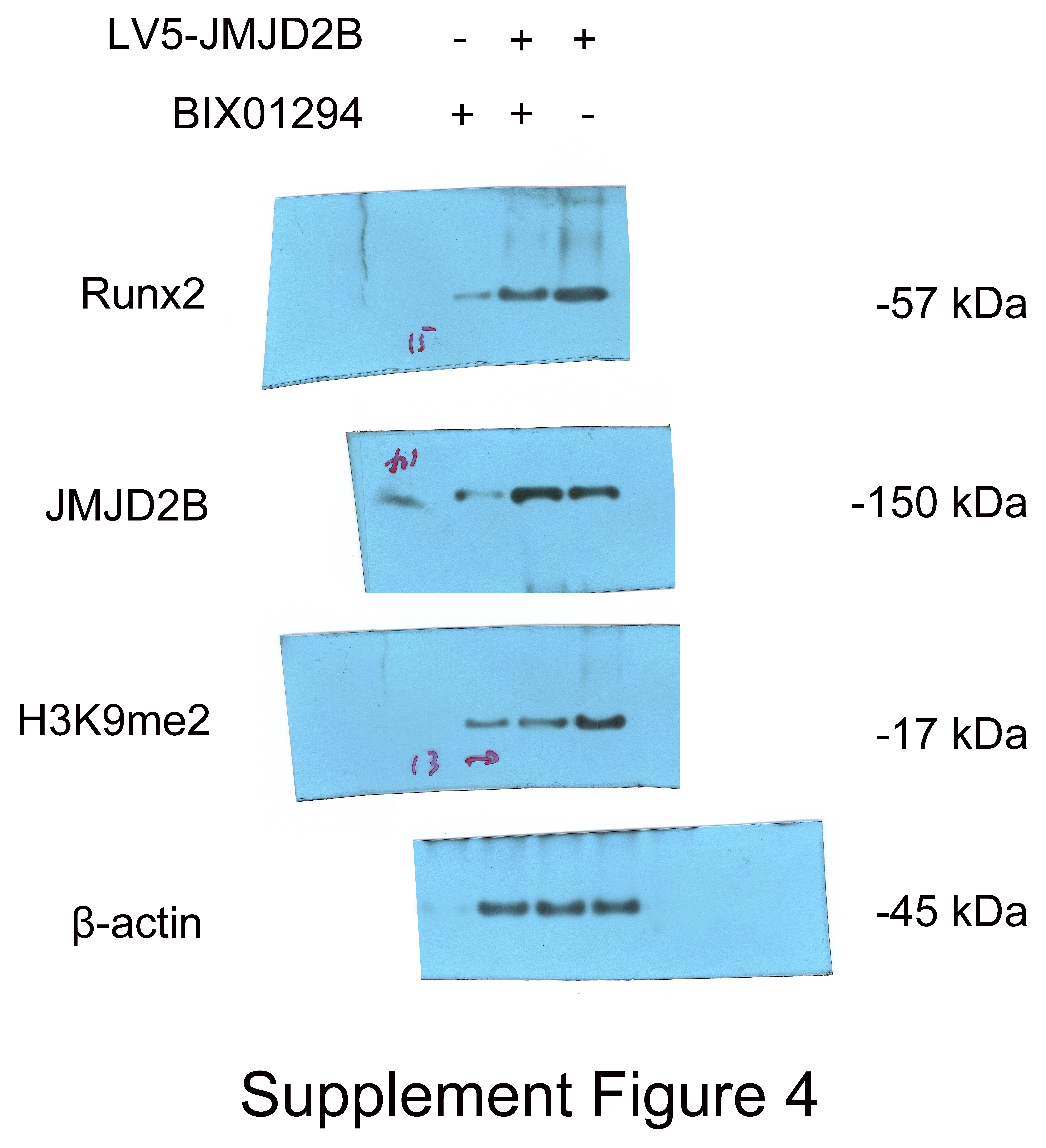

Supplement: Supplemental Information 4 [file peerj-10-13862-s004.jpg]

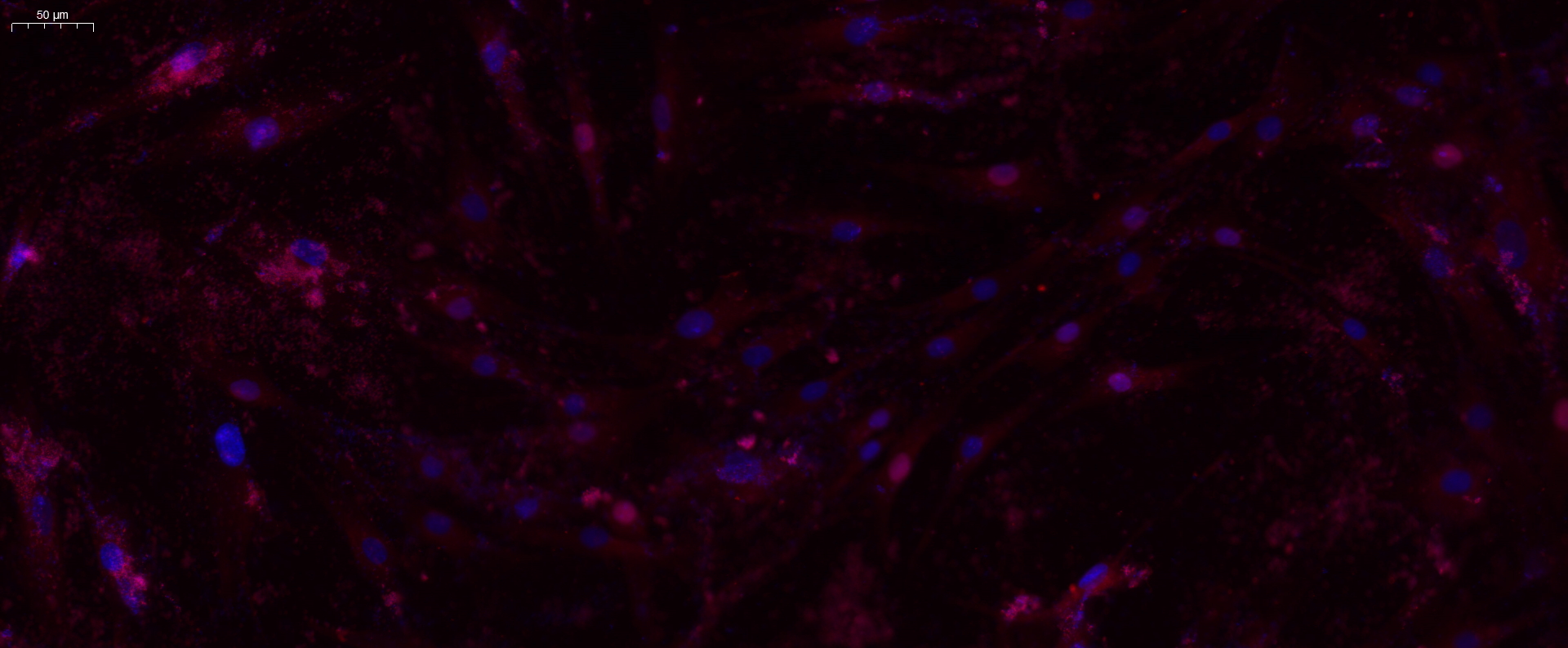

Supplement: Supplemental Information 5 [file peerj-10-13862-s005.zip › Supplement File 1(Figure 1C FAM210)/FAM 14d 2.jpg]

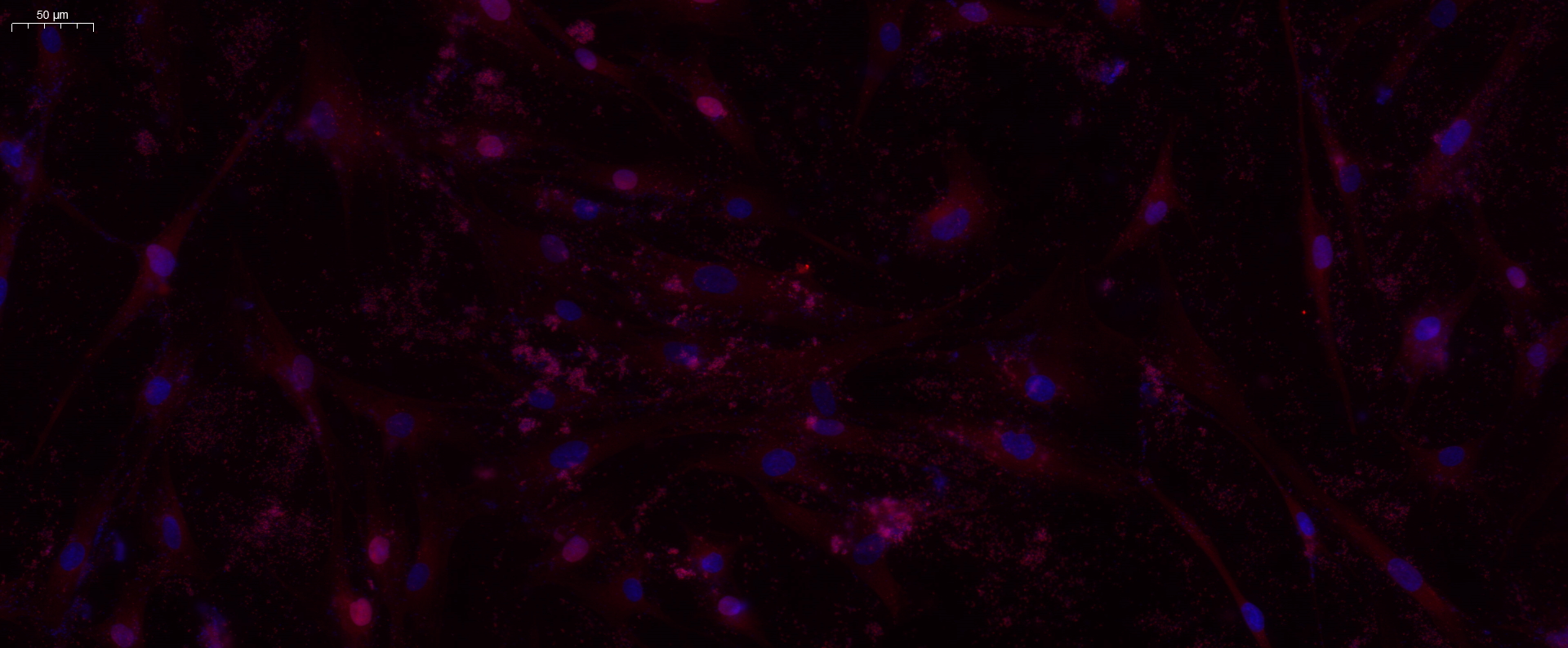

Supplement: Supplemental Information 5 [file peerj-10-13862-s005.zip › Supplement File 1(Figure 1C FAM210)/FAM 14d 1.jpg]

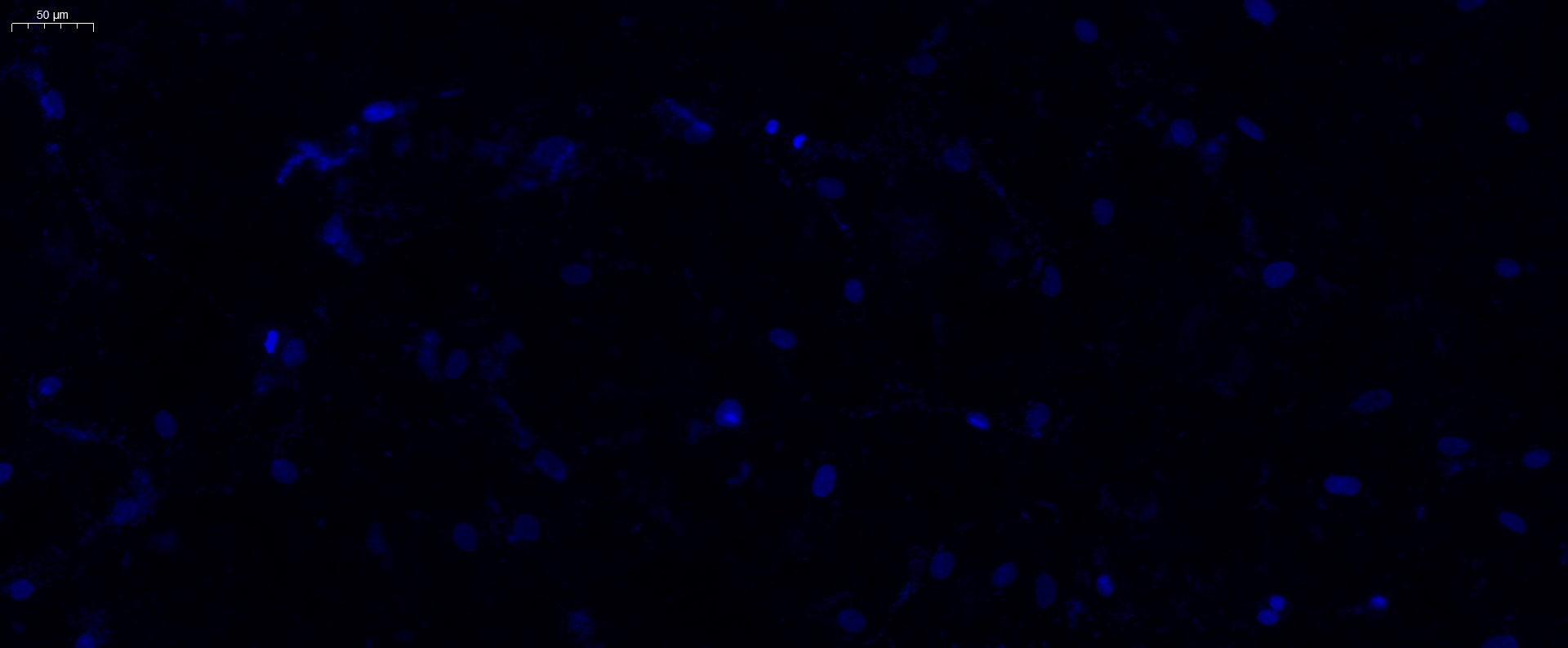

Supplement: Supplemental Information 5 [file peerj-10-13862-s005.zip › Supplement File 1(Figure 1C FAM210)/FAM 3d 2 1.jpg]

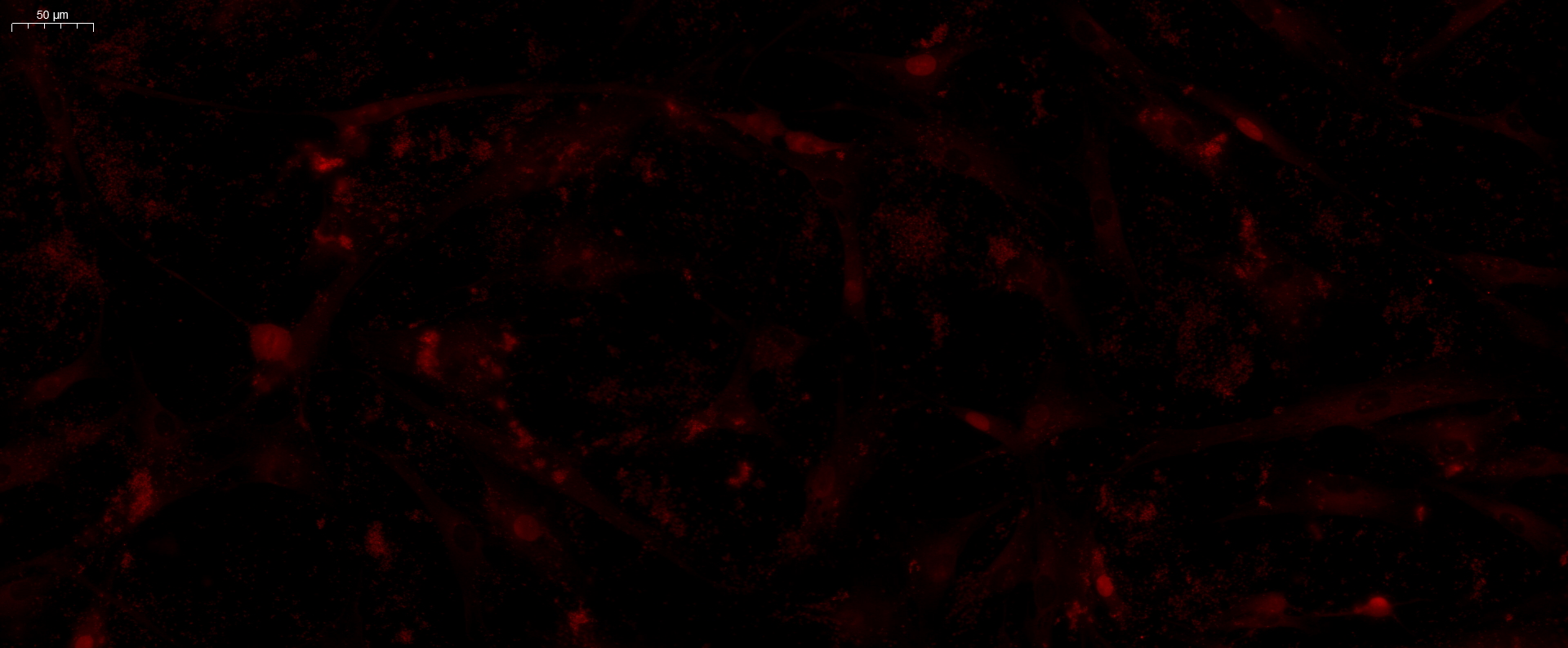

Supplement: Supplemental Information 5 [file peerj-10-13862-s005.zip › Supplement File 1(Figure 1C FAM210)/FAM 3d 2 2.jpg]

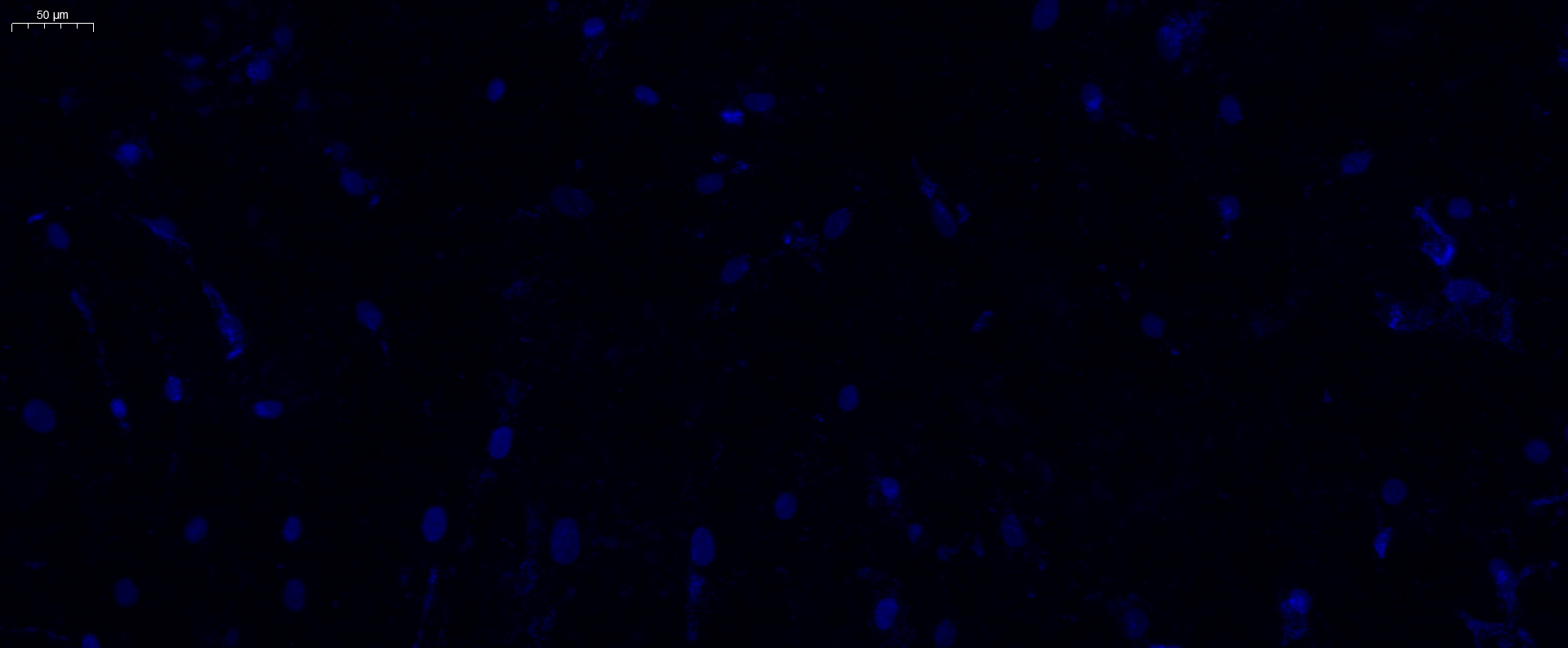

Supplement: Supplemental Information 5 [file peerj-10-13862-s005.zip › Supplement File 1(Figure 1C FAM210)/FAM 3d 1 1.jpg]

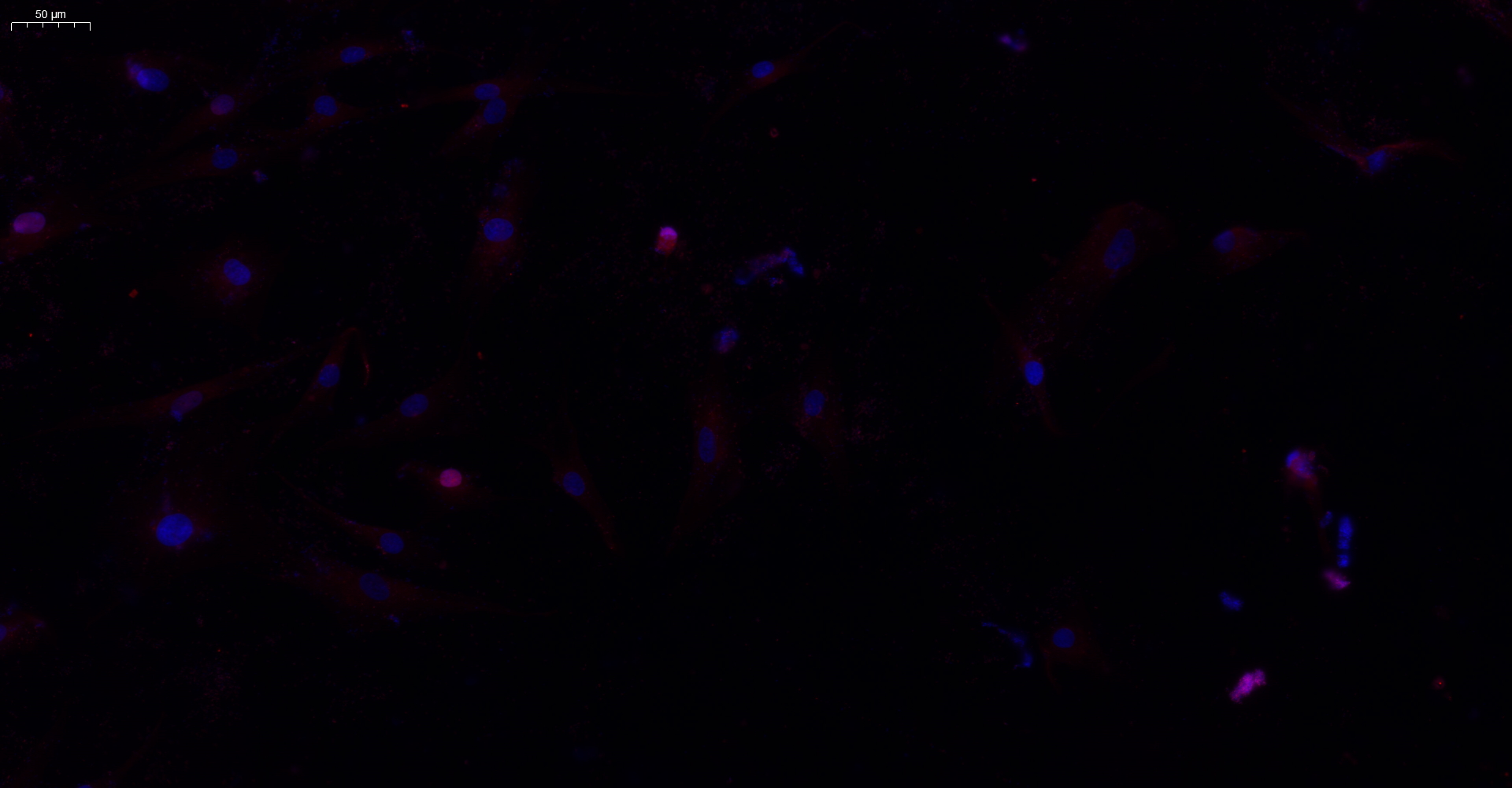

Supplement: Supplemental Information 5 [file peerj-10-13862-s005.zip › Supplement File 1(Figure 1C FAM210)/FAM 0d 2.jpg]

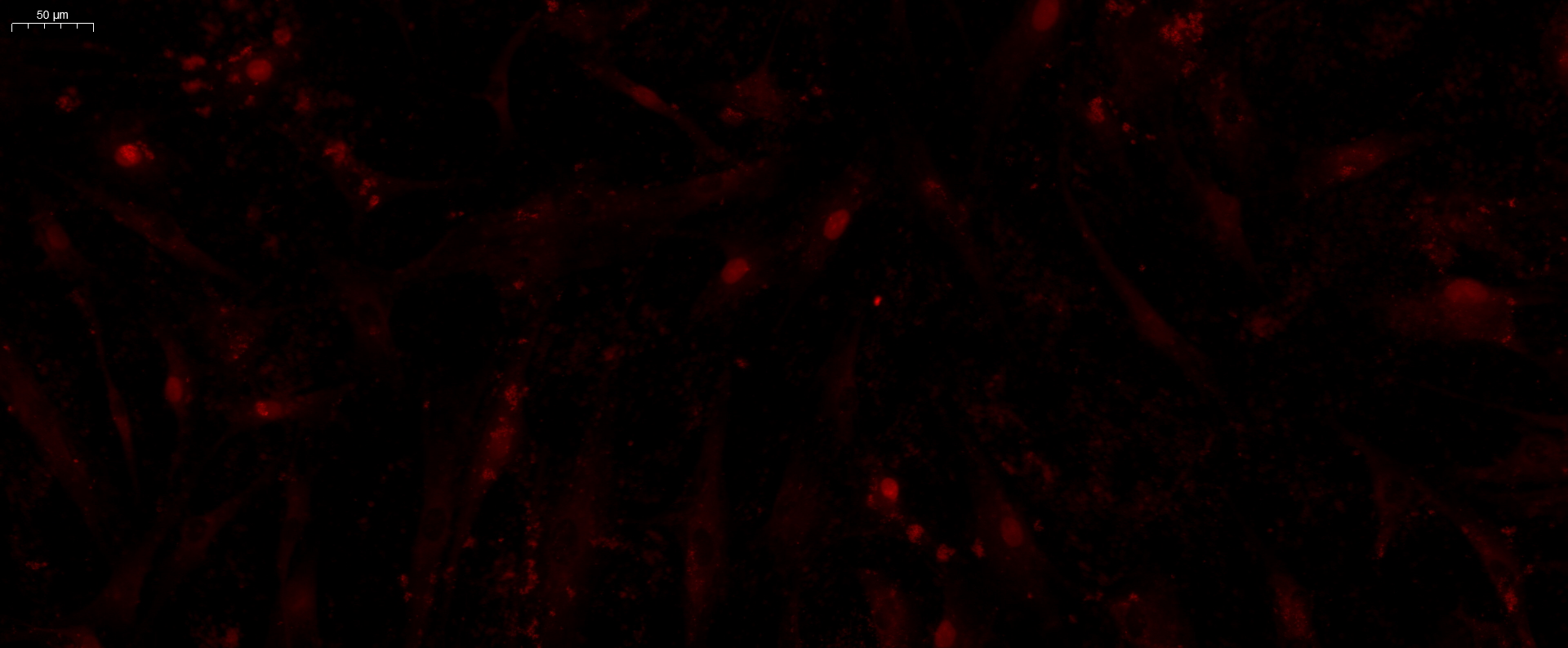

Supplement: Supplemental Information 5 [file peerj-10-13862-s005.zip › Supplement File 1(Figure 1C FAM210)/FAM 3d 1 2.jpg]

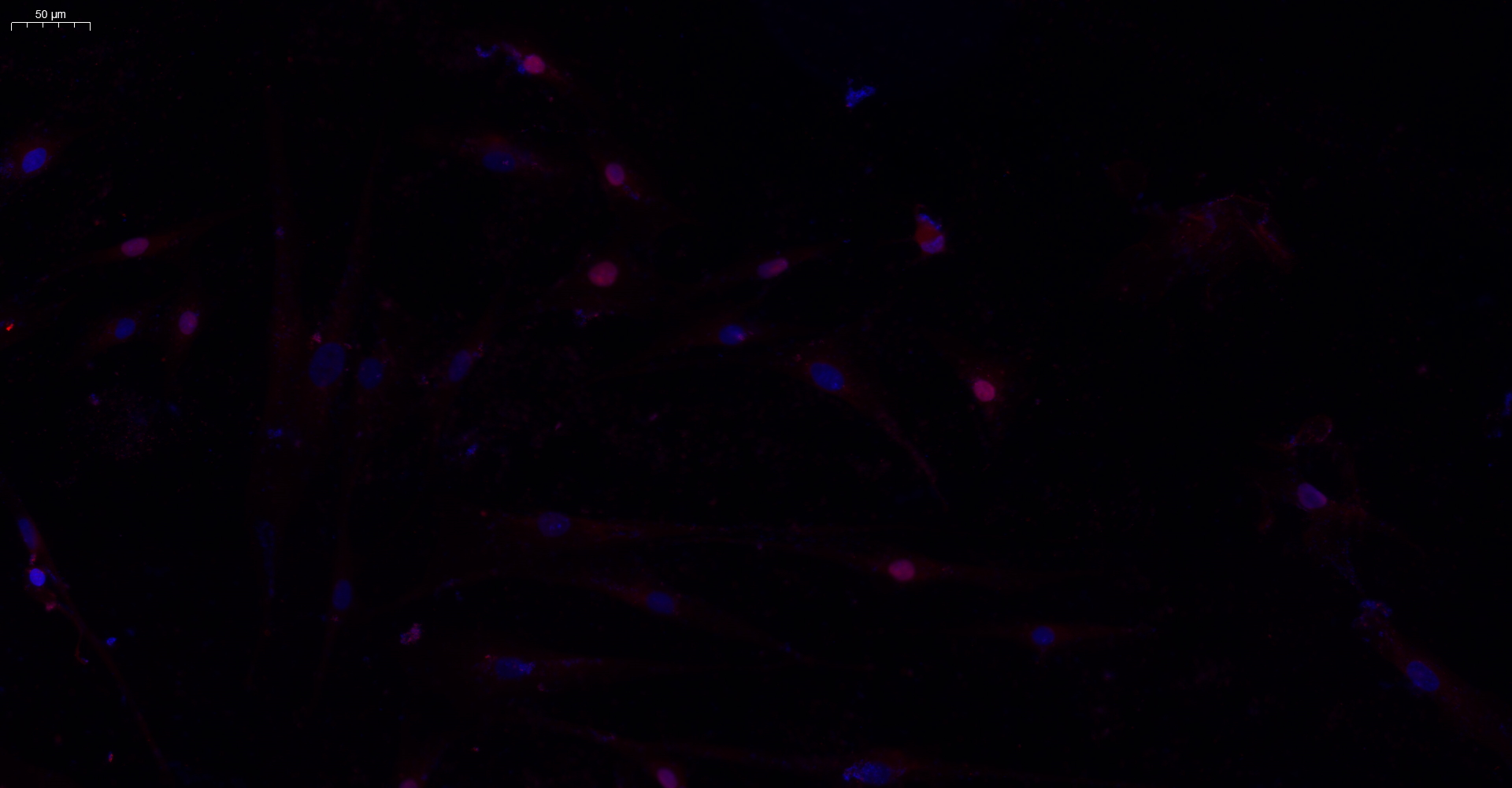

Supplement: Supplemental Information 5 [file peerj-10-13862-s005.zip › Supplement File 1(Figure 1C FAM210)/FAM 0d 1.jpg]

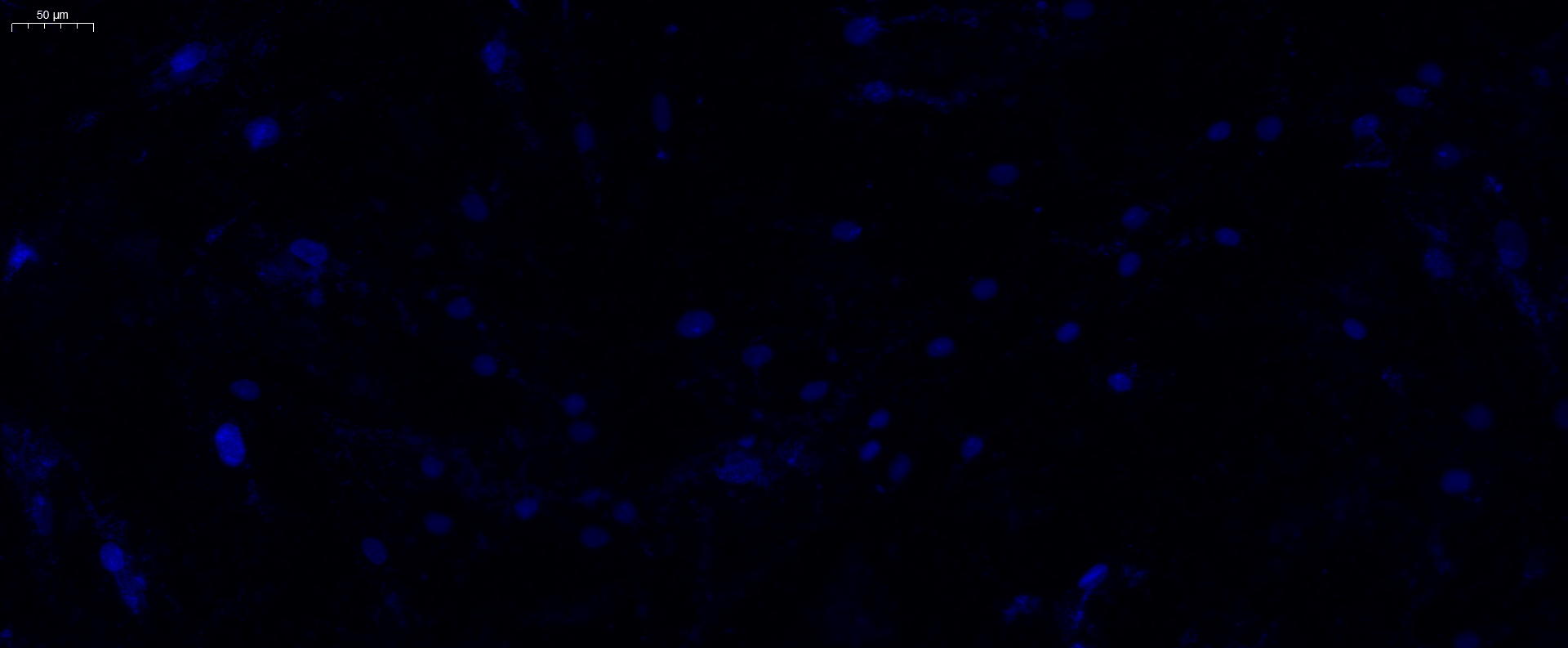

Supplement: Supplemental Information 5 [file peerj-10-13862-s005.zip › Supplement File 1(Figure 1C FAM210)/FAM 14d 2 1.jpg]

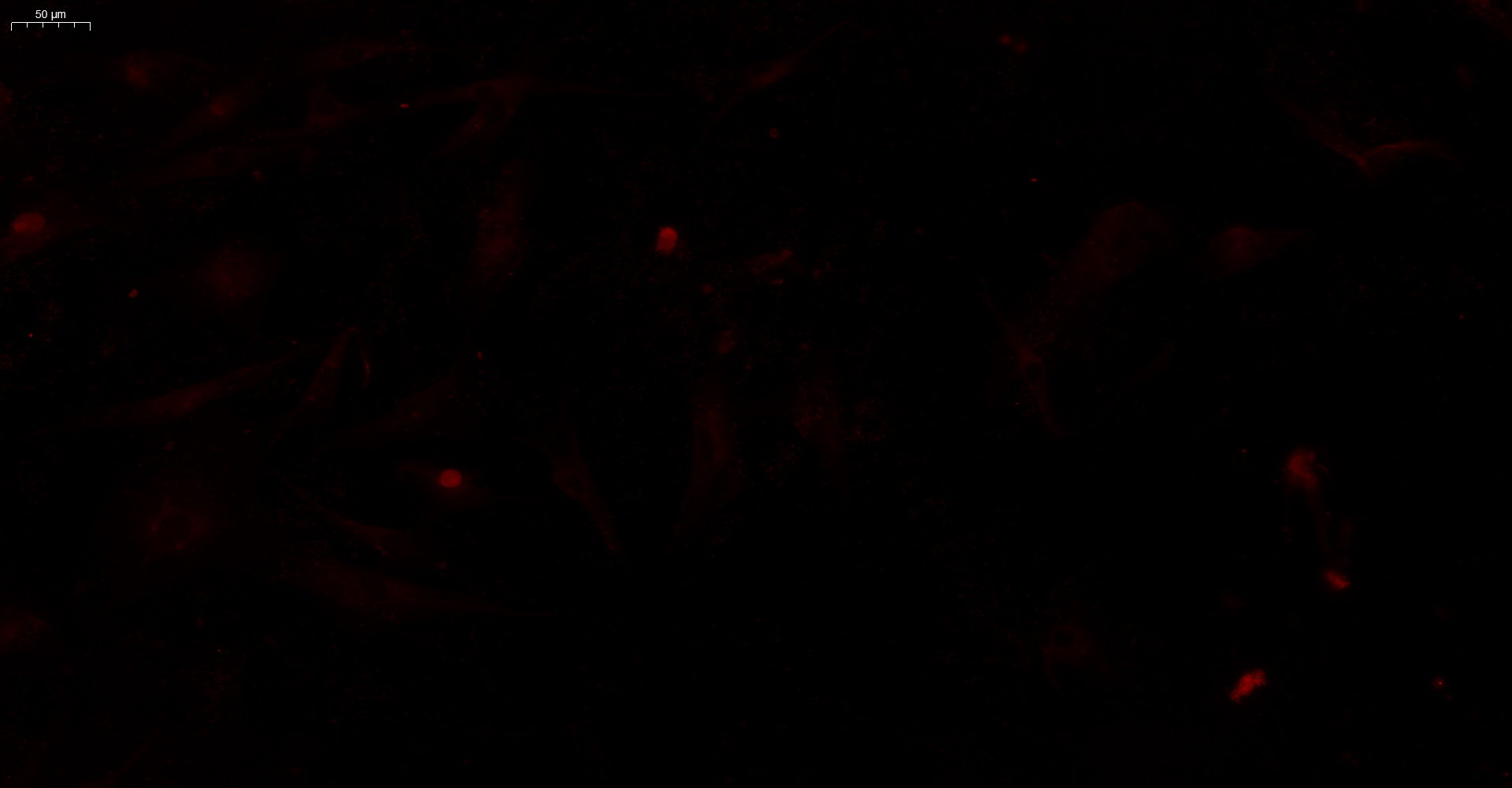

Supplement: Supplemental Information 5 [file peerj-10-13862-s005.zip › Supplement File 1(Figure 1C FAM210)/FAM 0d 2 2.jpg]

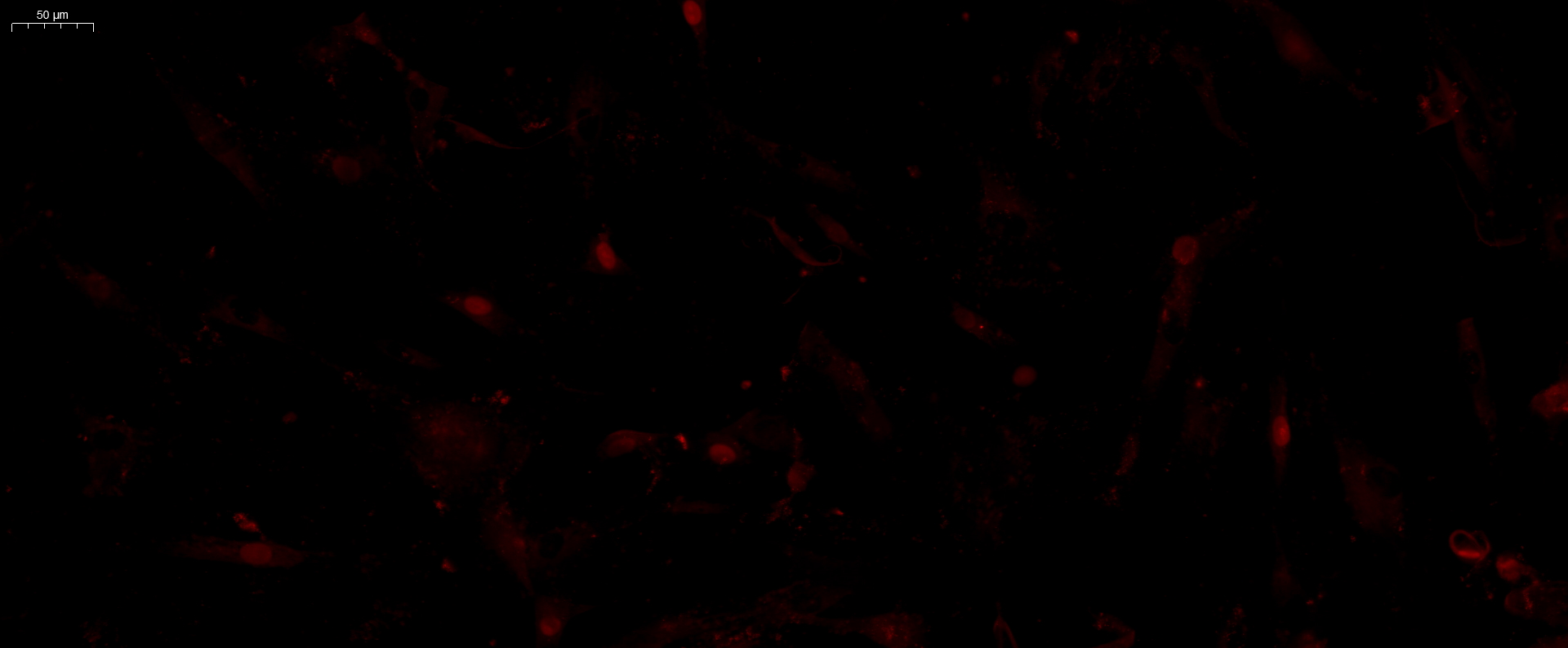

Supplement: Supplemental Information 5 [file peerj-10-13862-s005.zip › Supplement File 1(Figure 1C FAM210)/FAM 1d 2 2.jpg]

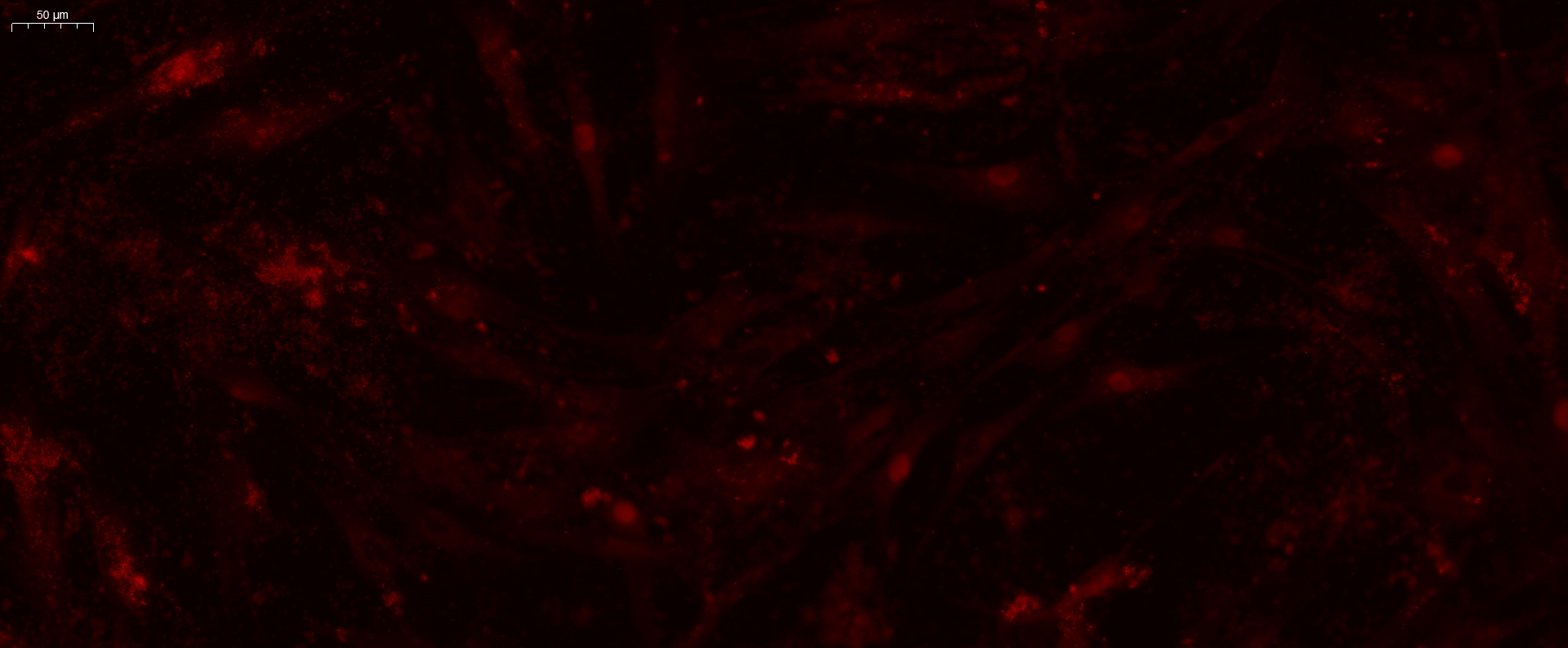

Supplement: Supplemental Information 5 [file peerj-10-13862-s005.zip › Supplement File 1(Figure 1C FAM210)/FAM 14d 2 2.jpg]

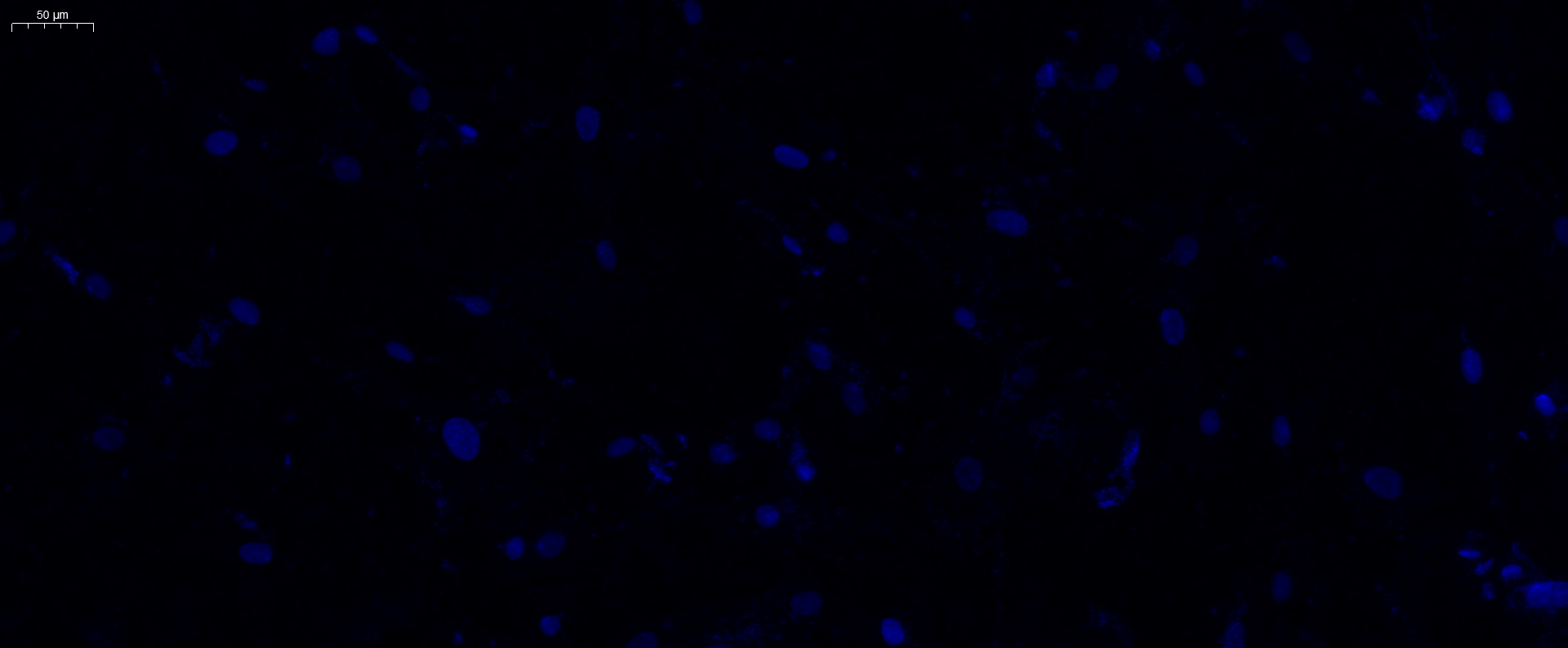

Supplement: Supplemental Information 5 [file peerj-10-13862-s005.zip › Supplement File 1(Figure 1C FAM210)/FAM 1d 2 1.jpg]

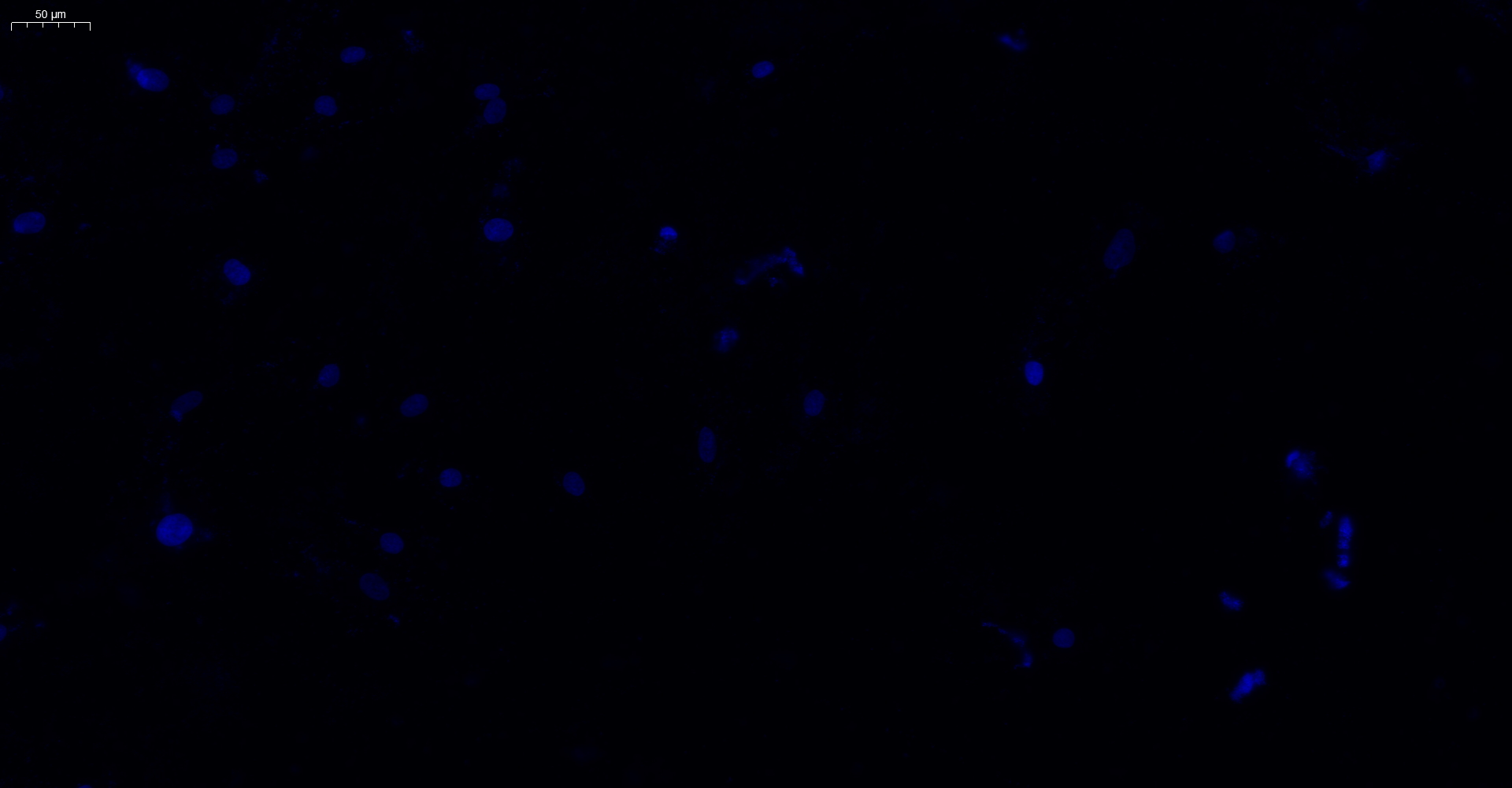

Supplement: Supplemental Information 5 [file peerj-10-13862-s005.zip › Supplement File 1(Figure 1C FAM210)/FAM 0d 2 1.jpg]

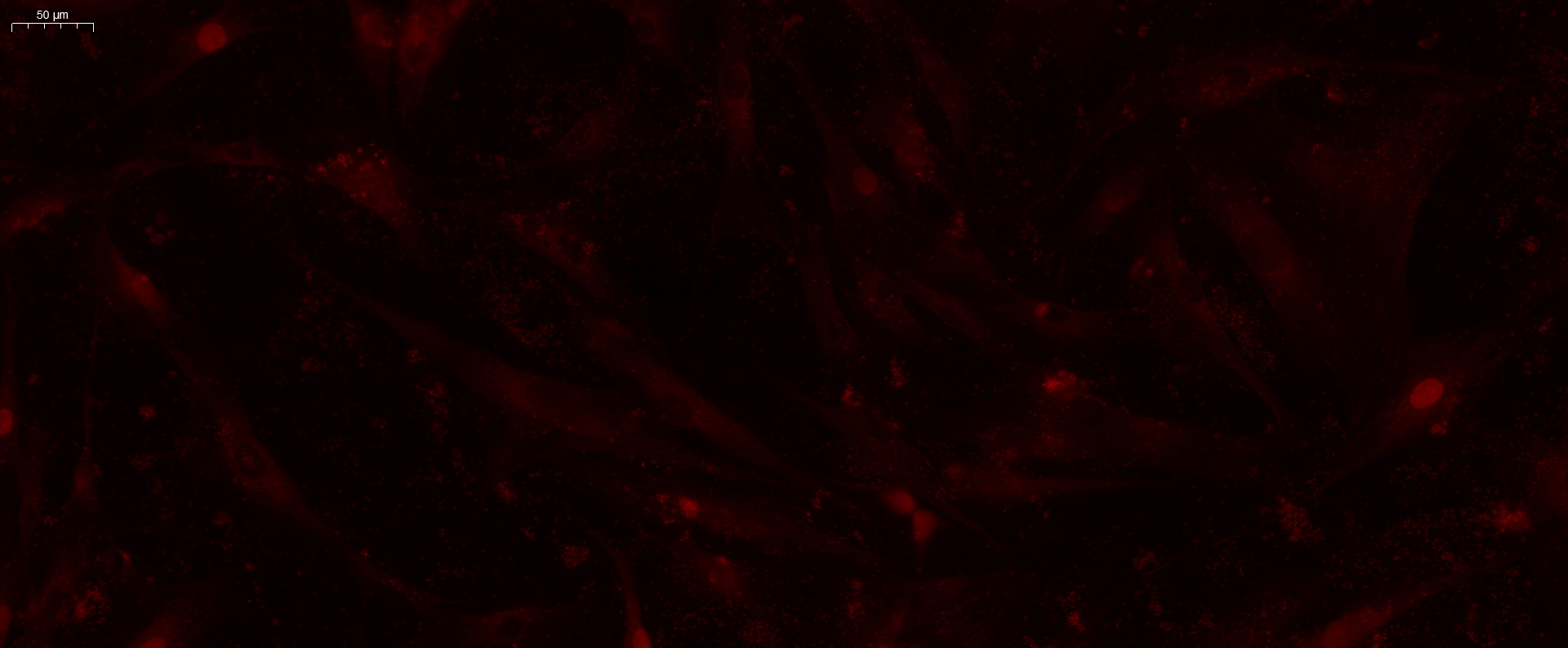

Supplement: Supplemental Information 5 [file peerj-10-13862-s005.zip › Supplement File 1(Figure 1C FAM210)/FAM 7d 1 2.jpg]

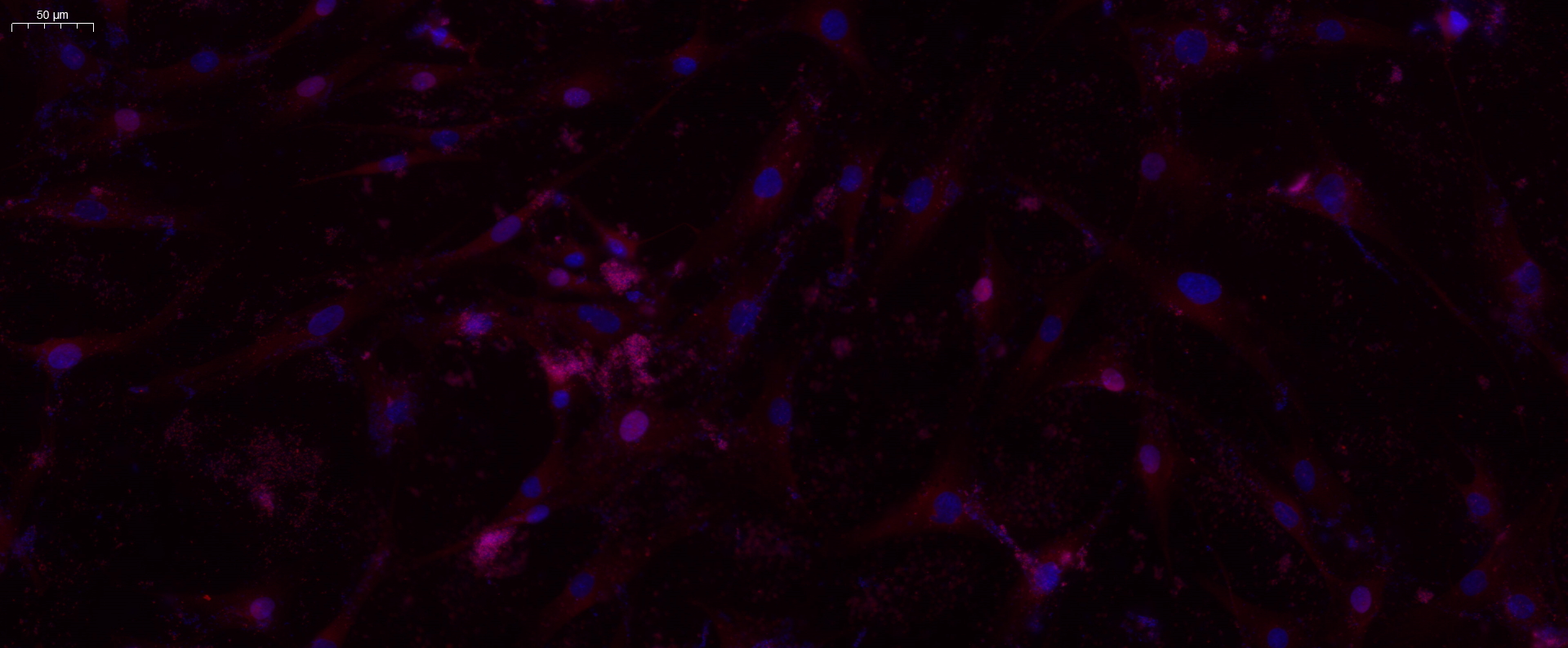

Supplement: Supplemental Information 5 [file peerj-10-13862-s005.zip › Supplement File 1(Figure 1C FAM210)/FAM 7d 2.jpg]

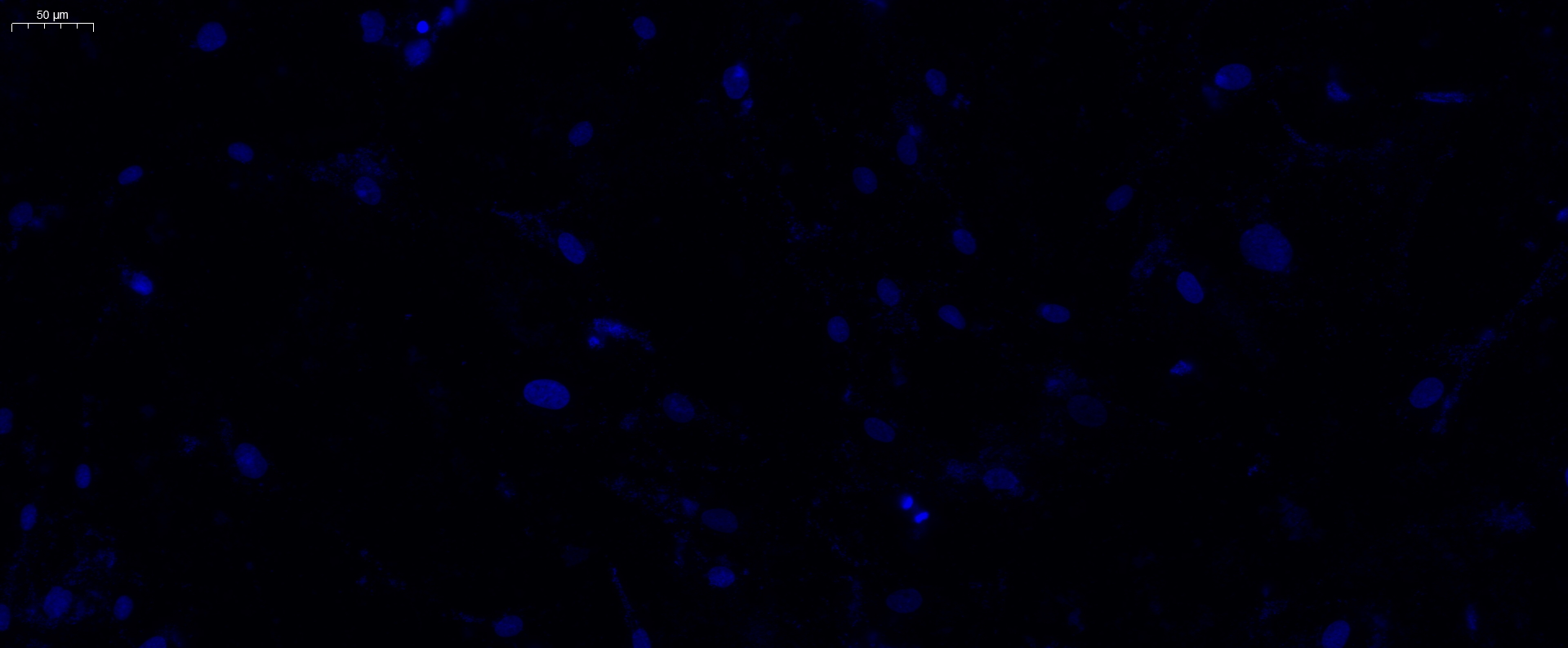

Supplement: Supplemental Information 5 [file peerj-10-13862-s005.zip › Supplement File 1(Figure 1C FAM210)/FAM 7d 1 1.jpg]

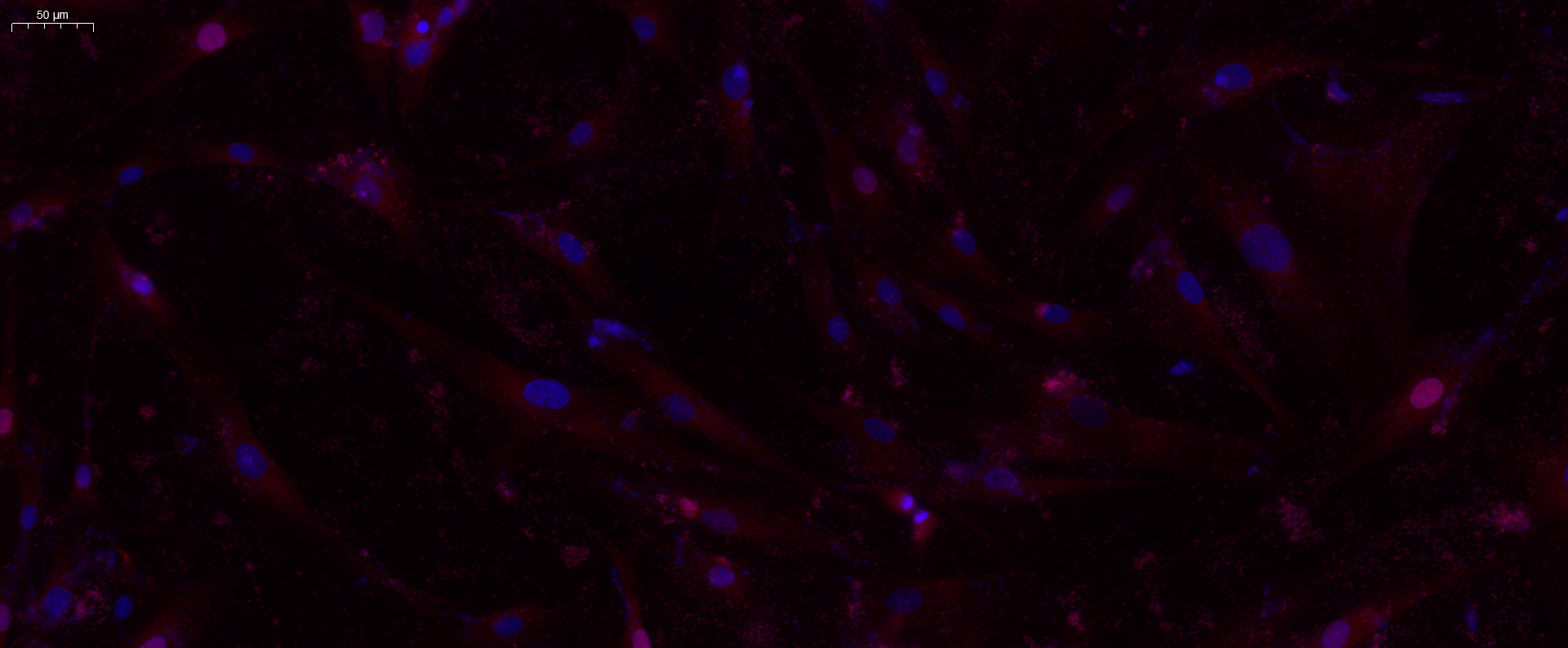

Supplement: Supplemental Information 5 [file peerj-10-13862-s005.zip › Supplement File 1(Figure 1C FAM210)/FAM 7d 1.jpg]

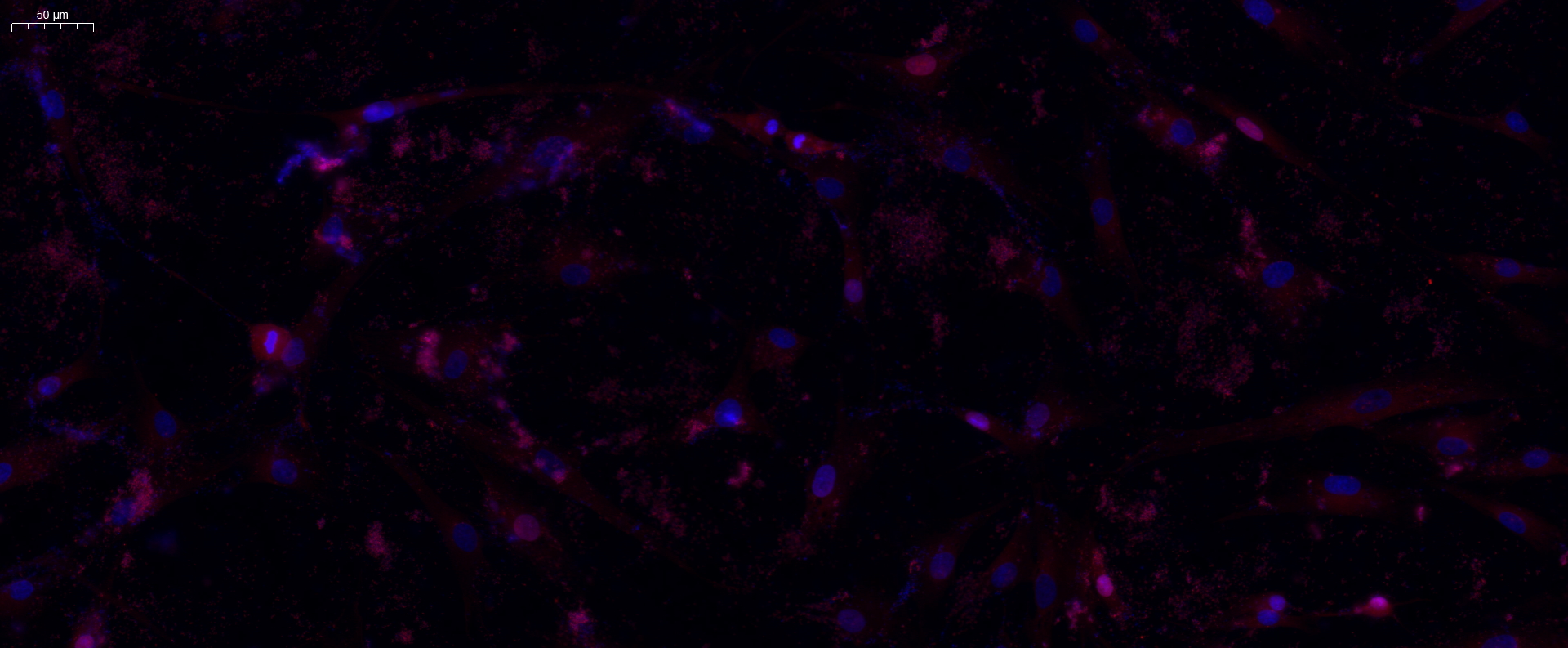

Supplement: Supplemental Information 5 [file peerj-10-13862-s005.zip › Supplement File 1(Figure 1C FAM210)/FAM 3d 2.jpg]

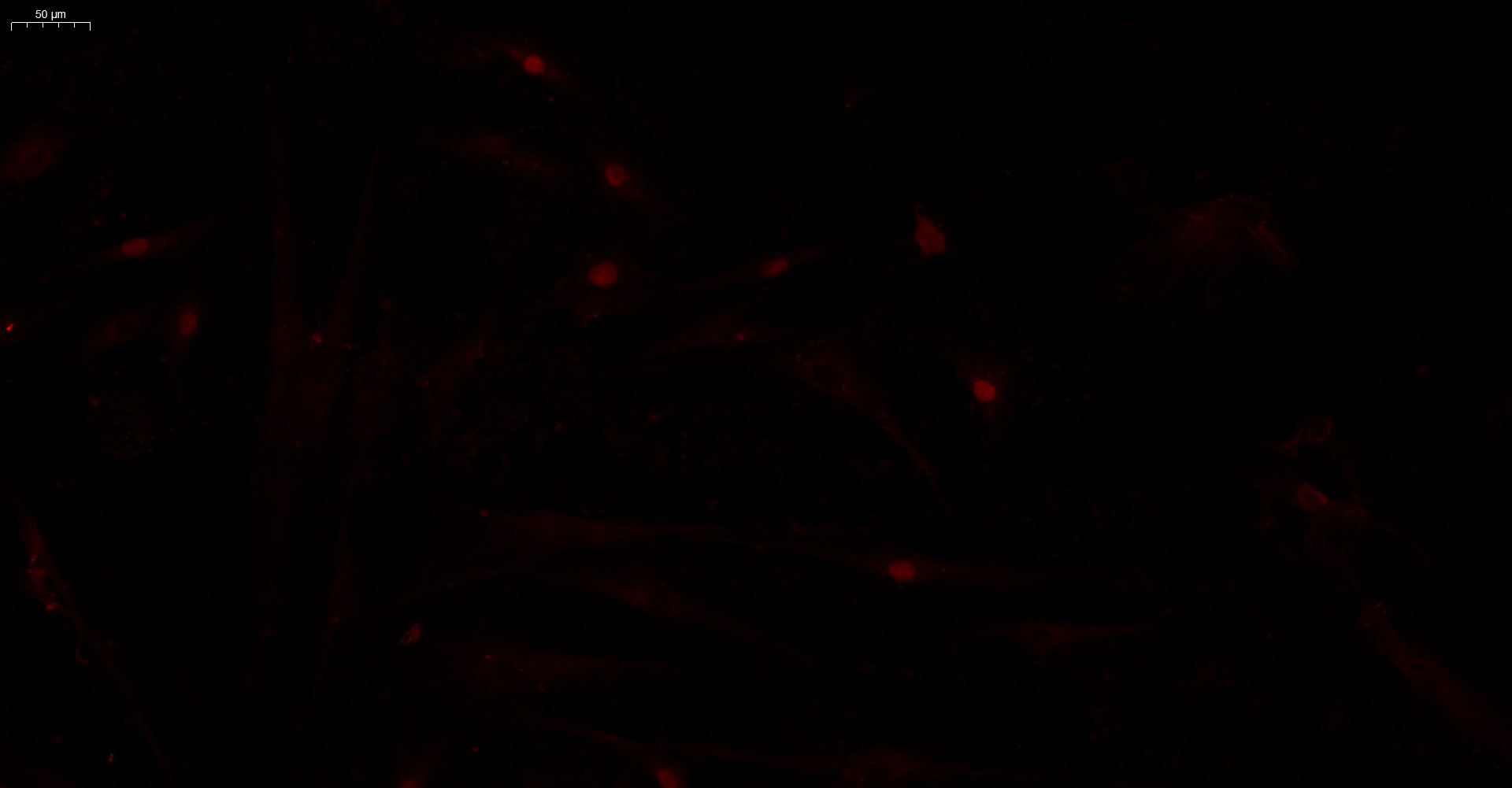

Supplement: Supplemental Information 5 [file peerj-10-13862-s005.zip › Supplement File 1(Figure 1C FAM210)/FAM 0d 1 2.jpg]

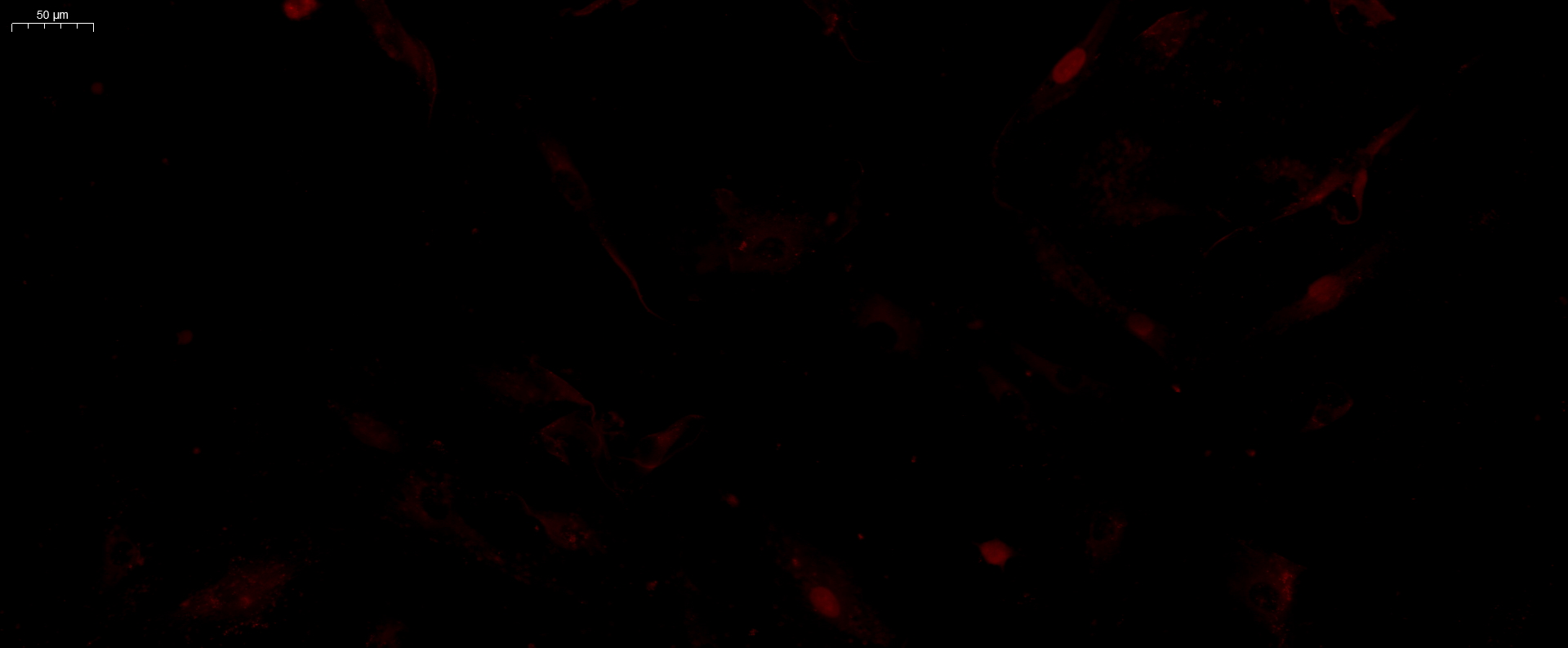

Supplement: Supplemental Information 5 [file peerj-10-13862-s005.zip › Supplement File 1(Figure 1C FAM210)/FAM 1d 1 2.jpg]

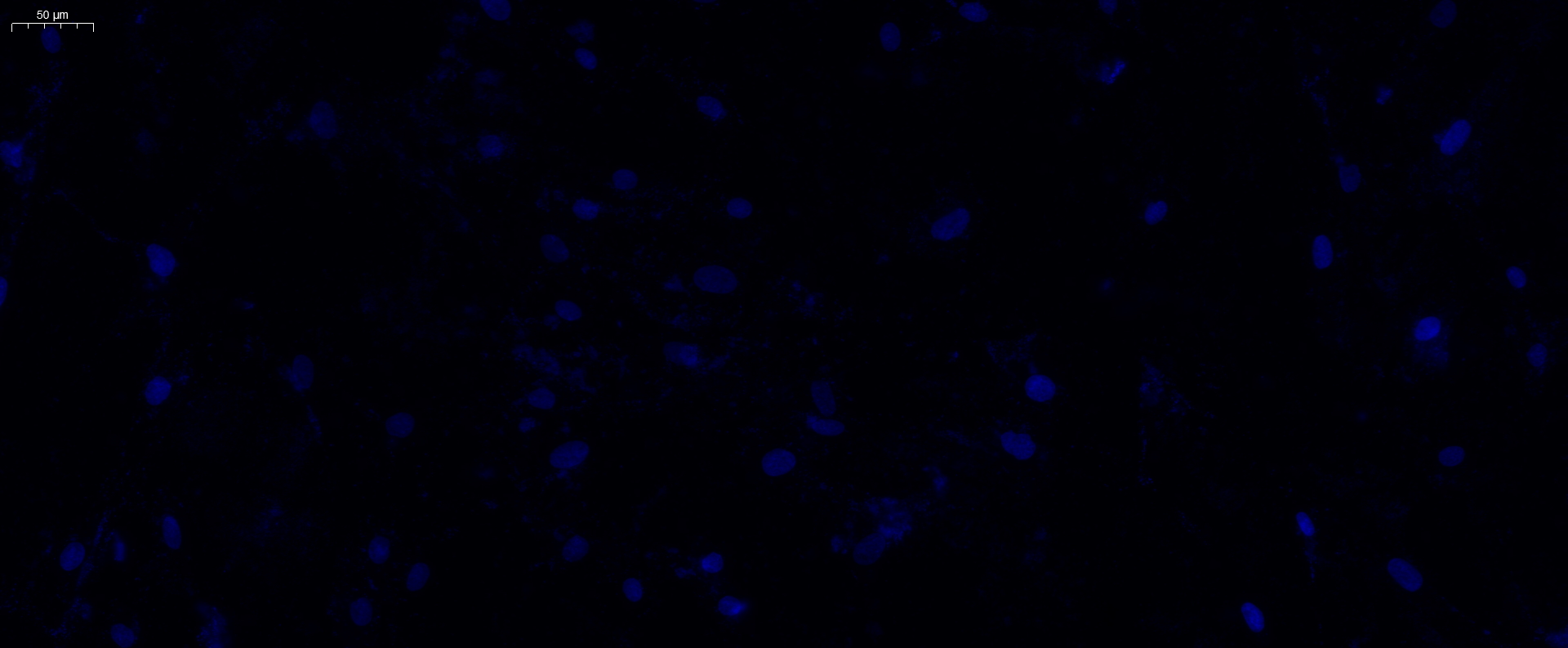

Supplement: Supplemental Information 5 [file peerj-10-13862-s005.zip › Supplement File 1(Figure 1C FAM210)/FAM 14d 1 1.jpg]

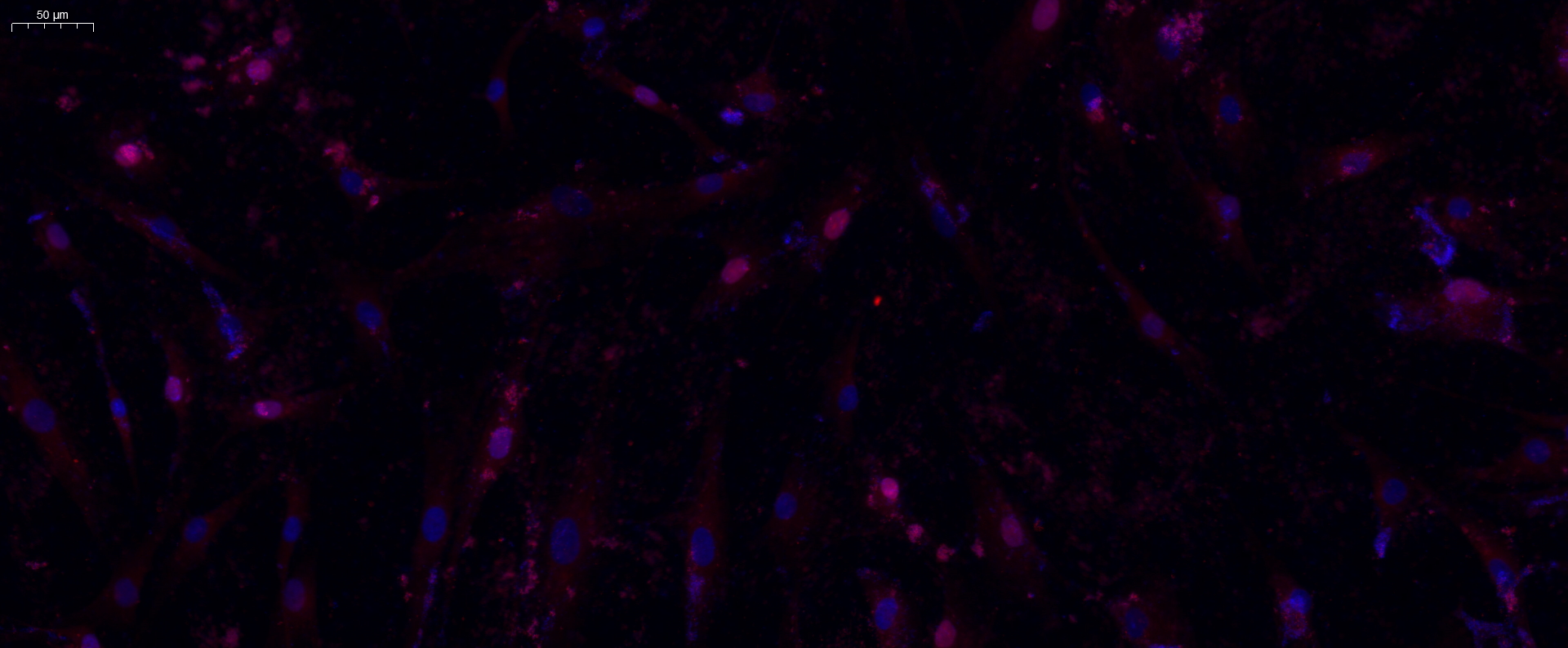

Supplement: Supplemental Information 5 [file peerj-10-13862-s005.zip › Supplement File 1(Figure 1C FAM210)/FAM 3d 1.jpg]

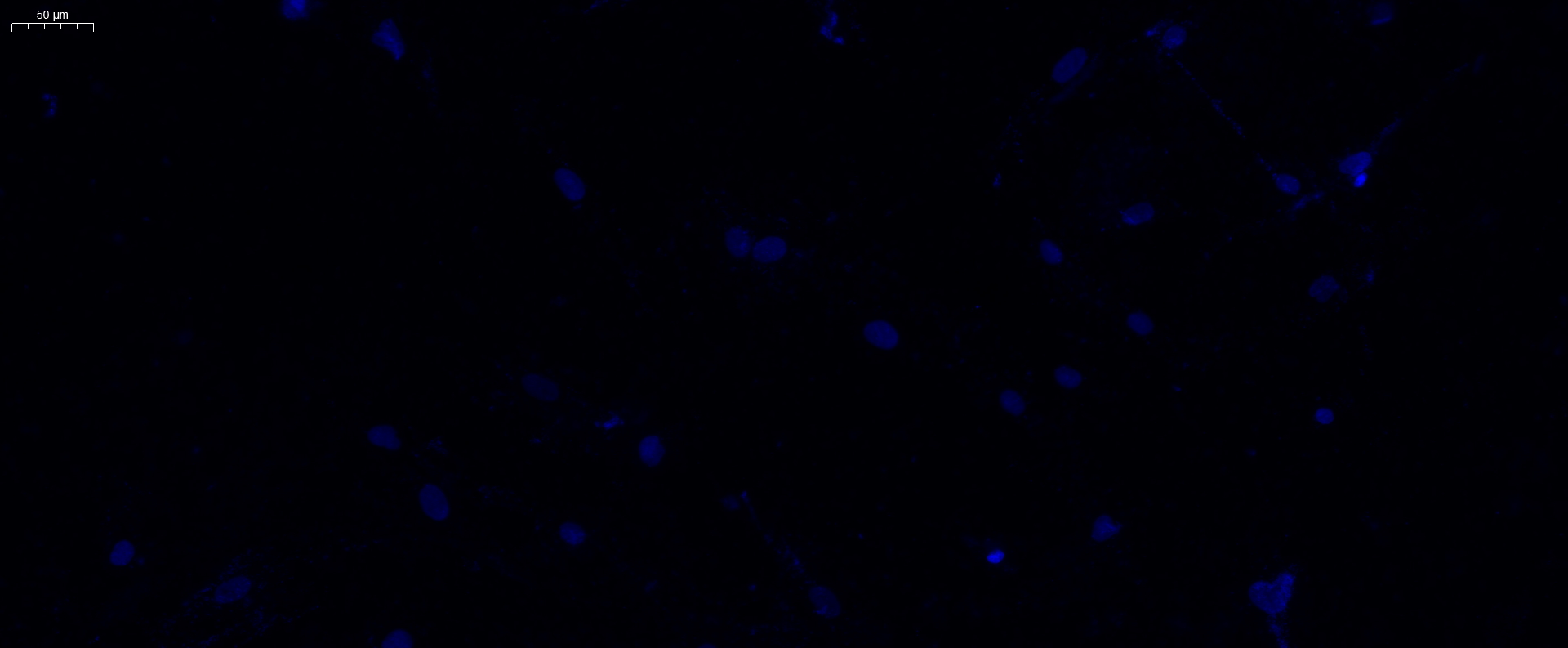

Supplement: Supplemental Information 5 [file peerj-10-13862-s005.zip › Supplement File 1(Figure 1C FAM210)/FAM 1d 1 1.jpg]

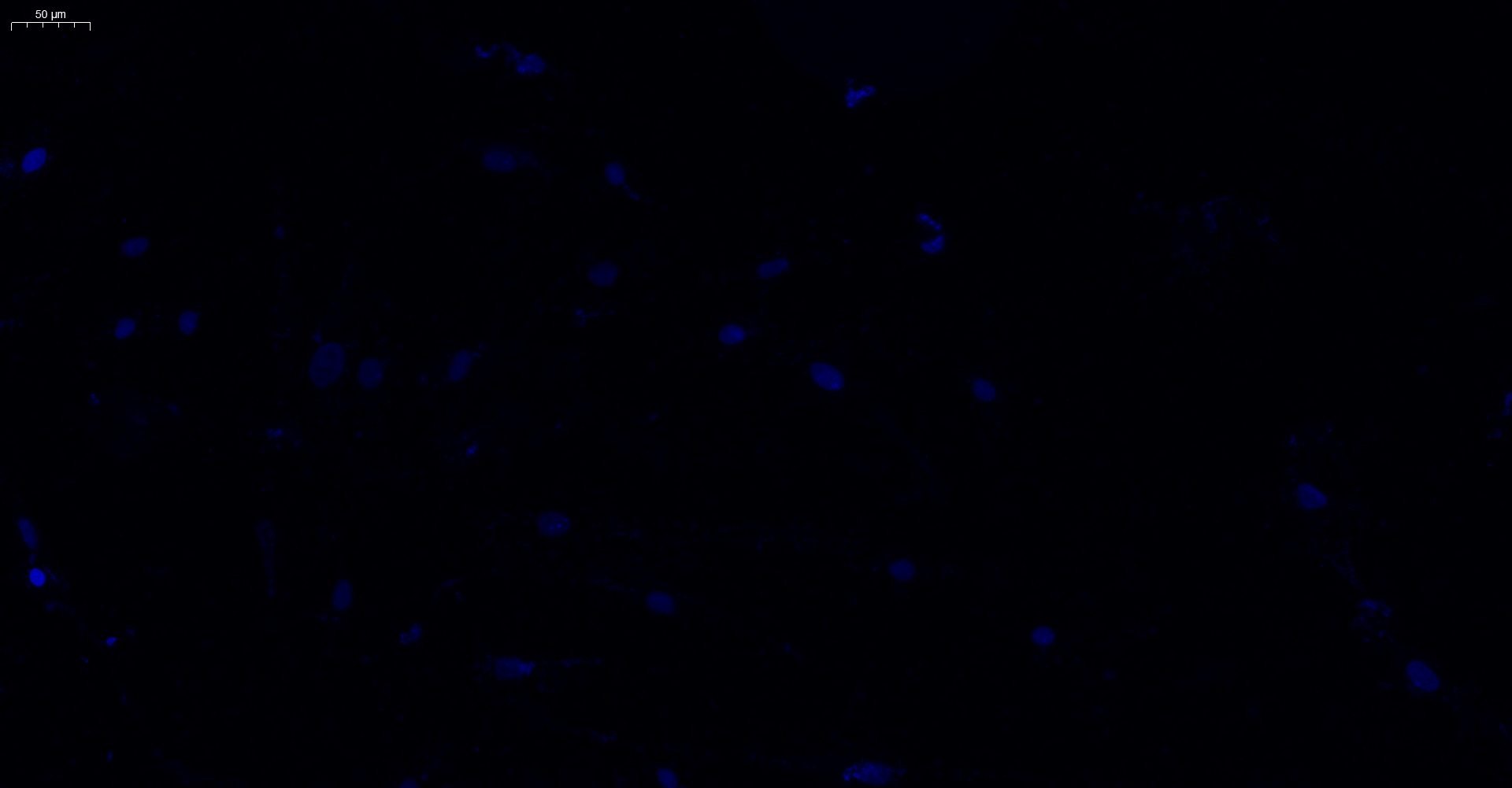

Supplement: Supplemental Information 5 [file peerj-10-13862-s005.zip › Supplement File 1(Figure 1C FAM210)/FAM 0d 1 1.jpg]

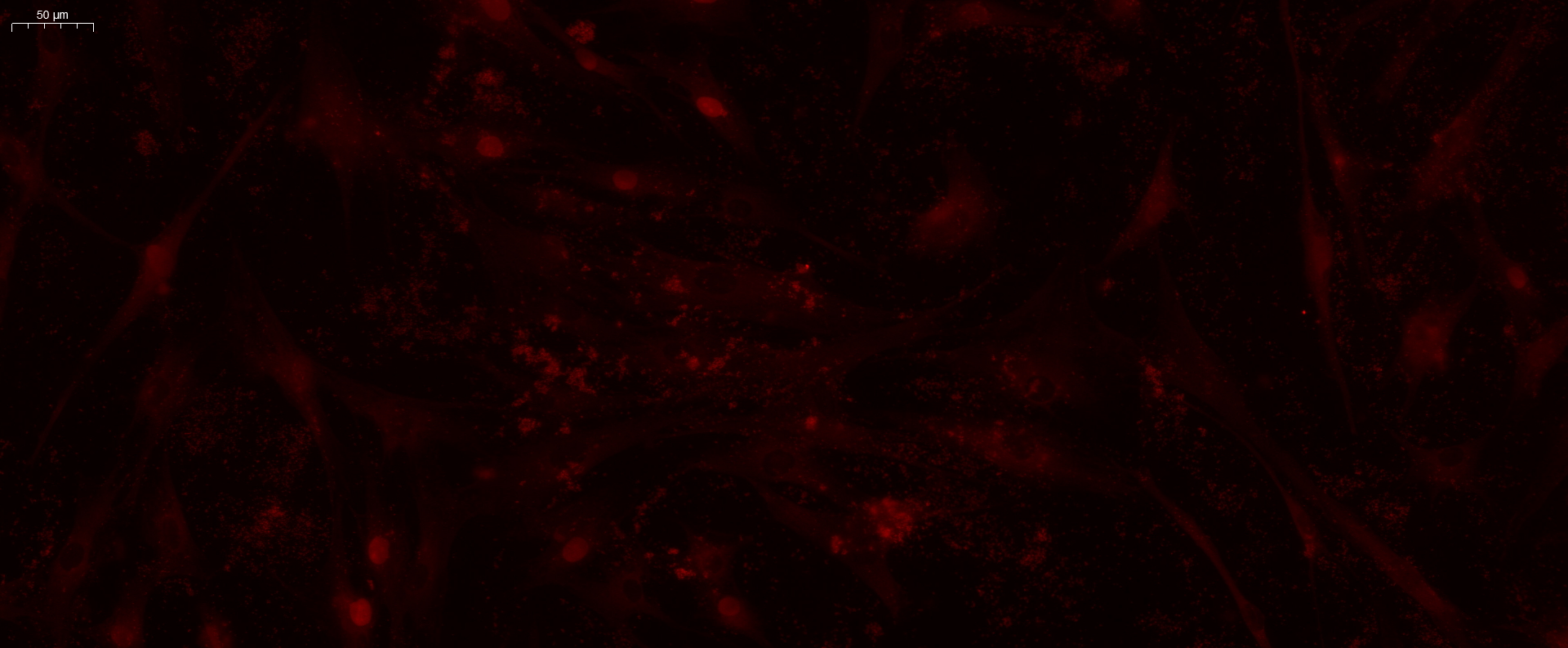

Supplement: Supplemental Information 5 [file peerj-10-13862-s005.zip › Supplement File 1(Figure 1C FAM210)/FAM 14d 1 2.jpg]

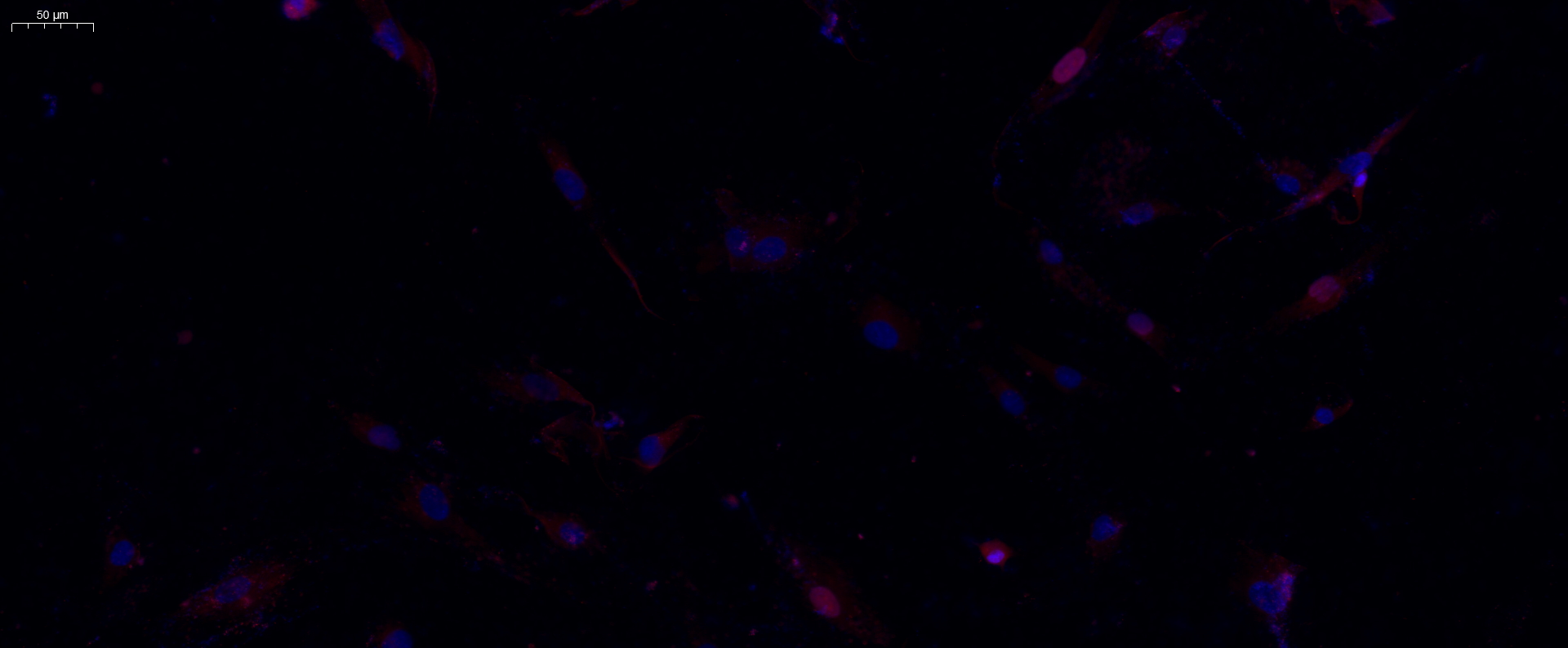

Supplement: Supplemental Information 5 [file peerj-10-13862-s005.zip › Supplement File 1(Figure 1C FAM210)/FAM 1d 1.jpg]

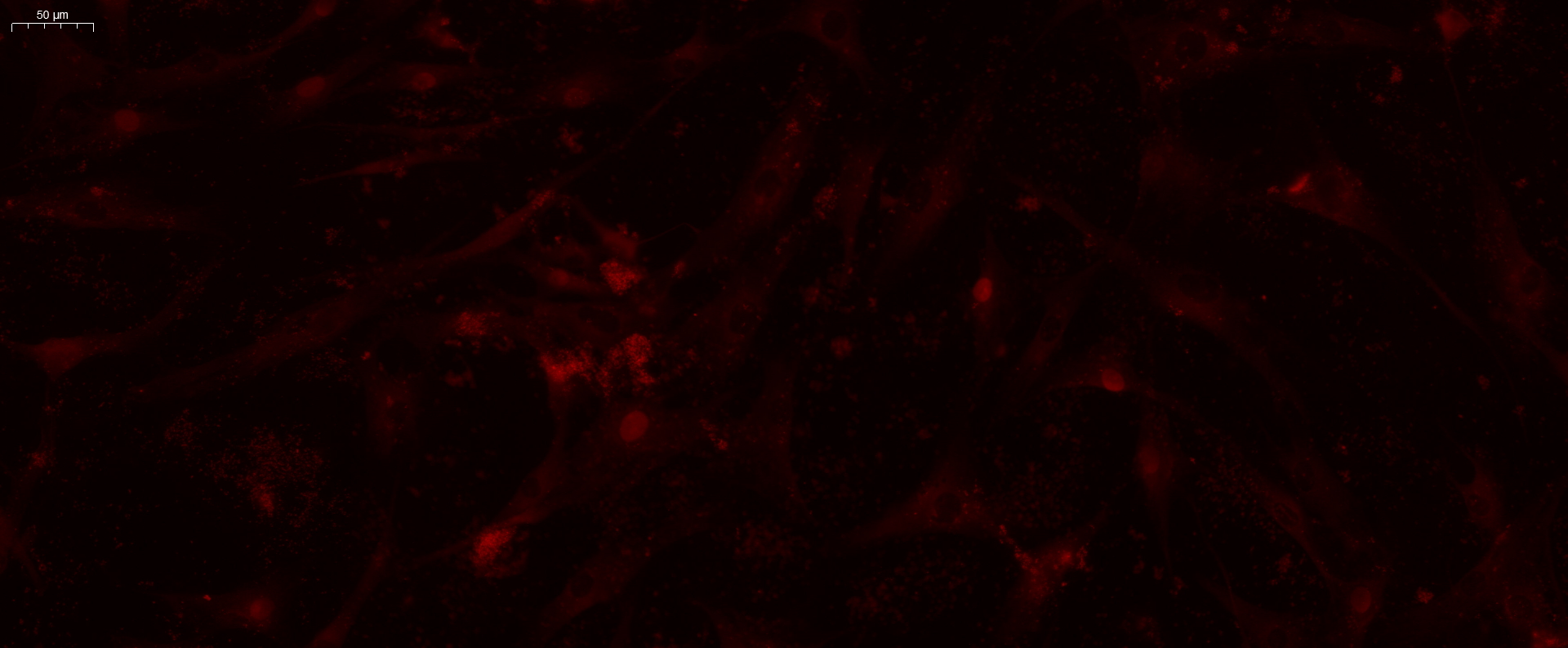

Supplement: Supplemental Information 5 [file peerj-10-13862-s005.zip › Supplement File 1(Figure 1C FAM210)/FAM 7d 2 2.jpg]

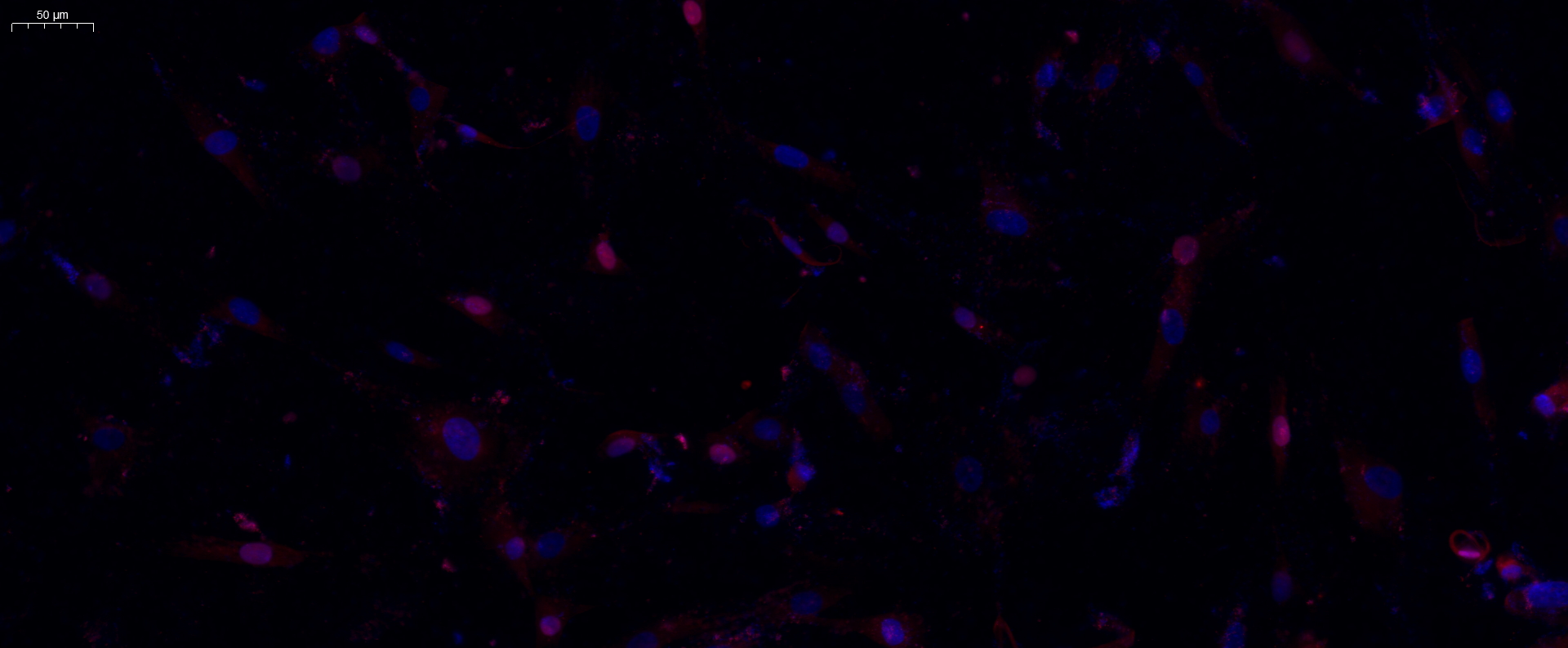

Supplement: Supplemental Information 5 [file peerj-10-13862-s005.zip › Supplement File 1(Figure 1C FAM210)/FAM 1d 2.jpg]

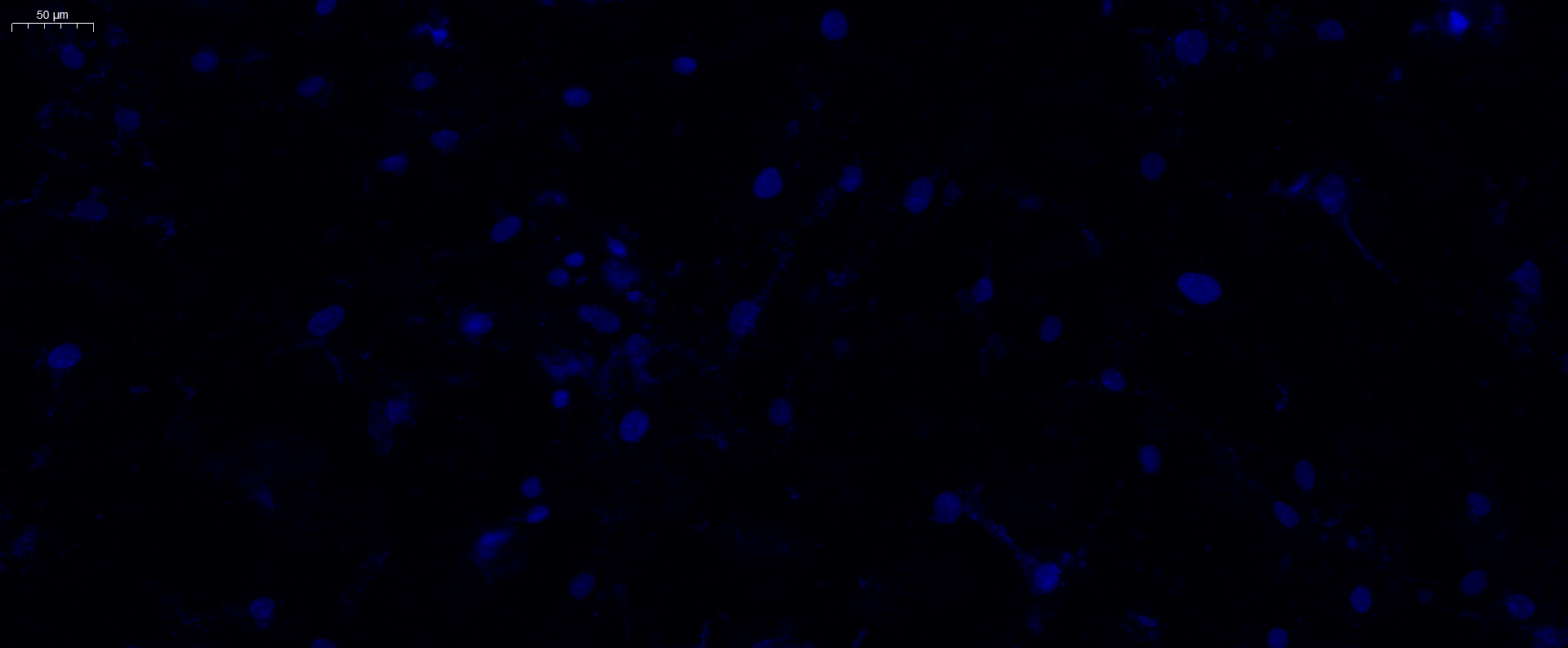

Supplement: Supplemental Information 5 [file peerj-10-13862-s005.zip › Supplement File 1(Figure 1C FAM210)/FAM 7d 2 1.jpg]

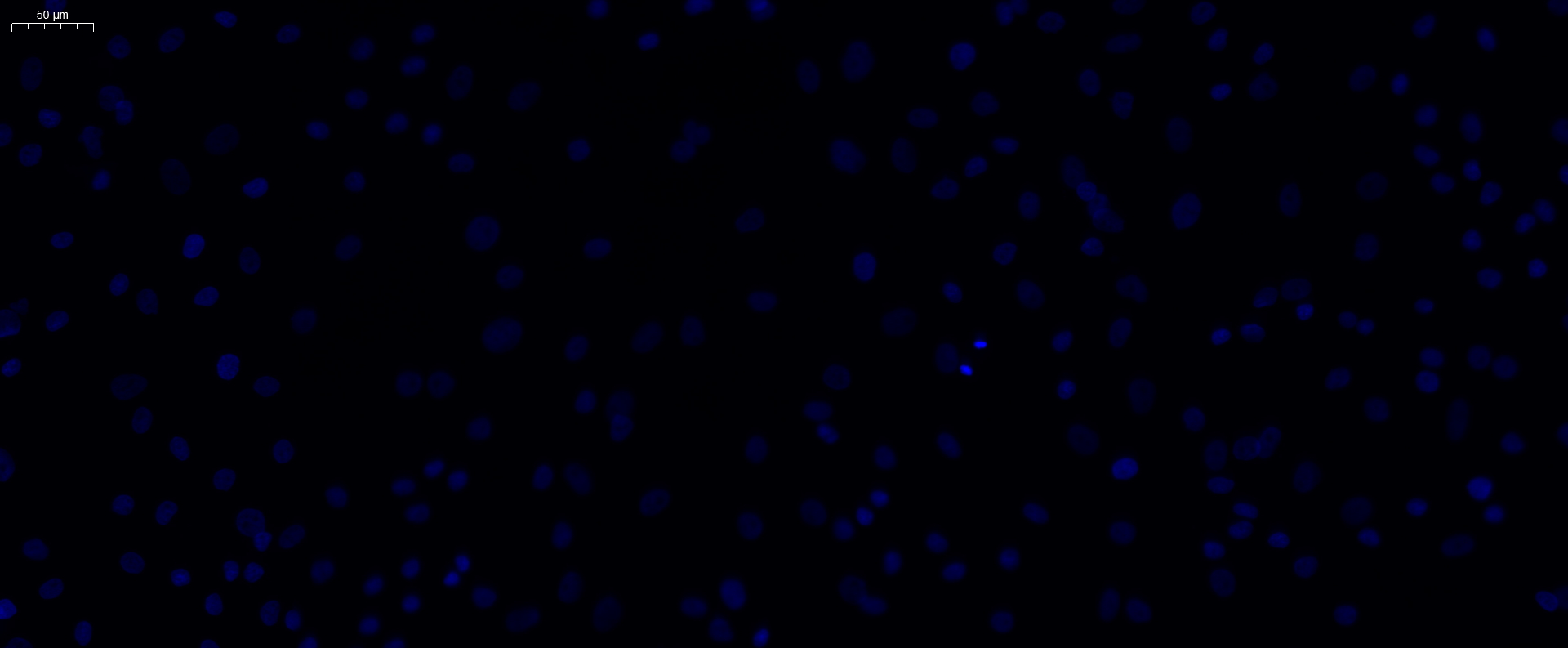

Supplement: Supplemental Information 6 [file peerj-10-13862-s006.zip › Supplement File 2(Figure 1C JMJD2B)/JMJD2B 3d 2 1.jpg]

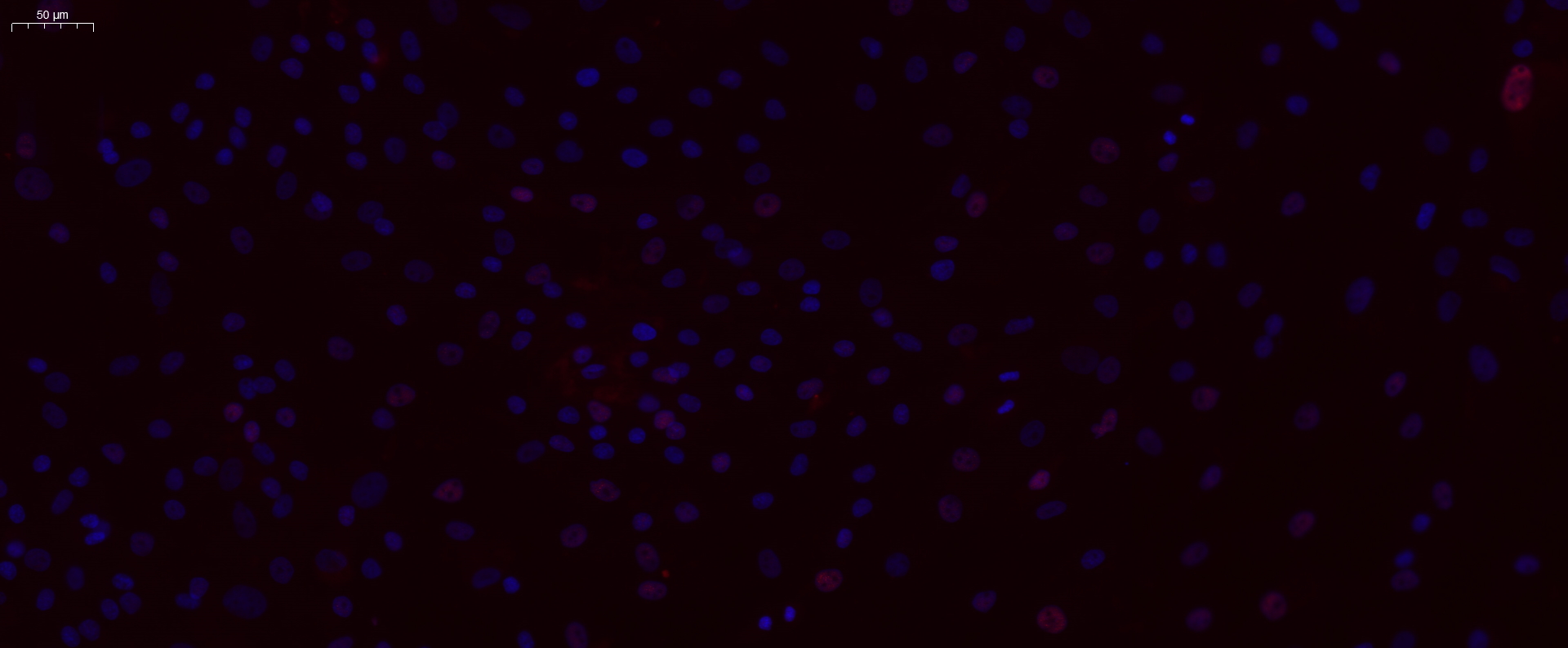

Supplement: Supplemental Information 6 [file peerj-10-13862-s006.zip › Supplement File 2(Figure 1C JMJD2B)/JMJD2B 3d 1.jpg]

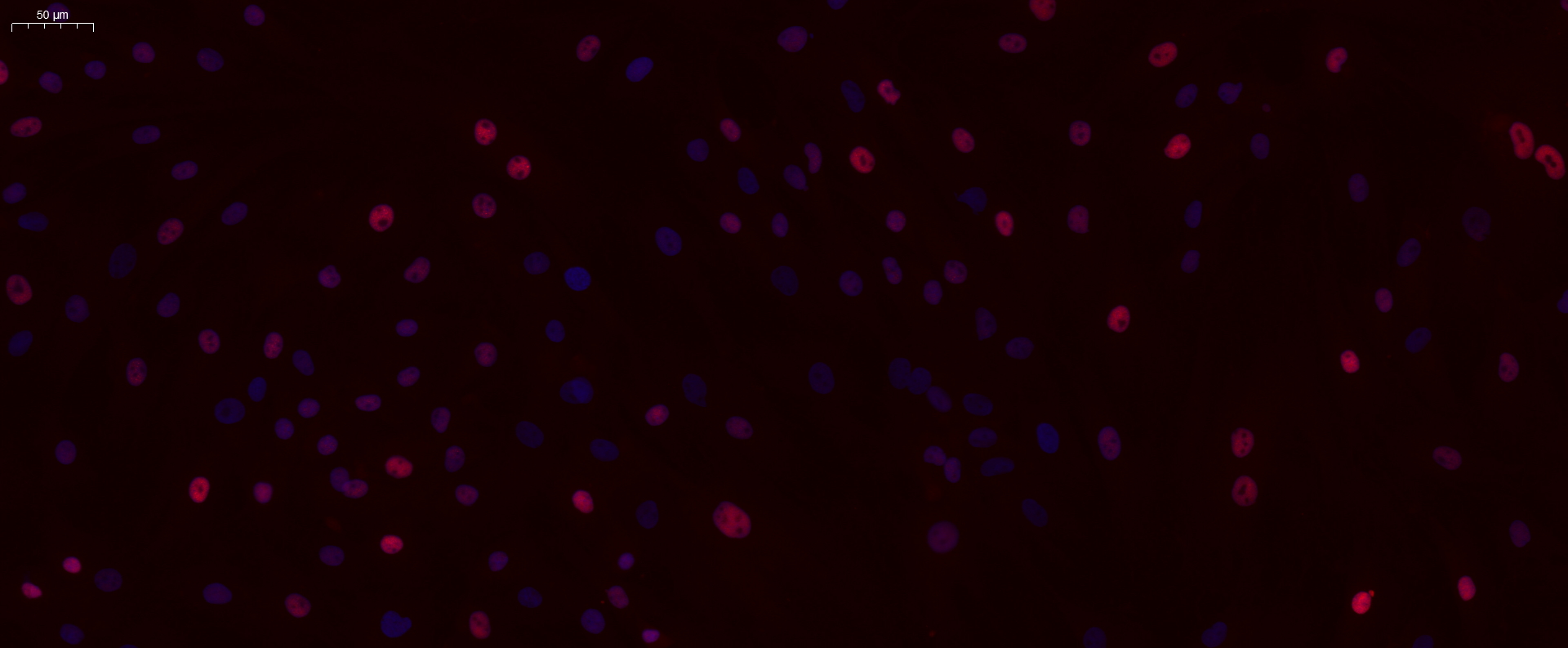

Supplement: Supplemental Information 6 [file peerj-10-13862-s006.zip › Supplement File 2(Figure 1C JMJD2B)/JMJD2B 14d 2.jpg]

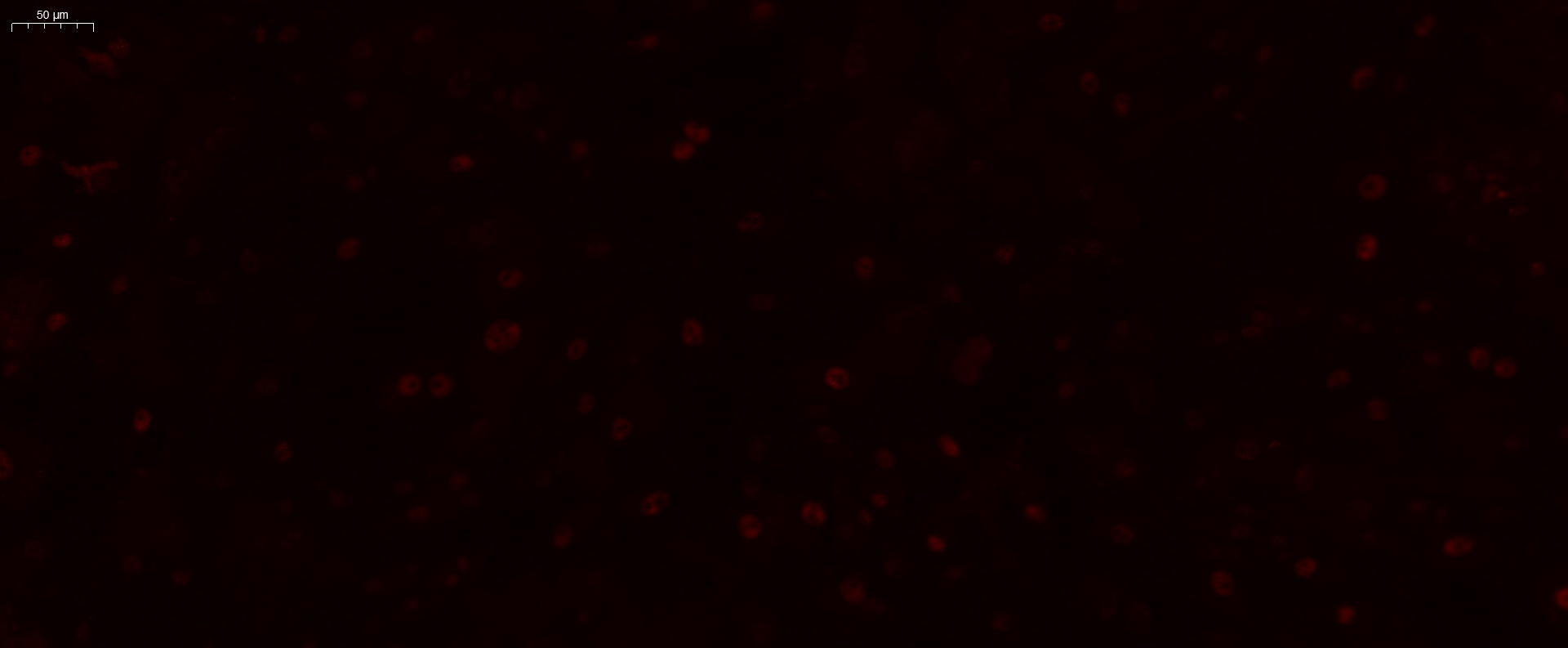

Supplement: Supplemental Information 6 [file peerj-10-13862-s006.zip › Supplement File 2(Figure 1C JMJD2B)/JMJD2B 3d 2 2.jpg]

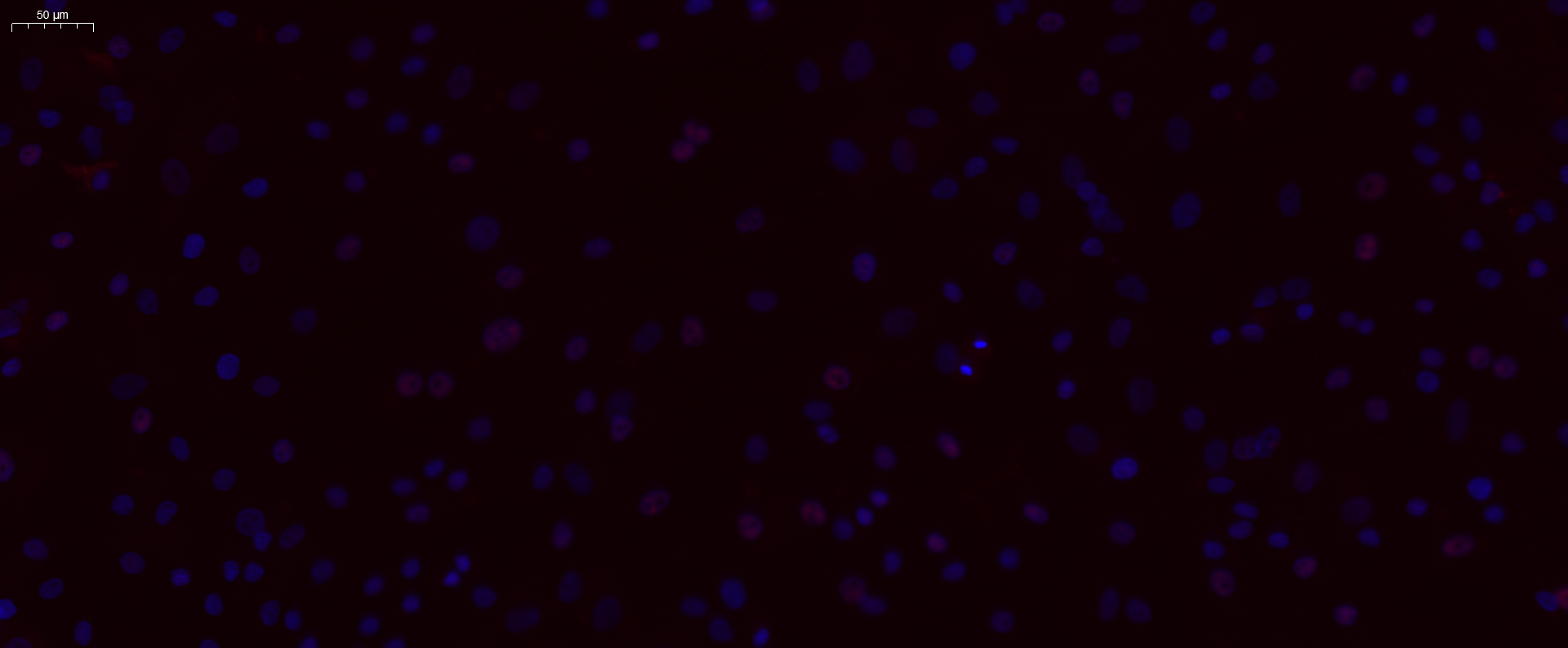

Supplement: Supplemental Information 6 [file peerj-10-13862-s006.zip › Supplement File 2(Figure 1C JMJD2B)/JMJD2B 3d 2.jpg]

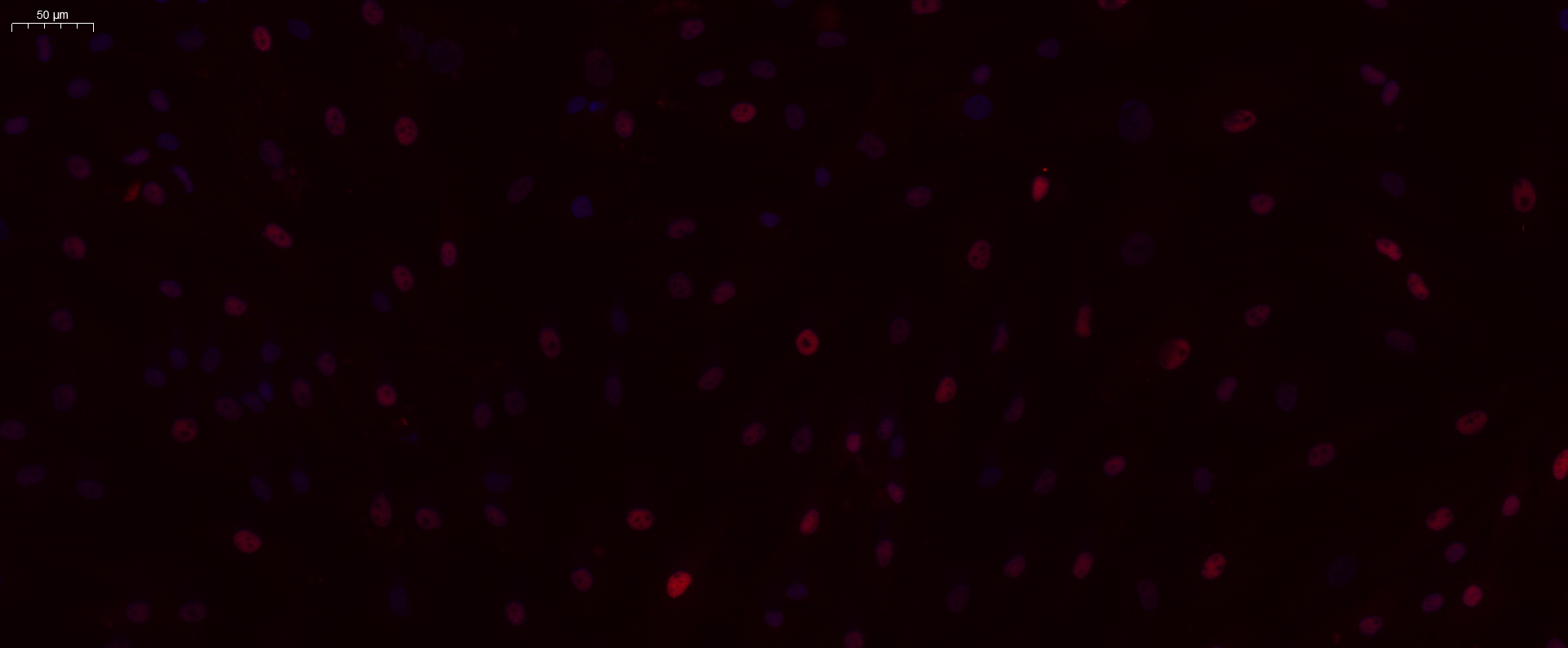

Supplement: Supplemental Information 6 [file peerj-10-13862-s006.zip › Supplement File 2(Figure 1C JMJD2B)/JMJD2B 14d 1.jpg]

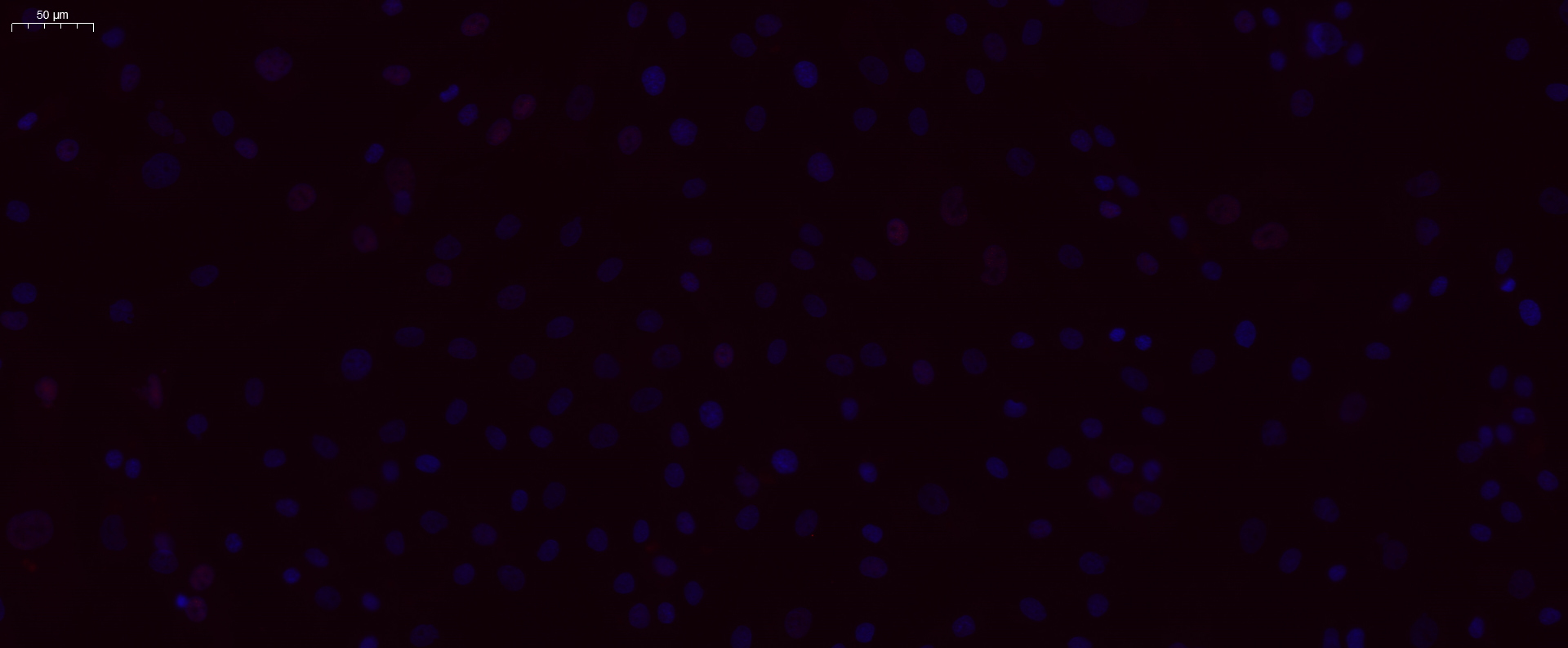

Supplement: Supplemental Information 6 [file peerj-10-13862-s006.zip › Supplement File 2(Figure 1C JMJD2B)/JMJD2B 1d 2.jpg]

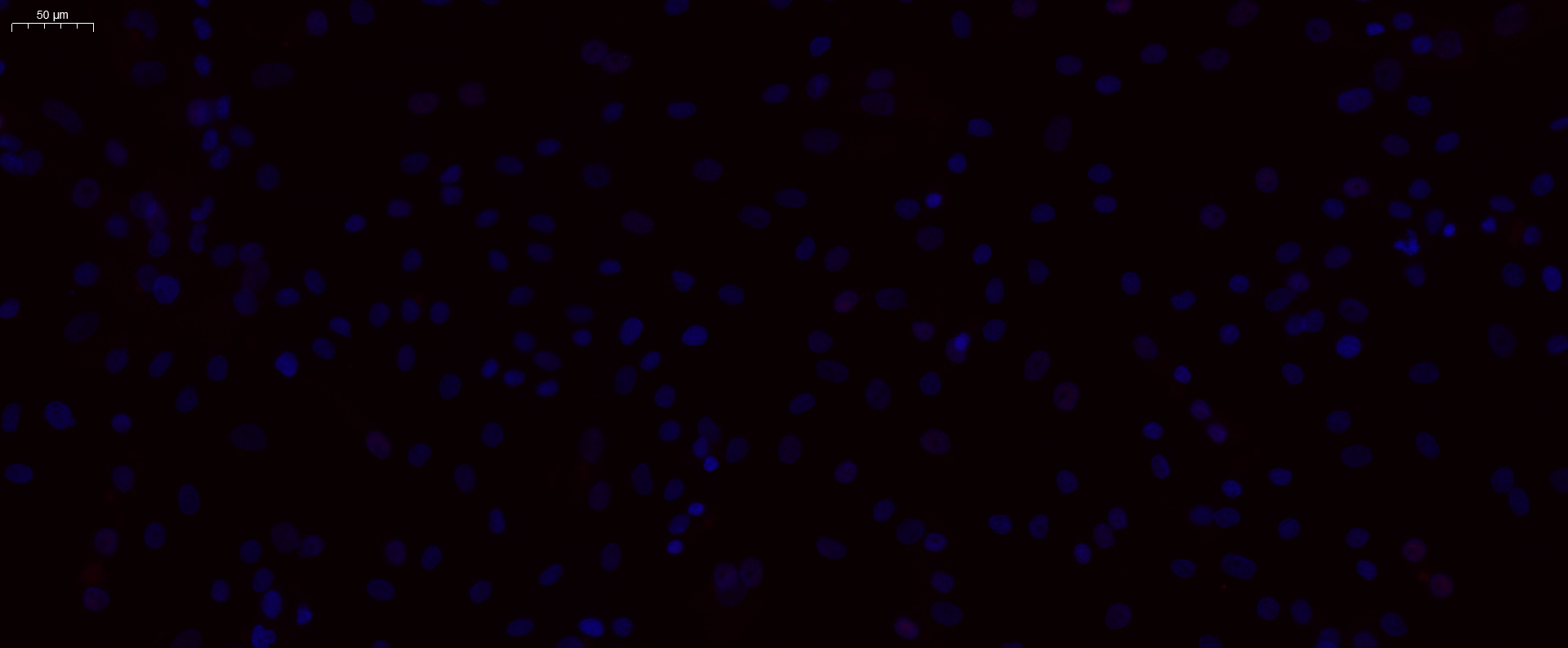

Supplement: Supplemental Information 6 [file peerj-10-13862-s006.zip › Supplement File 2(Figure 1C JMJD2B)/JMJD2B 1d 1.jpg]

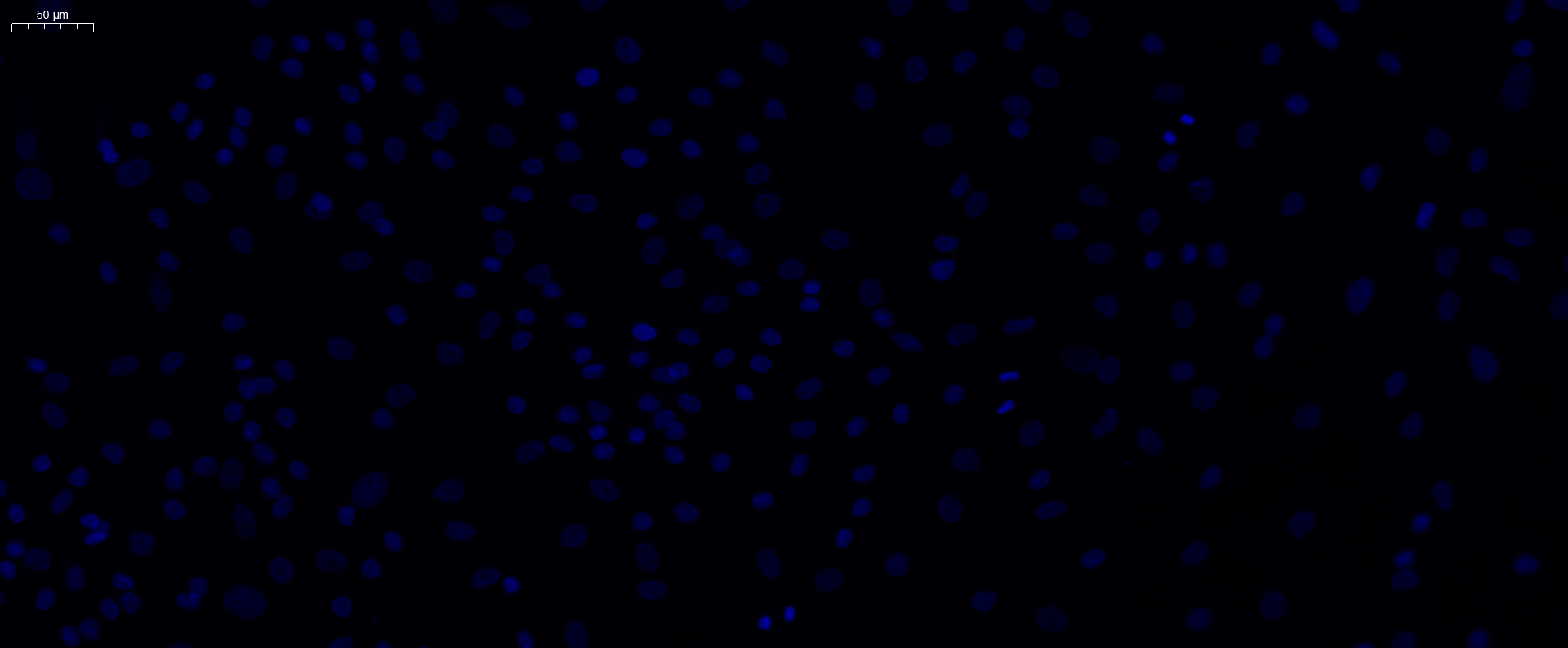

Supplement: Supplemental Information 6 [file peerj-10-13862-s006.zip › Supplement File 2(Figure 1C JMJD2B)/JMJD2B 3d 1 1.jpg]

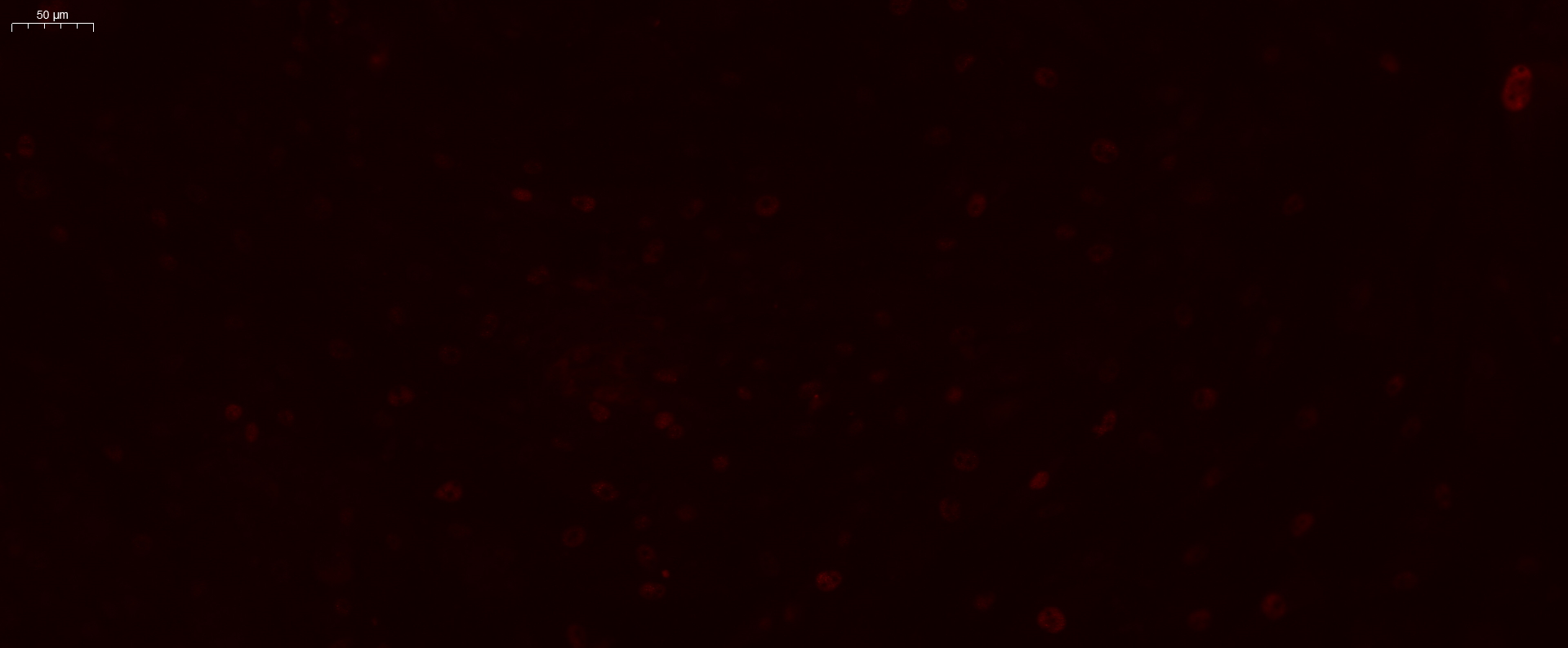

Supplement: Supplemental Information 6 [file peerj-10-13862-s006.zip › Supplement File 2(Figure 1C JMJD2B)/JMJD2B 3d 1 2.jpg]

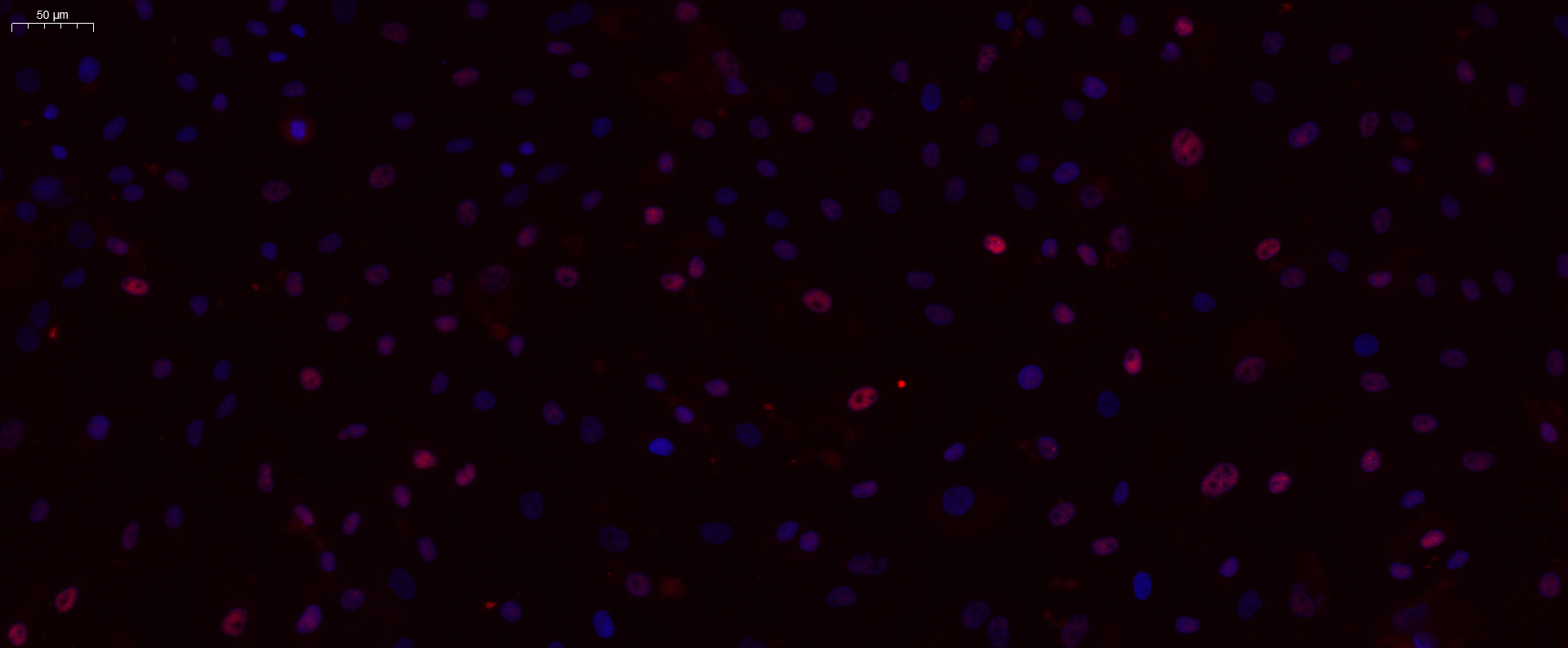

Supplement: Supplemental Information 6 [file peerj-10-13862-s006.zip › Supplement File 2(Figure 1C JMJD2B)/JMJD2B 7d 1.jpg]

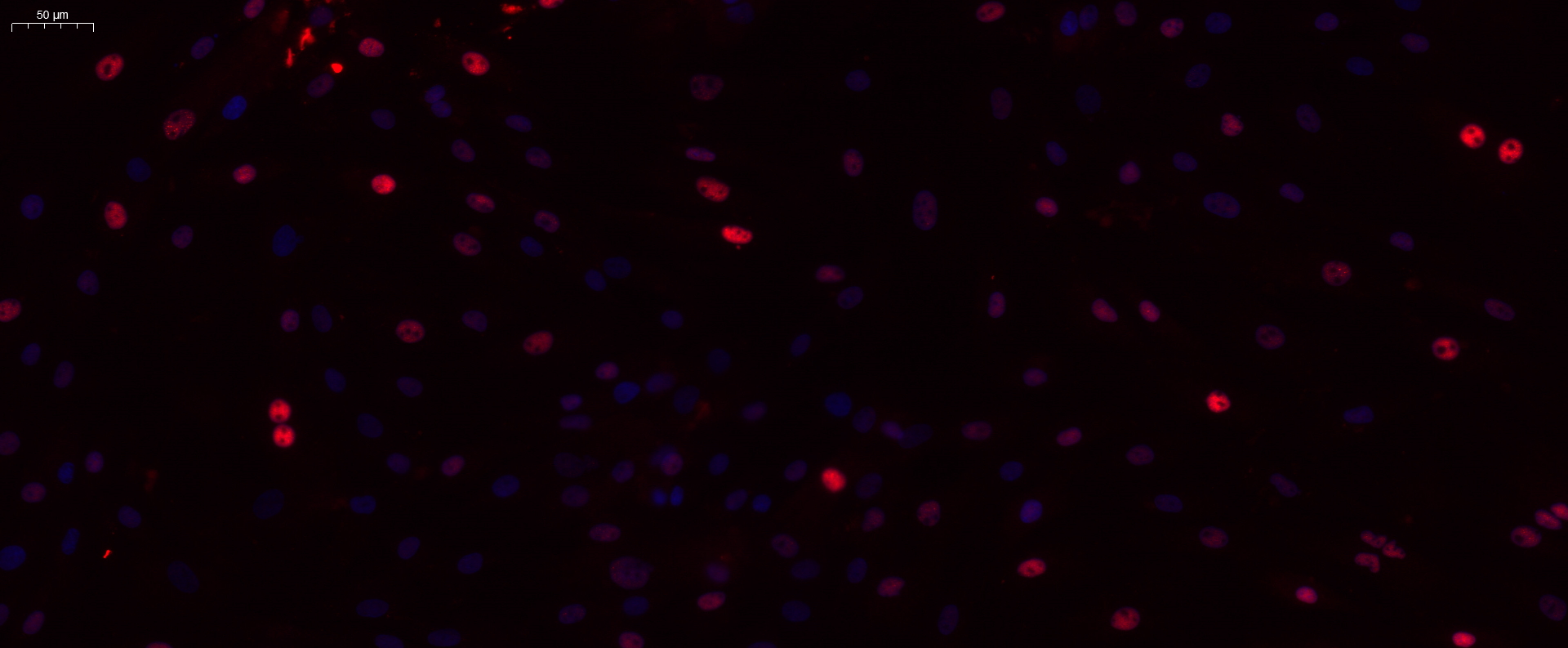

Supplement: Supplemental Information 6 [file peerj-10-13862-s006.zip › Supplement File 2(Figure 1C JMJD2B)/JMJD2B 7d 2.jpg]

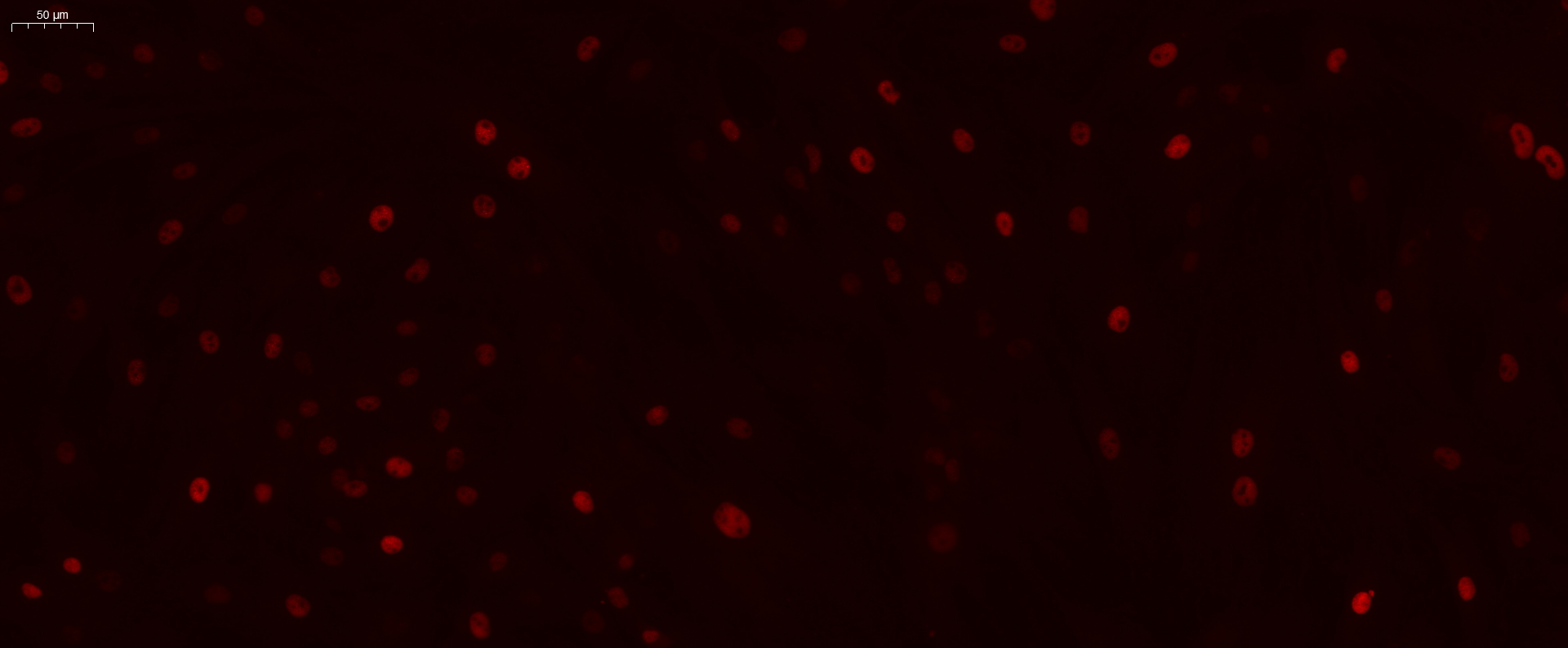

Supplement: Supplemental Information 6 [file peerj-10-13862-s006.zip › Supplement File 2(Figure 1C JMJD2B)/JMJD2B 14d 2 2.jpg]

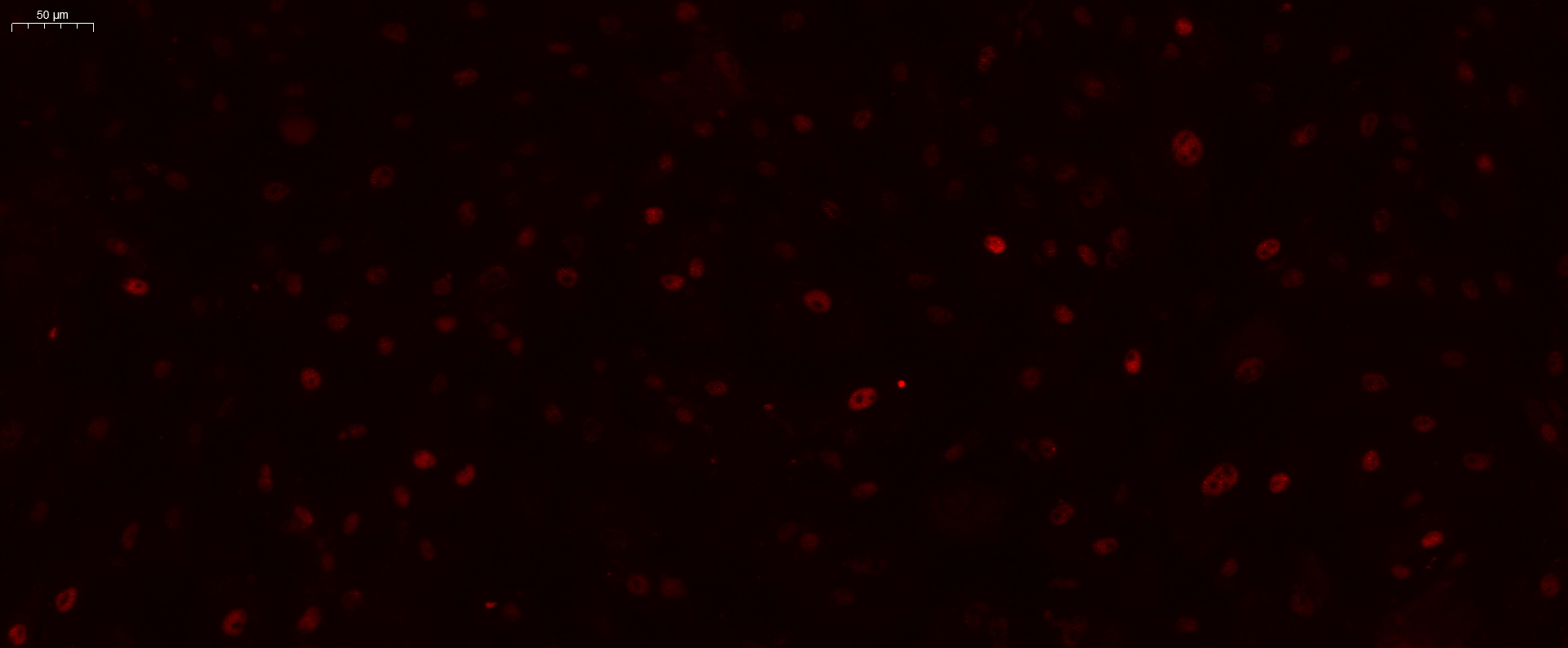

Supplement: Supplemental Information 6 [file peerj-10-13862-s006.zip › Supplement File 2(Figure 1C JMJD2B)/JMJD2B 7d 1 2.jpg]

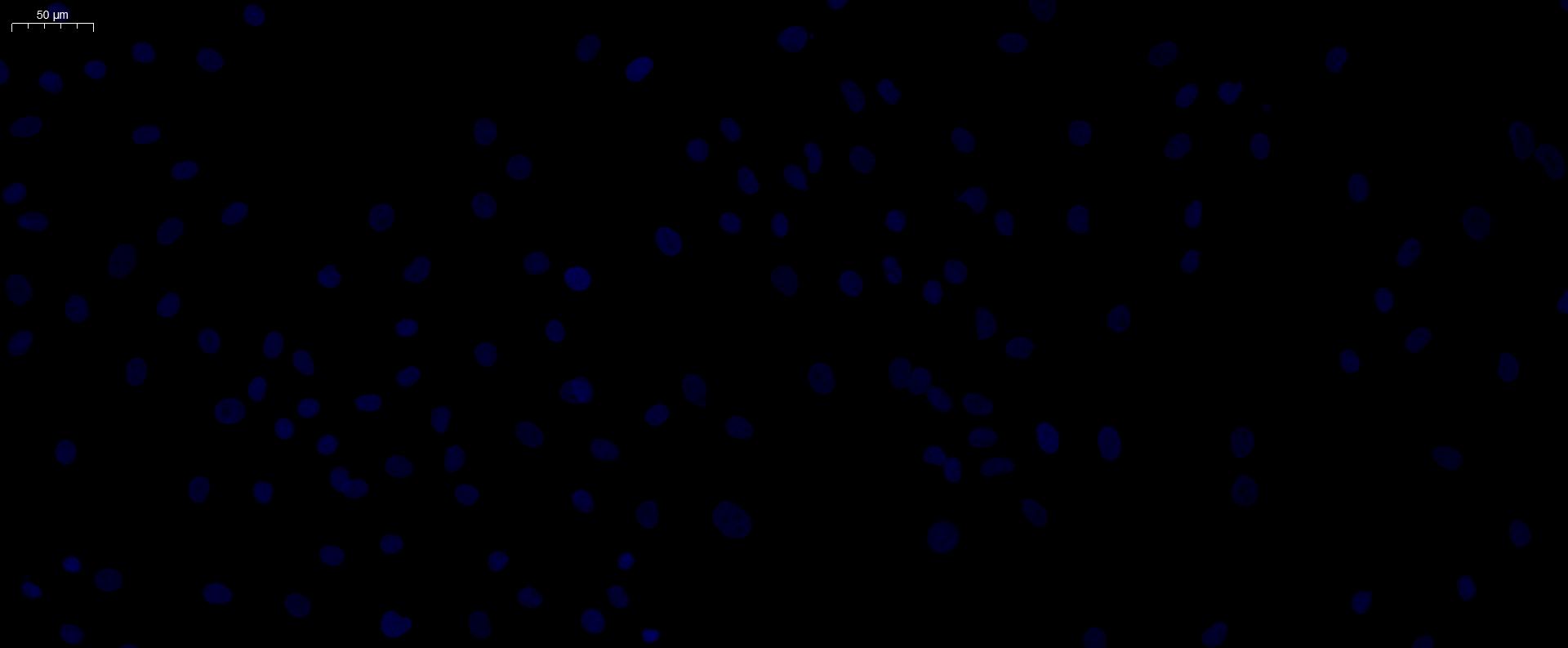

Supplement: Supplemental Information 6 [file peerj-10-13862-s006.zip › Supplement File 2(Figure 1C JMJD2B)/JMJD2B 14d 2 1.jpg]

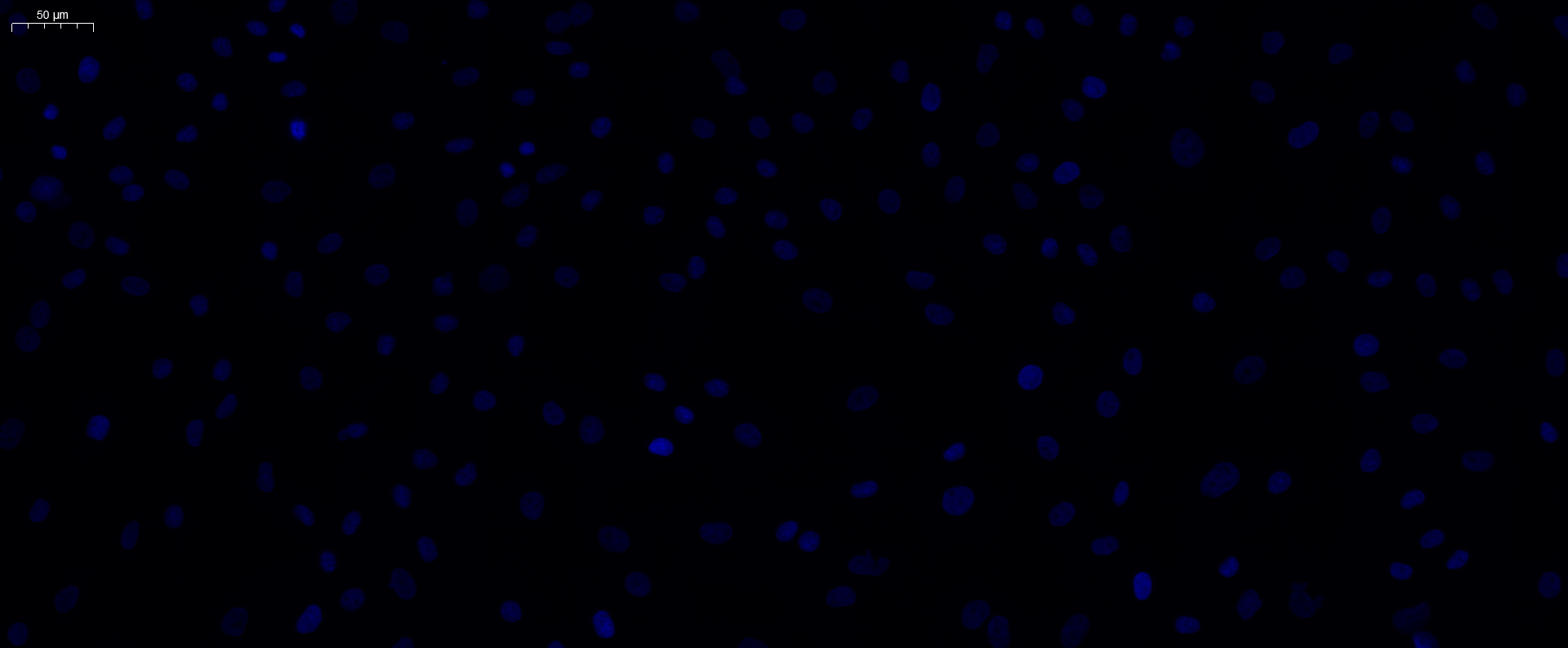

Supplement: Supplemental Information 6 [file peerj-10-13862-s006.zip › Supplement File 2(Figure 1C JMJD2B)/JMJD2B 7d 1 1.jpg]

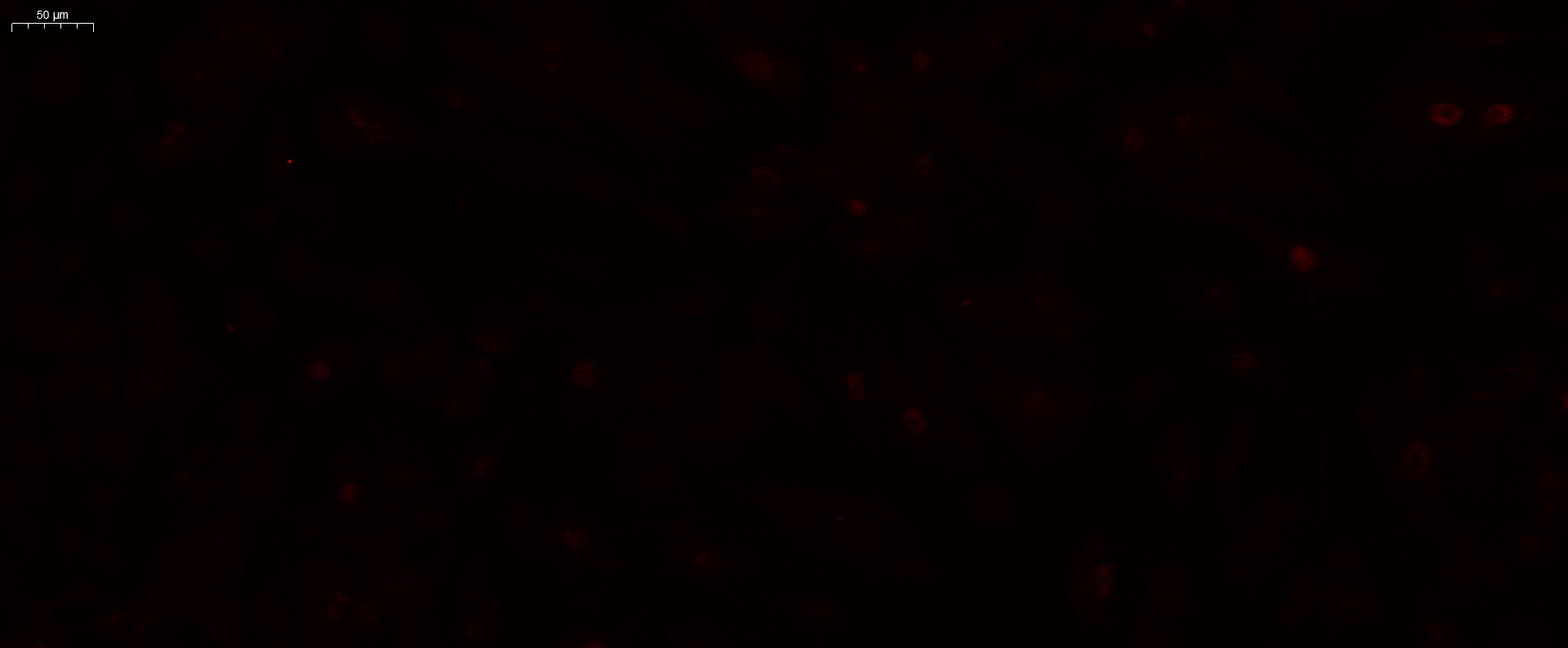

Supplement: Supplemental Information 6 [file peerj-10-13862-s006.zip › Supplement File 2(Figure 1C JMJD2B)/JMJD2B 0d 2 2.jpg]

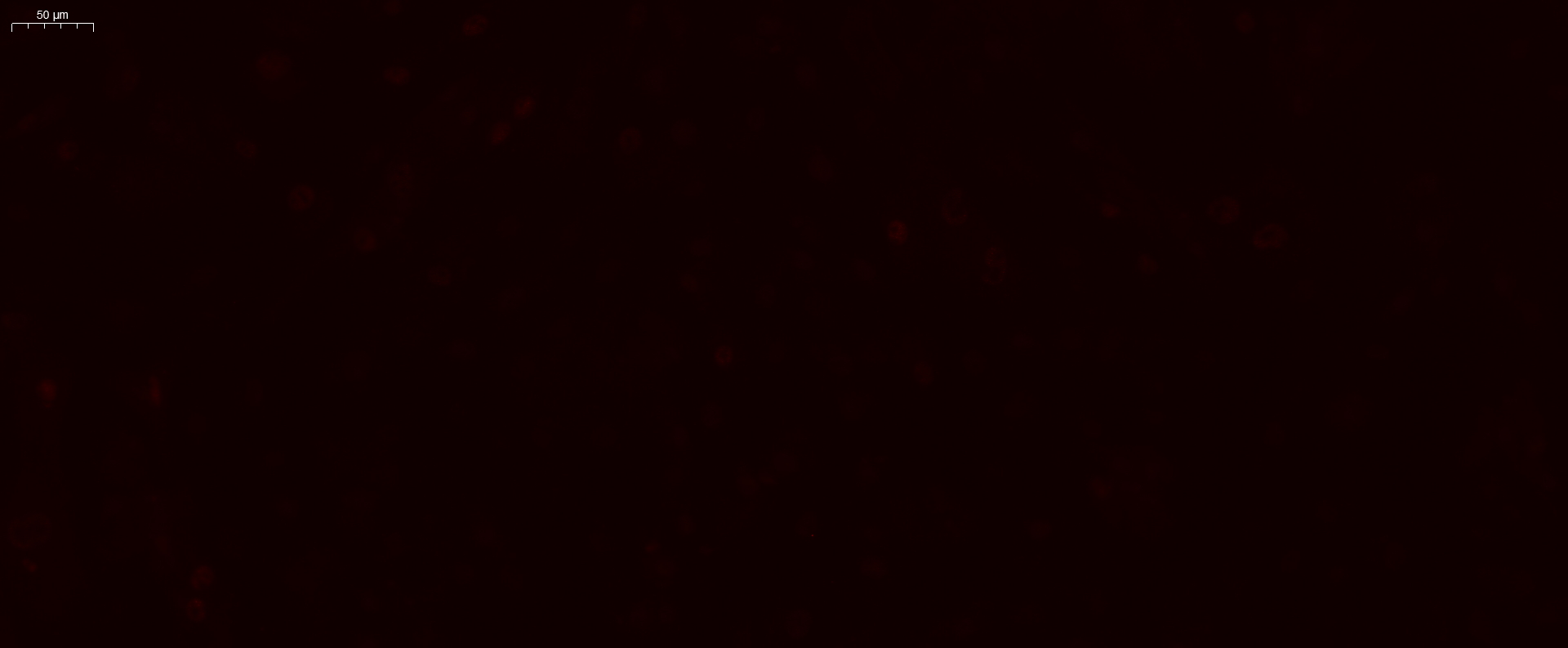

Supplement: Supplemental Information 6 [file peerj-10-13862-s006.zip › Supplement File 2(Figure 1C JMJD2B)/JMJD2B 1d 2 2.jpg]

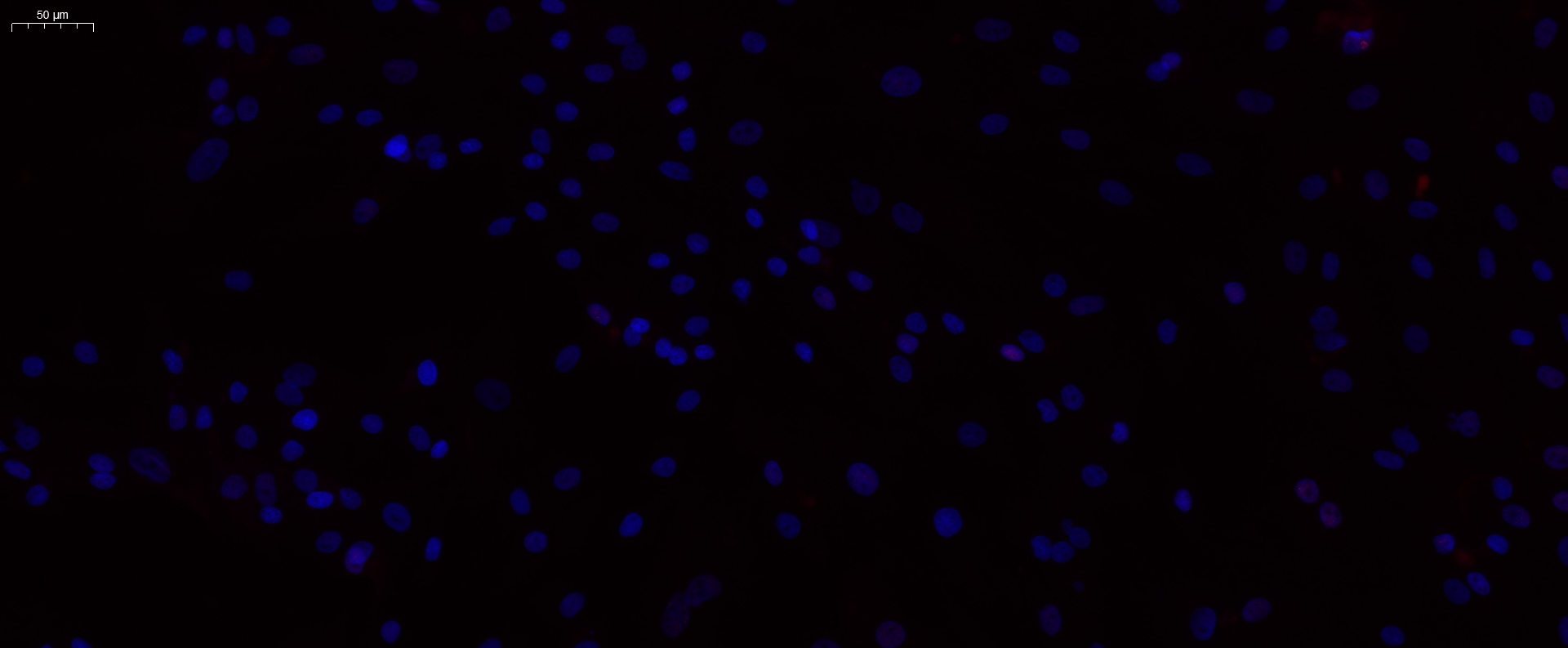

Supplement: Supplemental Information 6 [file peerj-10-13862-s006.zip › Supplement File 2(Figure 1C JMJD2B)/JMJD2B 0d 1.jpg]

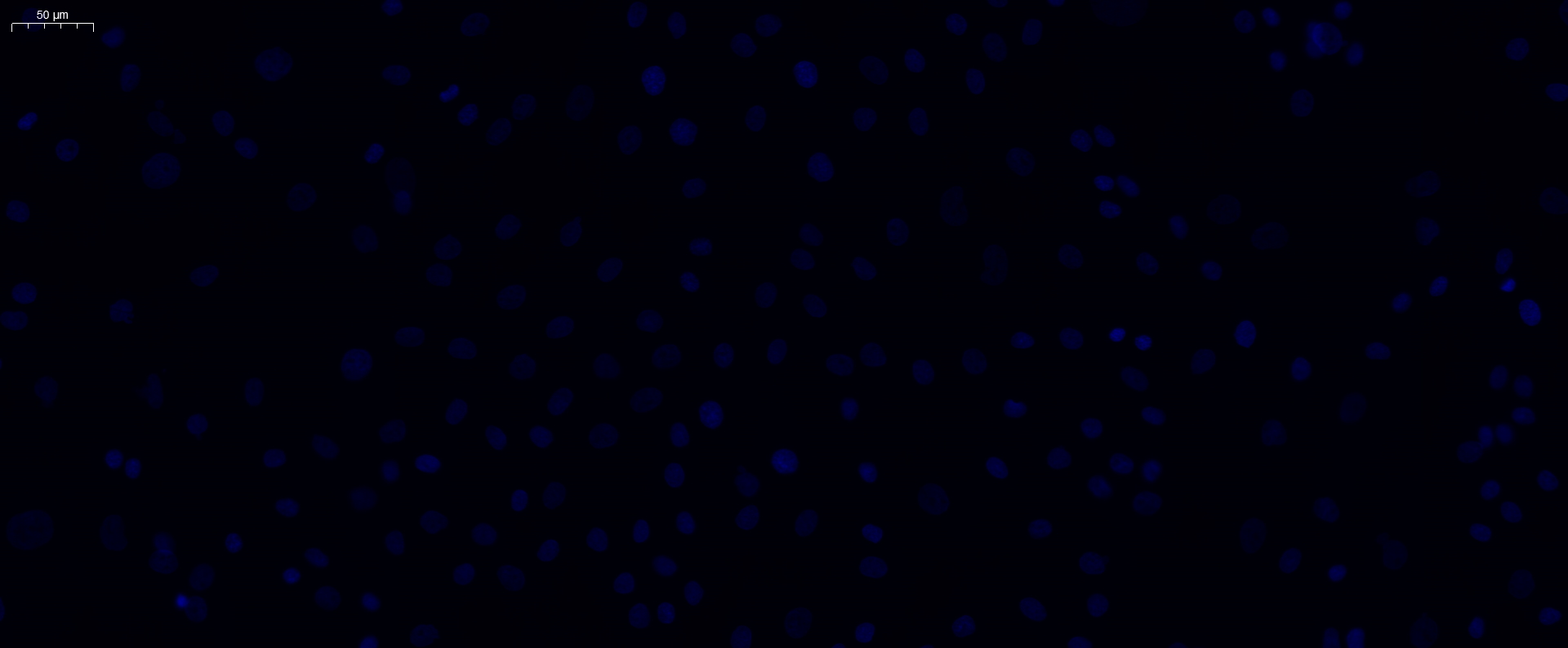

Supplement: Supplemental Information 6 [file peerj-10-13862-s006.zip › Supplement File 2(Figure 1C JMJD2B)/JMJD2B 1d 2 1.jpg]

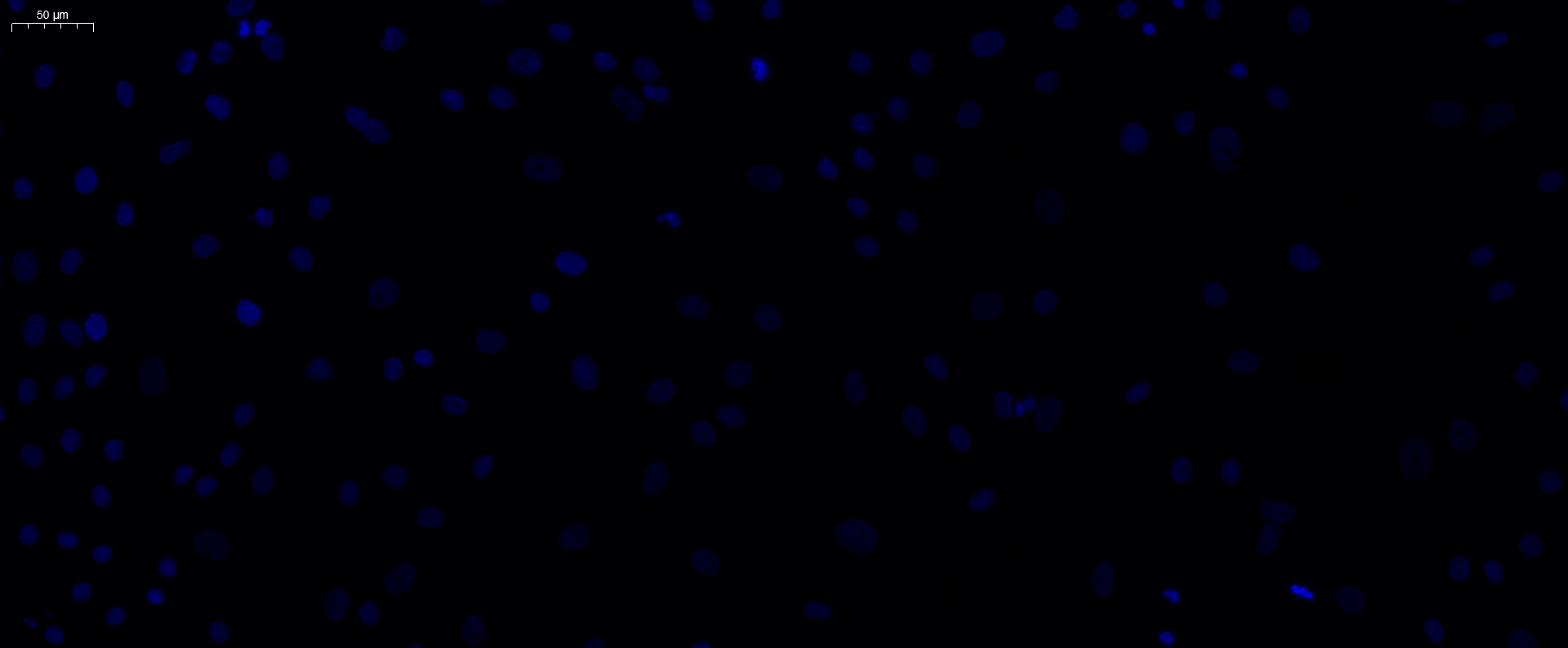

Supplement: Supplemental Information 6 [file peerj-10-13862-s006.zip › Supplement File 2(Figure 1C JMJD2B)/JMJD2B 0d 2 1.jpg]

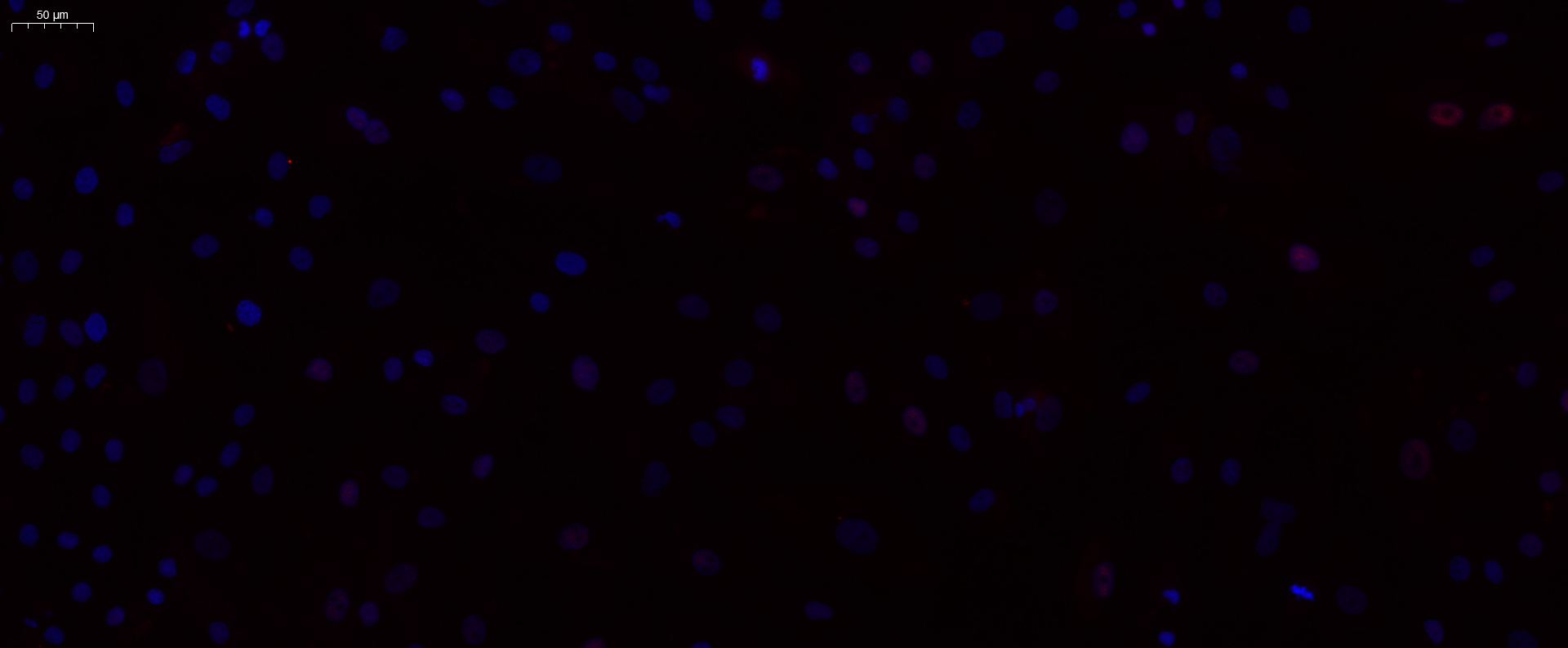

Supplement: Supplemental Information 6 [file peerj-10-13862-s006.zip › Supplement File 2(Figure 1C JMJD2B)/JMJD2B 0d 2.jpg]

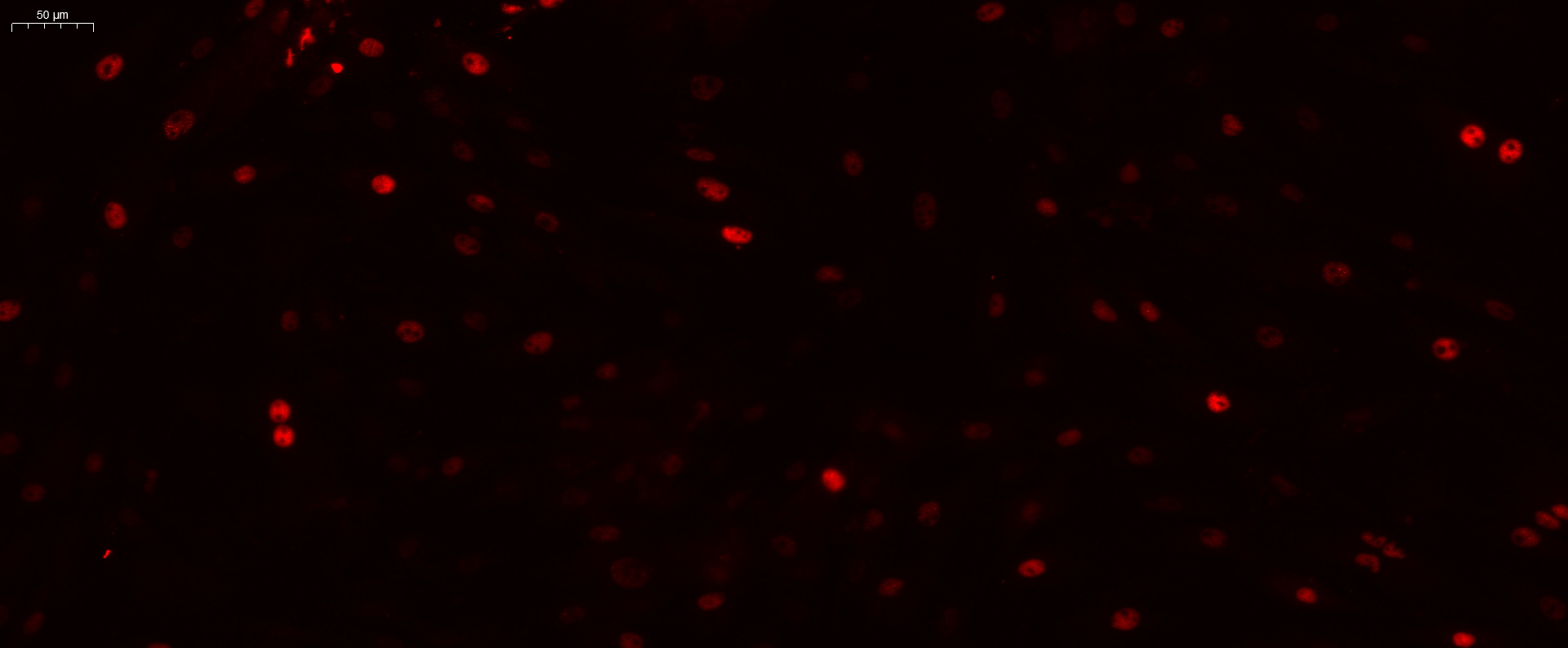

Supplement: Supplemental Information 6 [file peerj-10-13862-s006.zip › Supplement File 2(Figure 1C JMJD2B)/JMJD2B 7d 2 2.jpg]

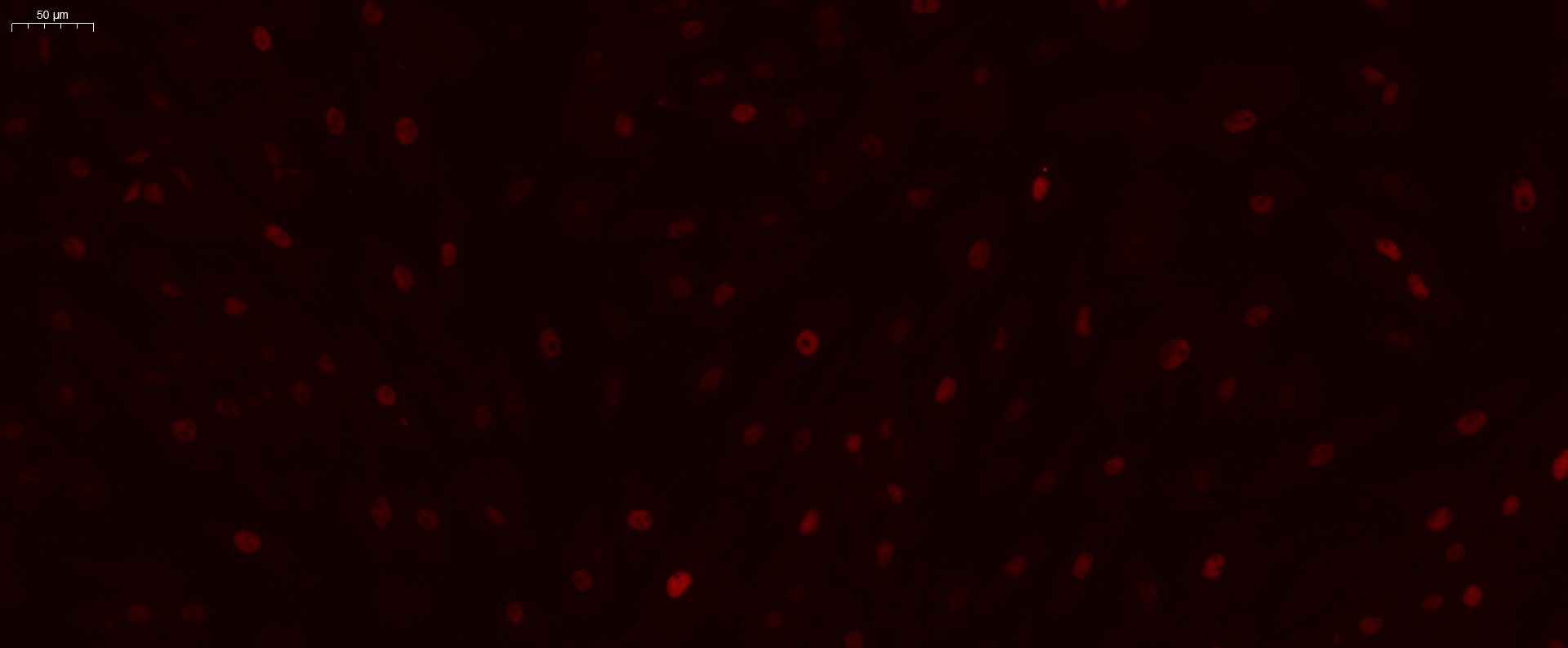

Supplement: Supplemental Information 6 [file peerj-10-13862-s006.zip › Supplement File 2(Figure 1C JMJD2B)/JMJD2B 14d 1 2.jpg]

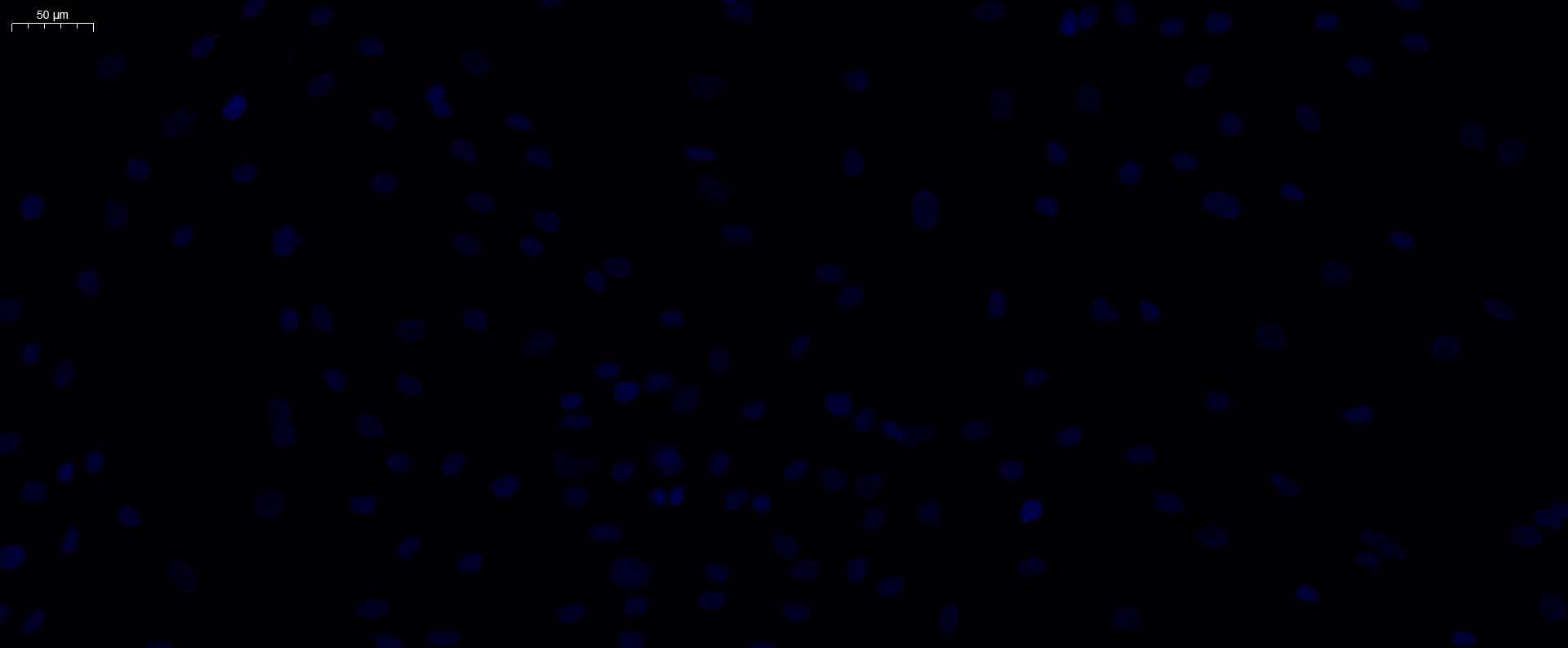

Supplement: Supplemental Information 6 [file peerj-10-13862-s006.zip › Supplement File 2(Figure 1C JMJD2B)/JMJD2B 7d 2 1.jpg]

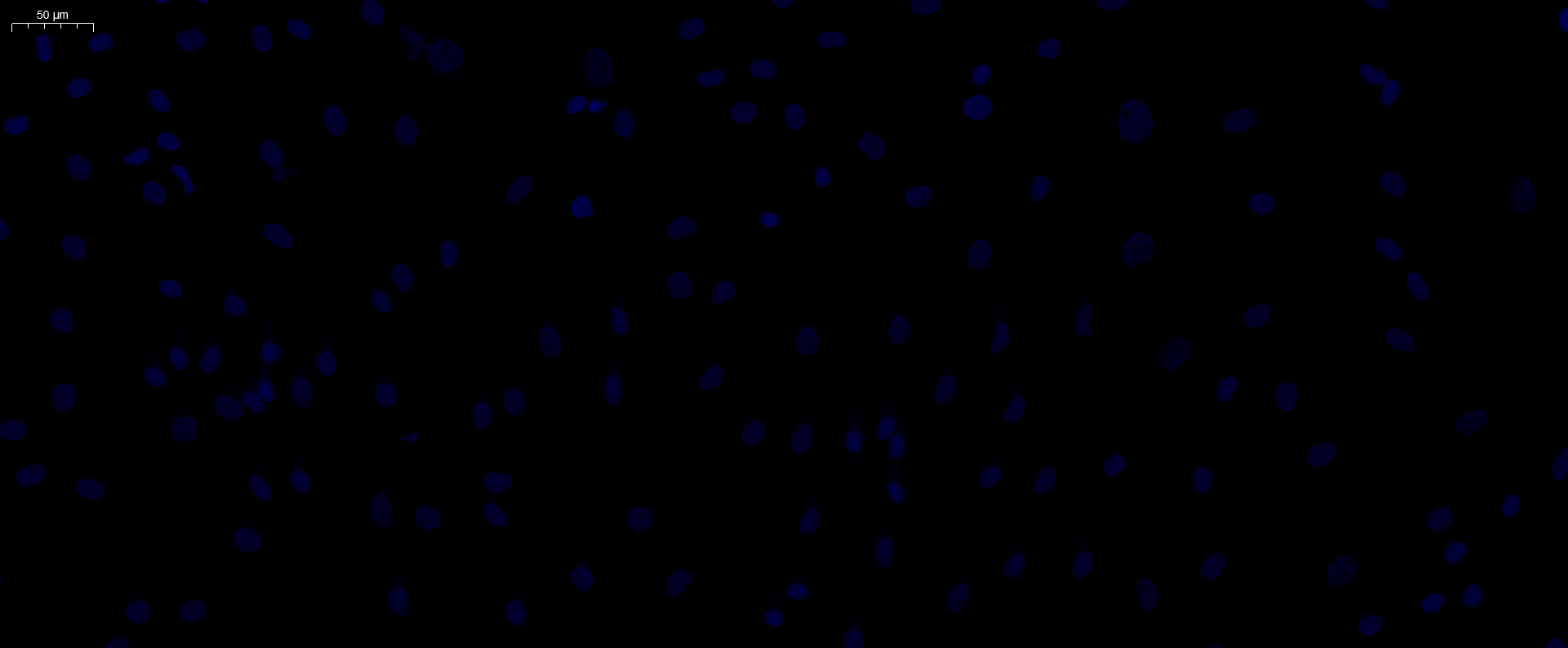

Supplement: Supplemental Information 6 [file peerj-10-13862-s006.zip › Supplement File 2(Figure 1C JMJD2B)/JMJD2B 14d 1 1.jpg]

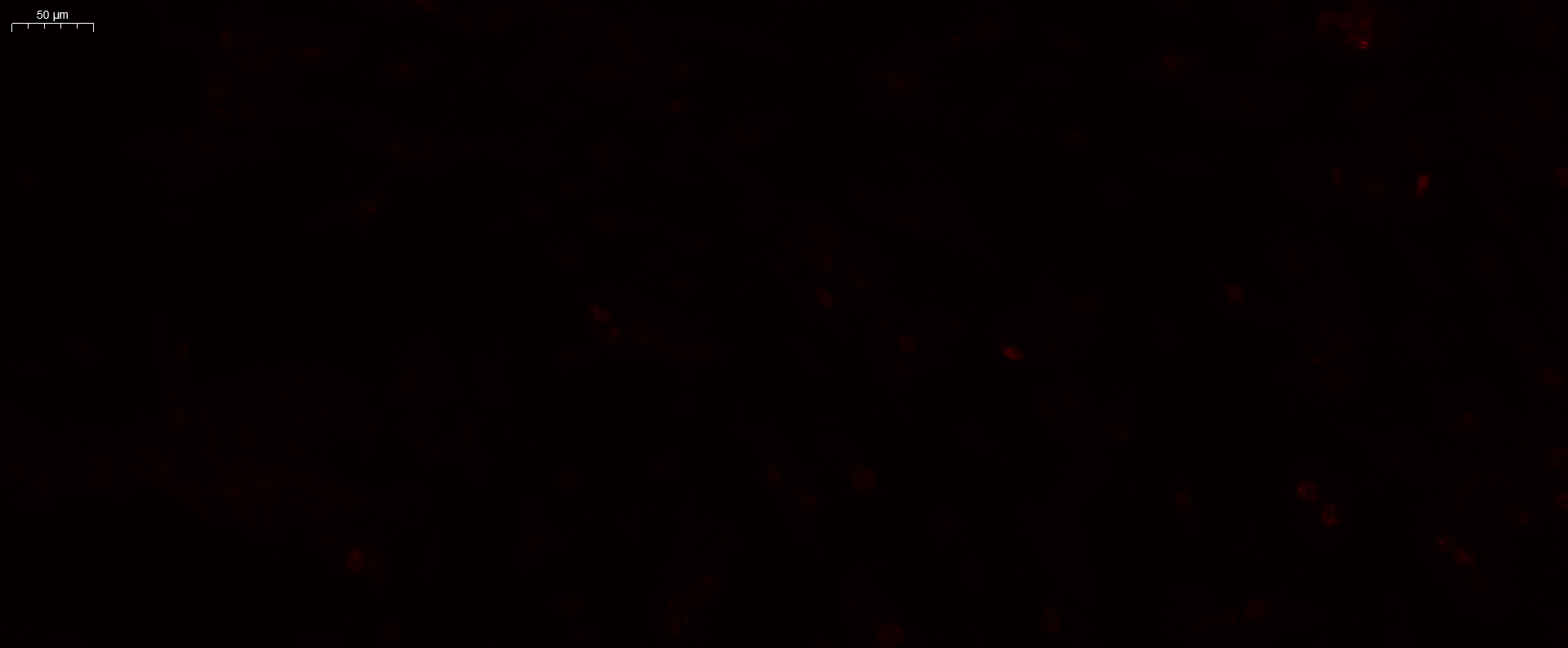

Supplement: Supplemental Information 6 [file peerj-10-13862-s006.zip › Supplement File 2(Figure 1C JMJD2B)/JMJD2B 0d 1 2.jpg]

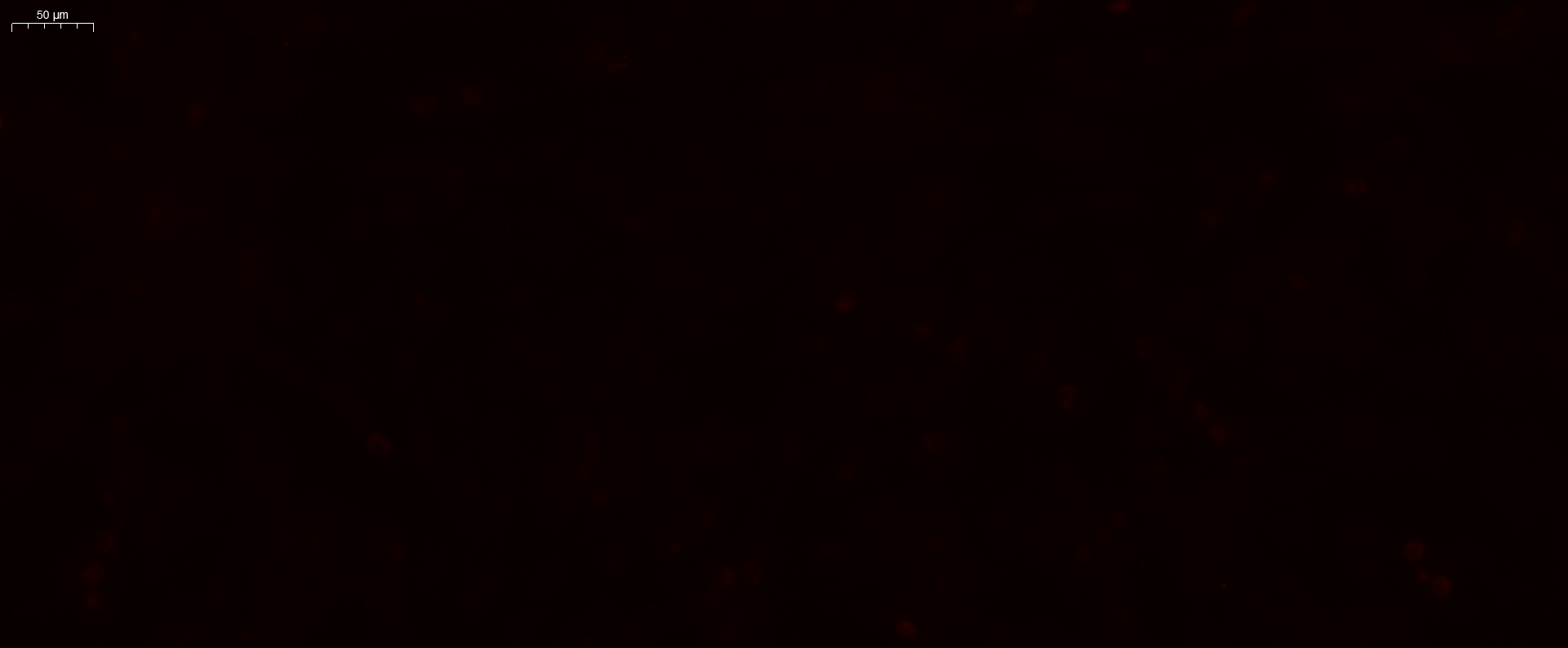

Supplement: Supplemental Information 6 [file peerj-10-13862-s006.zip › Supplement File 2(Figure 1C JMJD2B)/JMJD2B 1d 1 2.jpg]

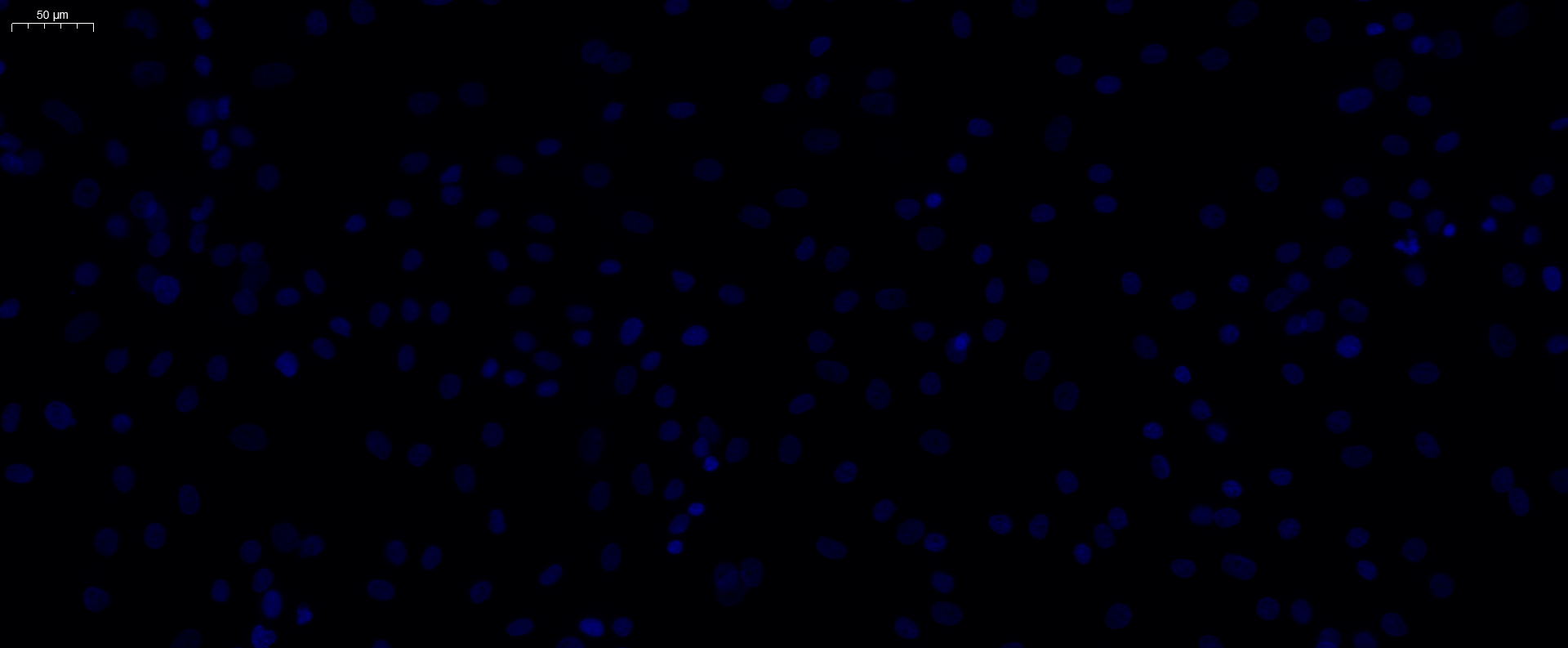

Supplement: Supplemental Information 6 [file peerj-10-13862-s006.zip › Supplement File 2(Figure 1C JMJD2B)/JMJD2B 1d 1 1.jpg]

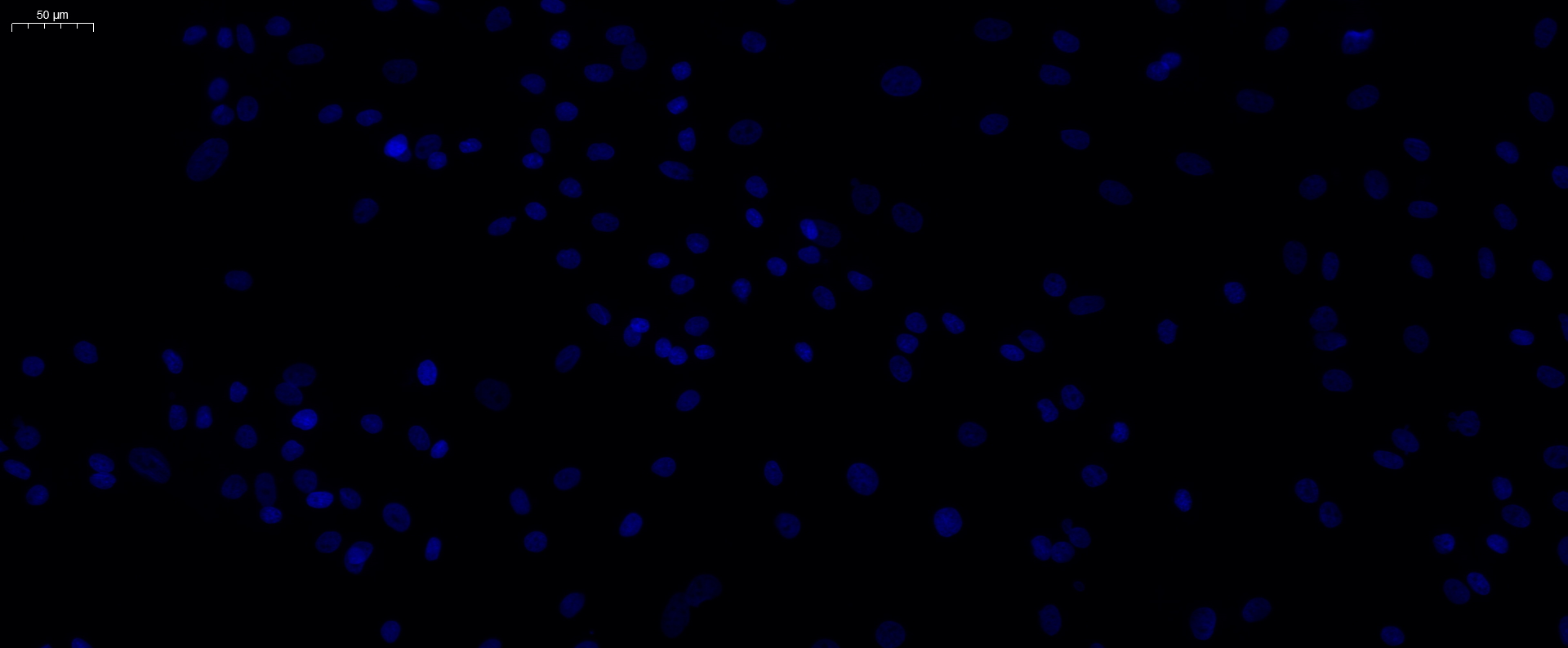

Supplement: Supplemental Information 6 [file peerj-10-13862-s006.zip › Supplement File 2(Figure 1C JMJD2B)/JMJD2B 0d 1 1.jpg]

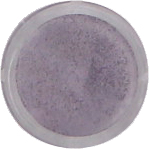

Supplement: Supplemental Information 9 [file peerj-10-13862-s009.zip › Supplement File 5(Figure 2C Left Panel)/D3 LV5-JMJD2B.jpg]

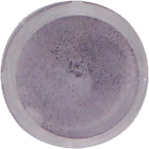

Supplement: Supplemental Information 9 [file peerj-10-13862-s009.zip › Supplement File 5(Figure 2C Left Panel)/D3 Scr.jpg]

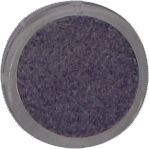

Supplement: Supplemental Information 9 [file peerj-10-13862-s009.zip › Supplement File 5(Figure 2C Left Panel)/D7 LV5-JMJD2B.jpg]

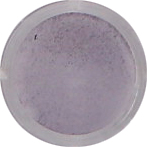

Supplement: Supplemental Information 9 [file peerj-10-13862-s009.zip › Supplement File 5(Figure 2C Left Panel)/D1 Scr.jpg]

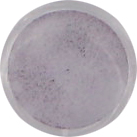

Supplement: Supplemental Information 9 [file peerj-10-13862-s009.zip › Supplement File 5(Figure 2C Left Panel)/D1 shJMJD2B.jpg]

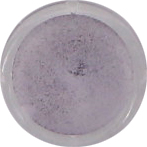

Supplement: Supplemental Information 9 [file peerj-10-13862-s009.zip › Supplement File 5(Figure 2C Left Panel)/D1 LV5-JMJD2B.jpg]

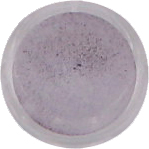

Supplement: Supplemental Information 9 [file peerj-10-13862-s009.zip › Supplement File 5(Figure 2C Left Panel)/D3 shJMJD2B.jpg]

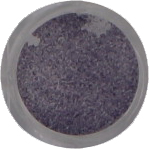

Supplement: Supplemental Information 9 [file peerj-10-13862-s009.zip › Supplement File 5(Figure 2C Left Panel)/D14 NC.jpg]

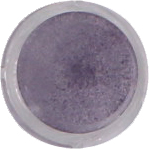

Supplement: Supplemental Information 9 [file peerj-10-13862-s009.zip › Supplement File 5(Figure 2C Left Panel)/D7 NC.jpg]

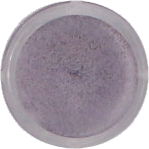

Supplement: Supplemental Information 9 [file peerj-10-13862-s009.zip › Supplement File 5(Figure 2C Left Panel)/D7 shMJD2B.jpg]

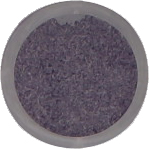

Supplement: Supplemental Information 9 [file peerj-10-13862-s009.zip › Supplement File 5(Figure 2C Left Panel)/D14 Scr.jpg]

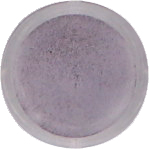

Supplement: Supplemental Information 9 [file peerj-10-13862-s009.zip › Supplement File 5(Figure 2C Left Panel)/D3 NC.jpg]

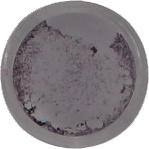

Supplement: Supplemental Information 9 [file peerj-10-13862-s009.zip › Supplement File 5(Figure 2C Left Panel)/D14 shJMJD2B.jpg]

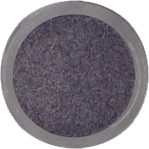

Supplement: Supplemental Information 9 [file peerj-10-13862-s009.zip › Supplement File 5(Figure 2C Left Panel)/D14 LV5-JMJD2B.jpg]

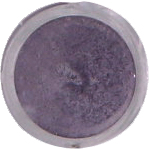

Supplement: Supplemental Information 9 [file peerj-10-13862-s009.zip › Supplement File 5(Figure 2C Left Panel)/D7 Scr.jpg]

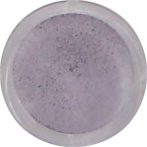

Supplement: Supplemental Information 9 [file peerj-10-13862-s009.zip › Supplement File 5(Figure 2C Left Panel)/D1 NC.jpg]

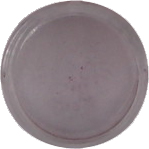

Supplement: Supplemental Information 10 [file peerj-10-13862-s010.zip › Supplement File 6(Figure 2C Right Panel)/D3 LV5-JMJD2B.jpg]

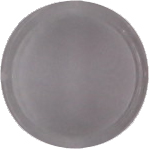

Supplement: Supplemental Information 10 [file peerj-10-13862-s010.zip › Supplement File 6(Figure 2C Right Panel)/D3 Scr.jpg]

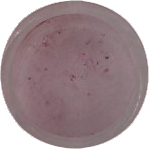

Supplement: Supplemental Information 10 [file peerj-10-13862-s010.zip › Supplement File 6(Figure 2C Right Panel)/D7 shJMJD2B.jpg]

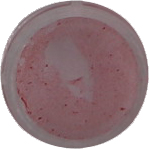

Supplement: Supplemental Information 10 [file peerj-10-13862-s010.zip › Supplement File 6(Figure 2C Right Panel)/D7 LV5-JMJD2B.jpg]

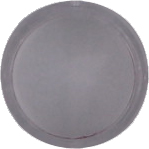

Supplement: Supplemental Information 10 [file peerj-10-13862-s010.zip › Supplement File 6(Figure 2C Right Panel)/D1 Scr.jpg]

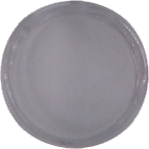

Supplement: Supplemental Information 10 [file peerj-10-13862-s010.zip › Supplement File 6(Figure 2C Right Panel)/D1 shJMJD2B.jpg]

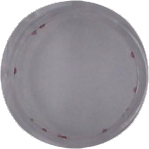

Supplement: Supplemental Information 10 [file peerj-10-13862-s010.zip › Supplement File 6(Figure 2C Right Panel)/D1 LV5-JMJD2B.jpg]

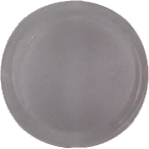

Supplement: Supplemental Information 10 [file peerj-10-13862-s010.zip › Supplement File 6(Figure 2C Right Panel)/D3 shJMJD2B.jpg]

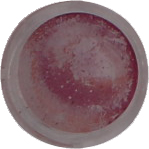

Supplement: Supplemental Information 10 [file peerj-10-13862-s010.zip › Supplement File 6(Figure 2C Right Panel)/D14 NC.jpg]

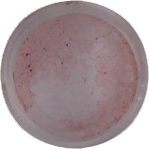

Supplement: Supplemental Information 10 [file peerj-10-13862-s010.zip › Supplement File 6(Figure 2C Right Panel)/D7 NC.jpg]

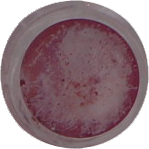

Supplement: Supplemental Information 10 [file peerj-10-13862-s010.zip › Supplement File 6(Figure 2C Right Panel)/D14 Scr.jpg]

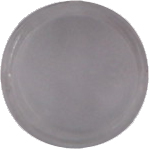

Supplement: Supplemental Information 10 [file peerj-10-13862-s010.zip › Supplement File 6(Figure 2C Right Panel)/D3 NC.jpg]

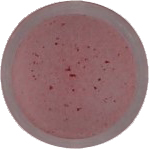

Supplement: Supplemental Information 10 [file peerj-10-13862-s010.zip › Supplement File 6(Figure 2C Right Panel)/D14 shJMJD2B.jpg]

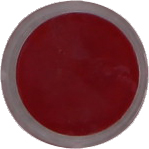

Supplement: Supplemental Information 10 [file peerj-10-13862-s010.zip › Supplement File 6(Figure 2C Right Panel)/D14 LV5-JMJD2B.jpg]

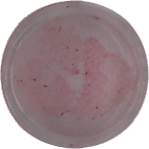

Supplement: Supplemental Information 10 [file peerj-10-13862-s010.zip › Supplement File 6(Figure 2C Right Panel)/D7 Scr.jpg]

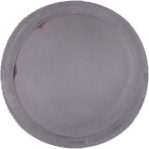

Supplement: Supplemental Information 10 [file peerj-10-13862-s010.zip › Supplement File 6(Figure 2C Right Panel)/D1 NC.jpg]

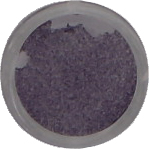

Supplement: Supplemental Information 11 [file peerj-10-13862-s011.zip › Supplement File 7(Figure 3 ALP Staining)/Control.jpg]

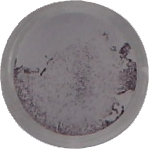

Supplement: Supplemental Information 11 [file peerj-10-13862-s011.zip › Supplement File 7(Figure 3 ALP Staining)/BOX01294.jpg]

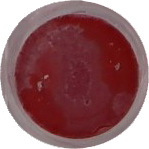

Supplement: Supplemental Information 12 [file peerj-10-13862-s012.zip › Supplement File 8(Figure 3 Alizarin Red Staining)/Control.jpg]

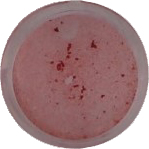

Supplement: Supplemental Information 12 [file peerj-10-13862-s012.zip › Supplement File 8(Figure 3 Alizarin Red Staining)/BIX01294.jpg]
