# Supplementary material for: Histone H3K9 demethylase JMJD2B/KDM4B promotes osteogenic differentiation of bone marrow-derived mesenchymal stem cells by regulating H3K9me2 on RUNX2
Source: PeerJ. 2022 Oct 5;10:e13862. doi: 10.7717/peerj.13862 (PMC9547583; doi:10.7717/peerj.13862)
Supplement: Supplemental Information 14 [file peerj-10-13862-s014.docx]

**Figure 1A:**

| ANOVA table | SS | DF | MS | F (DFn, DFd) | P value |
| --- | --- | --- | --- | --- | --- |
| Runx2 | | | | | |
| Treatment (between columns) | 128.4 | 4 | 32.10 | F (4, 10) = 53.22 | P<0.0001 |
| Residual (within columns) | 6.032 | 10 | 0.6032 |  |  |
| Total | 134.4 | 14 |  |  |  |
|  | | | | | |
| JMJD2B | | | | | |
| Treatment (between columns) | 259.5 | 4 | 64.88 | F (4, 10) = 37.25 | P<0.0001 |
| Residual (within columns) | 17.42 | 10 | 1.742 |  |  |
| Total | 277.0 | 14 |  |  |  |
|  | | | | | |
| FAM210A | | | | | |
| Treatment (between columns) | 248.3 | 4 | 62.08 | F (4, 10) = 91.74 | P<0.0001 |
| Residual (within columns) | 6.767 | 10 | 0.6767 |  |  |
| Total | 255.1 | 14 |  |  |  |

| Different time point  (Compare that to D0) | mRNA fold expression | repeated measure | P Value |
| --- | --- | --- | --- |
| Runx2 | | | |
| D1 | 1.206±0.2041 | 3 | >0.9999 |
| D3 | 3.165±2.162 | 3 | 0.0266 |
| D7 | 5.413±4.411 | 3 | 0.0002 |
| D14 | 8.832±7.830 | 3 | <0.0001 |
| JMJD2B | | | |
| D1 | 1.131±0.1164 | 3 | >0.9999 |
| D3 | 1.603±0.5885 | 3 | >0.9999 |
| D7 | 4.962±3.947 | 3 | 0.0175 |
| D14 | 11.92±10.91 | 3 | <0.0001 |
| FAM210A | | | |
| D1 | 1.939±0.9309 | 3 | 0.7835 |
| D3 | 2.161±1.153 | 3 | 0.4669 |
| D7 | 4.785±3.777 | 3 | 0.0009 |
| D14 | 12.15±11.14 | 3 | <0.0001 |

**Figure 2A:**

**JMJD2B**

| ANOVA summary | |
| --- | --- |
| F | 94.03 |
| P value | <0.0001 |
| R square | 0.9741 |

| Test details | mRNA relative expression | P Value |
| --- | --- | --- |
| Control vs. Scr | 1.132±0.1266 | 0.9987 |
| Control vs. shJMJD2B | 0.2970±0.7084 | 0.5936 |
| Control vs. NC | 0.9479±0.05753 | >0.9999 |
| Control vs. LV5-JMJD2B | 8.128±7.123 | <0.0001 |
| Scr vs. shJMJD2B | 0.2970±0.8350 | 0.4495 |
| Scr vs. NC | 0.9479±0.1842 | 0.9945 |
| Scr vs. LV5-JMJD2B | 8.128±6.996 | <0.0001 |
| shJMJD2B vs. NC | 0.9479±0.6508 | 0.6618 |
| shJMJD2B vs. LV5-JMJD2B | 8.128±7.831 | <0.0001 |
| NC vs. LV5-JMJD2B | 8.128±7.181 | <0.0001 |

**Figure 2D**

| Test details | Mean 1 | Mean 2 | Mean Diff. | SE of diff. | N1 | N2 | t | DF |
| --- | --- | --- | --- | --- | --- | --- | --- | --- |
|  |  |  |  |  |  |  |  |  |
| Scr - shJMJD2B |  |  |  |  |  |  |  |  |
| JMJD2B | 0.9600 | 0.1867 | 0.7733 | 0.1013 | 3 | 3 | 7.632 | 20.00 |
| Runx2 | 1.111 | 0.4545 | 0.6566 | 0.1013 | 3 | 3 | 6.481 | 20.00 |
| Osteocalcin | 1.101 | 0.5821 | 0.5185 | 0.1013 | 3 | 3 | 5.118 | 20.00 |
| Osteopontin | 1.167 | 0.4000 | 0.7667 | 0.1013 | 3 | 3 | 7.566 | 20.00 |
| Osterix | 1.120 | 0.2772 | 0.8424 | 0.1013 | 3 | 3 | 8.314 | 20.00 |
| NC-LV5-JMJD2B |  |  |  |  |  |  |  |  |
| JMJD2B | 1.040 | 12.30 | -11.26 | 0.8731 | 3 | 3 | 12.90 | 20.00 |
| Runx2 | 1.010 | 6.636 | -5.626 | 0.8731 | 3 | 3 | 6.444 | 20.00 |
| Osteocalcin | 1.013 | 6.947 | -5.934 | 0.8731 | 3 | 3 | 6.797 | 20.00 |
| Osteopontin | 1.020 | 6.657 | -5.637 | 0.8731 | 3 | 3 | 6.456 | 20.00 |
| Osterix | 1.012 | 4.632 | -3.621 | 0.8731 | 3 | 3 | 4.147 | 20.00 |

| ANOVA table | SS | DF | MS | F (DFn, DFd) | P value |
| --- | --- | --- | --- | --- | --- |
| Scr - shJMJD2B | | | | | |
| Gene x Expression | 0.09636 | 4 | 0.02409 | F (4, 16) = 1.580 | P=0.2276 |
| Gene | 0.2604 | 4 | 0.06510 | F (4, 16) = 4.271 | P=0.0154 |
| Expression | 3.797 | 1 | 3.797 | F (1, 4) = 236.9 | P=0.0001 |
| Subject | 0.06412 | 4 | 0.01603 | F (4, 16) = 1.052 | P=0.4119 |
| Residual | 0.2439 | 16 | 0.01524 |  |  |
| NC - LV5-JMJD2B |  |  |  |  |  |
| Gene x Expression | 49.11 | 4 | 12.28 | F (4, 16) = 10.55 | P=0.0002 |
| Gene | 49.91 | 4 | 12.48 | F (4, 16) = 10.72 | P=0.0002 |
| Expression | 308.7 | 1 | 308.7 | F (1, 4) = 290.4 | P<0.0001 |
| Subject | 4.253 | 4 | 1.063 | F (4, 16) = 0.9138 | P=0.4797 |
| Residual | 18.61 | 16 | 1.163 |  |  |

| Bonferroni's multiple comparisons test | Mean Diff. | 95.00% CI of diff. | Significant? | Summary | Adjusted P Value |
| --- | --- | --- | --- | --- | --- |
|  |  |  |  |  |  |
| Scr - shJMJD2B |  |  |  |  |  |
| JMJD2B | 0.7733 | 0.4850 to 1.062 | Yes | **** | <0.0001 |
| Runx2 | 0.6566 | 0.3683 to 0.9449 | Yes | **** | <0.0001 |
| Osteocalcin | 0.5185 | 0.2302 to 0.8068 | Yes | *** | 0.0003 |
| Osteopontin | 0.7667 | 0.4784 to 1.055 | Yes | **** | <0.0001 |
| Osterix | 0.8424 | 0.5541 to 1.131 | Yes | **** | <0.0001 |
| NC - LV5-JMJD2B |  |  |  |  |  |
| JMJD2B | -11.26 | -13.74 to -8.776 | Yes | **** | <0.0001 |
| Runx2 | -5.626 | -8.110 to -3.142 | Yes | **** | <0.0001 |
| Osteocalcin | -5.934 | -8.418 to -3.450 | Yes | **** | <0.0001 |
| Osteopontin | -5.637 | -8.121 to -3.153 | Yes | **** | <0.0001 |
| Osterix | -3.621 | -6.105 to -1.137 | Yes | ** | 0.0025 |

**Figure 3A**

| Bonferroni's multiple comparisons test | Mean Diff. | 95.00% CI of diff. | Significant? | Summary | Adjusted P Value |  |
| --- | --- | --- | --- | --- | --- | --- |
| IgG vs. Scr | -0.5241 | -1.285 to 0.2365 | No | ns | 0.3323 | A-B |
| IgG vs. si-JMJD2B | -0.1494 | -0.9100 to 0.6112 | No | ns | >0.9999 | A-C |
| IgG vs. NC | -0.5423 | -1.303 to 0.2183 | No | ns | 0.2869 | A-D |
| IgG vs. LV5-JMJD2B | -3.419 | -4.180 to -2.659 | Yes | **** | <0.0001 | A-E |
| Scr vs. si-JMJD2B | 0.3747 | -0.3859 to 1.135 | No | ns | >0.9999 | B-C |
| Scr vs. NC | -0.01819 | -0.7788 to 0.7424 | No | ns | >0.9999 | B-D |
| Scr vs. LV5-JMJD2B | -2.895 | -3.656 to -2.135 | Yes | **** | <0.0001 | B-E |
| si-JMJD2B vs. NC | -0.3928 | -1.153 to 0.3677 | No | ns | 0.9407 | C-D |
| si-JMJD2B vs. LV5-JMJD2B | -3.270 | -4.030 to -2.509 | Yes | **** | <0.0001 | C-E |
| NC vs. LV5-JMJD2B | -2.877 | -3.638 to -2.116 | Yes | **** | <0.0001 | D-E |

| Test details | Mean 1 | Mean 2 | Mean Diff. | SE of diff. | n1 | n2 | t | DF |
| --- | --- | --- | --- | --- | --- | --- | --- | --- |
| IgG vs. Scr | 0.02697 | 0.5511 | -0.5241 | 0.2124 | 3 | 3 | 2.468 | 10 |
| IgG vs. si-JMJD2B | 0.02697 | 0.1764 | -0.1494 | 0.2124 | 3 | 3 | 0.7036 | 10 |
| IgG vs. NC | 0.02697 | 0.5692 | -0.5423 | 0.2124 | 3 | 3 | 2.553 | 10 |
| IgG vs. LV5-JMJD2B | 0.02697 | 3.446 | -3.419 | 0.2124 | 3 | 3 | 16.10 | 10 |
| Scr vs. si-JMJD2B | 0.5511 | 0.1764 | 0.3747 | 0.2124 | 3 | 3 | 1.764 | 10 |
| Scr vs. NC | 0.5511 | 0.5692 | -0.01819 | 0.2124 | 3 | 3 | 0.08565 | 10 |
| Scr vs. LV5-JMJD2B | 0.5511 | 3.446 | -2.895 | 0.2124 | 3 | 3 | 13.63 | 10 |
| si-JMJD2B vs. NC | 0.1764 | 0.5692 | -0.3928 | 0.2124 | 3 | 3 | 1.850 | 10 |
| si-JMJD2B vs. LV5-JMJD2B | 0.1764 | 3.446 | -3.270 | 0.2124 | 3 | 3 | 15.40 | 10 |
| NC vs. LV5-JMJD2B | 0.5692 | 3.446 | -2.877 | 0.2124 | 3 | 3 | 13.55 | 10 |

**Figure 3C**

| Bonferroni's multiple comparisons test | Mean Diff. | 95.00% CI of diff. | Significant? | Summary | Adjusted P Value |
| --- | --- | --- | --- | --- | --- |
|  |  |  |  |  |  |
| Control - BIX01294 |  |  |  |  |  |
| JMJD2B | 0.4010 | 0.2711 to 0.5310 | Yes | **** | <0.0001 |
| Runx2 | 0.8914 | 0.7614 to 1.021 | Yes | **** | <0.0001 |

| Test details | Mean 1 | Mean 2 | Mean Diff. | SE of diff. | N1 | N2 | t | DF |
| --- | --- | --- | --- | --- | --- | --- | --- | --- |
| Control - BIX01294 |  |  |  |  |  |  |  |  |
| JMJD2B | 1.001 | 0.5999 | 0.4010 | 0.04725 | 3 | 3 | 8.489 | 8.000 |
| Runx2 | 1.002 | 0.1108 | 0.8914 | 0.04725 | 3 | 3 | 18.87 | 8.000 |

**Figure 3F**

| Bonferroni's multiple comparisons test | Mean Diff. | 95.00% CI of diff. | Significant? | Summary | Adjusted P Value |  |
| --- | --- | --- | --- | --- | --- | --- |
| JMJD2B | | | | | | |
| NC vs. LV5-JMJD2B | -2.580 | -4.570 to -0.5902 | Yes | * | 0.0159 | A-B |
| NC vs. LV5-JMJD2B | -3.406 | -5.396 to -1.416 | Yes | ** | 0.0040 | A-C |
| LV5-JMJD2B vs. LV5-JMJD2B | -0.8257 | -2.816 to 1.164 | No | ns | 0.6645 | B-C |
| Runx2 | | | | | | |
| NC vs. LV5-JMJD2B | -1.872 | -2.944 to -0.7998 | Yes | ** | 0.0036 | A-B |
| NC vs. LV5-JMJD2B | -4.496 | -5.568 to -3.424 | Yes | **** | <0.0001 | A-C |
| LV5-JMJD2B vs. LV5-JMJD2B | -2.624 | -3.696 to -1.552 | Yes | *** | 0.0006 | B-C |

| Test details | Mean 1 | Mean 2 | Mean Diff. | SE of diff. | n1 | n2 | t | DF |
| --- | --- | --- | --- | --- | --- | --- | --- | --- |
| JMJD2B |  |  |  |  |  |  |  |  |
| NC vs. LV5-JMJD2B | 1.000 | 3.580 | -2.580 | 0.6053 | 3 | 3 | 4.262 | 6 |
| NC vs. LV5-JMJD2B | 1.000 | 4.406 | -3.406 | 0.6053 | 3 | 3 | 5.626 | 6 |
| LV5-JMJD2B vs. LV5-JMJD2B | 3.580 | 4.406 | -0.8257 | 0.6053 | 3 | 3 | 1.364 | 6 |
| Runx2 |  |  |  |  |  |  |  |  |
| NC vs. LV5-JMJD2B | 1.006 | 2.878 | -1.872 | 0.3261 | 3 | 3 | 5.740 | 6 |
| NC vs. LV5-JMJD2B | 1.006 | 5.501 | -4.496 | 0.3261 | 3 | 3 | 13.79 | 6 |
| LV5-JMJD2B vs. LV5-JMJD2B | 2.878 | 5.501 | -2.624 | 0.3261 | 3 | 3 | 8.046 | 6 |
